# Bracket Transfer Accuracy with the Indirect Bonding Technique—A Systematic Review and Meta-Analysis

**DOI:** 10.3390/jcm11092568

**Published:** 2022-05-04

**Authors:** Hisham Sabbagh, Yeganeh Khazaei, Uwe Baumert, Lea Hoffmann, Andrea Wichelhaus, Mila Janjic Rankovic

**Affiliations:** 1 Department of Orthodontics and Dentofacial Orthopedics, University Hospital, LMU Munich, Goethestrasse 70, 80336 Munich, Germany; uwe.baumert@med.uni-muenchen.de (U.B.); lea.hoffmann@med.uni-muenchen.de (L.H.); kfo.sekretariat@med.uni-muenchen.de (A.W.); mila.janjic@med.uni-muenchen.de (M.J.R.); 2 Statistical Consultation Unit, StaBLab, Department of Statistics, LMU Munich, 80799 Munich, Germany; yeganekhazaei@gmail.com

**Keywords:** bracket bonding, indirect bonding, orthodontic brackets, transfer accuracy, bracket positioning, bonding accuracy, bonding tray

## Abstract

Purpose: To investigate the bracket transfer accuracy of the indirect bonding technique (IDB). Methods: Systematic search of the literature was conducted in PubMed MEDLINE, Web of Science, Embase, and Scopus through November 2021. Selection Criteria: In vivo and ex vivo studies investigating bracket transfer accuracy by comparing the planned and achieved bracket positions using the IDB technique were considered. Information concerning patients, samples, and applied methodology was collected. Measured mean transfer errors (MTE) for angular and linear directions were extracted. Risk of bias (RoB) in the studies was assessed using a tailored RoB tool. Meta-analysis of ex vivo studies was performed for overall linear and angular bracket transfer accuracy and for subgroup analyses by type of tray, tooth groups, jaw-related, side-related, and by assessment method. Results: A total of 16 studies met the eligibility criteria for this systematic review. The overall linear mean transfer errors (MTE) in mesiodistal, vertical and buccolingual direction were 0.08 mm (95% CI 0.05; 0.10), 0.09 mm (0.06; 0.11), 0.14 mm (0.10; 0.17), respectively. The overall angular mean transfer errors (MTE) regarding angulation, rotation, torque were 1.13° (0.75; 1.52), 0.93° (0.49; 1.37), and 1.11° (0.68; 1.53), respectively. Silicone trays showed the highest accuracy, followed by vacuum-formed trays and 3D printed trays. Subgroup analyses between tooth groups, right and left sides, and upper and lower jaw showed minor differences. Conclusions and implications: The overall accuracy of the indirect bonding technique can be considered clinically acceptable. Future studies should address the validation of the accuracy assessment methods used.

## 1. Introduction

The straight-wire technique derived from the works of Andrews [1,2] is the most commonly used technique in fixed orthodontic treatment [3]. In this technique, the ideal placement of the brackets is of utmost importance [4,5,6,7]. Positioning errors necessitate the repositioning of brackets or the insertion of additional compensatory bends [4,8,9,10,11,12,13,14] and increase the number of visits and the treatment duration [5], thus compromising treatment efficiency.

Clinically, brackets can be positioned directly with an instrument or indirectly with a transfer tray. Indirect bonding (IDB) was first proposed in 1972 [15] and has since been used mainly to improve accuracy through pre-planning the ideal bracket position [7]. Numerous studies have shown that IDB can increase the precision of bracket placement [8,16,17,18,19,20], but neither the direct nor the indirect technique achieves ideal clinical results, and readjustments remain necessary [3,21,22,23,24]. 

More recently, with the introduction of software for virtual treatment planning and workflows for additive transfer tray manufacturing for IDB, another approach for ideal bracket placement was introduced [25]. By calculating and visualizing the tooth movements resulting from the application of the virtually positioned brackets, adjustments can be made to realize the treatment objectives in the digital setup [13]. Accurate clinical implementation of the planned bracket positions is crucial in this method to achieve the virtually simulated alignment [26].

A growing number of studies have addressed the topic of IDB accuracy [26,27,28]. There is, however, great variability in the reported results between studies, which might be due to underlying methodological or clinical heterogeneity. Thus, the aim of this study was to synthesize the findings and assess the accuracy of the IDB technique, focusing not only on the overall accuracy of the method or different types of indirect bonding trays but also taking into account methodological and clinical aspects such as the method used to evaluate accuracy, and tooth-type-specific and jaw-related differences.

## 2. Materials and Methods

This systematic review was conducted according to the “Preferred Reporting Items for a Systematic Review and Meta-analysis of Diagnostic Test Accuracy Studies” (PRISMA-DTA) statement [29] and registered at the PROSPERO platform (registration number: CRD42021243227). The PICO model (problem/patient, intervention, comparison, outcome) was followed to define the research question and eligibility criteria [30]. Detailed information on how this model influenced the study design, and the definition of each PICO element can be found in Supplementary Table S1.

### 2.1. Eligibility Criteria

Prospective and retrospective in vivo and ex vivo studies investigating bracket transfer accuracy by comparing the planned and achieved bracket positions for buccal bracket bonding were considered. The following eligibility criteria were applied. (1) At least one of the measurements in the linear (mesiodistal, buccolingual, vertical) and/or angular (angulation, rotation, torque) directions was reported. (2) Actual status of the bracket position was confirmed by comparing it to the planned bracket position. Studies assessing lingual bracket bonding accuracy were not considered for inclusion. Only studies published in English were considered, and the last update of the search according to the search strategy was performed on 1 November 2021.

### 2.2. Literature Search and Study Selection Process

Based on the research question and the aforementioned eligibility criteria, a search strategy was developed. Following the Cochrane recommendations for studies dealing with very specific topics, such as indirect bonding, we applied the following concept and broke it into three sub-concepts in order to create our search strategy [31] (Table 1).

This template was applied to four bibliographic databases (PubMed, Embase, Web of Science, and Scopus) with specific adaptations for each bibliographic database (Table 2). Sets of records from each database were downloaded to the bibliographic software package EndNote X9 (Clarivate Analytics, Philadelphia, PA, USA) and merged into one core database in order to remove duplicate records.

All records identified by the searches were primarily checked on the basis of title and abstract. Full texts of the records identified as relevant were then downloaded and checked for meeting the eligibility criteria. The articles that did not meet the predefined inclusion criteria after the full-text assessment were excluded from further examination. The whole literature screening process was conducted independently in parallel by two of the authors (H.S, M.J.R). The Cohen’s K coefficient for agreement between the two reviewers was 0.89. Any doubts or disagreements were solved by discussion.

### 2.3. Data Extraction

Data from the included studies were extracted by both reviewers in specially prepared data extraction sheets. Any differences in extracted data were resolved through discussion until reaching a consensus. 

Briefly, the following information was extracted from papers: author and year of publication, study design, number of assessed teeth (incisors, canines, premolars, molars); patient information in case of in vivo studies; IDB technique used (double polyvinyl siloxane (double-PVS); double vacuum-form (double-VF), polyvinyl siloxane vacuum-form (PVS-VF), polyvinyl siloxane putty (PVS-putty), and single vacuum-form (single-VF)); type of brackets used in the study; method for measuring transfer accuracy (digital photography, calipers, CBCT, 3D-scan and superimposition); mean transfer errors (MTE) in linear (mesiodistal, buccolingual, vertical) and angular (angulation, rotation, torque) directions expressed in millimeters (mm) and degrees (°). All corresponding authors of the included studies were contacted to provide the complete data sets or additional data if available. For included studies reporting data only graphically [10,32], data were collected using a data extraction software (WebPlotDigitzer, Version 4.4, Pacifica, CA, USA) as described and validated by Drevon et al. [33]. All data were later transferred to Excel spreadsheets (Excel 2010, Microsoft Corporation, Redmond, WA, USA). The data transfer was checked twice by both reviewers involved before further analysis.

### 2.4. Risk of Bias Assessment in Included Studies

In this review, an adapted risk of bias (RoB) assessment tool was used (Supplementary Table S3) [34,35]. The tool contained four domains (selection bias; reference test bias; verification bias; outcome bias), each of them included items that cover different sources of bias. One of the following three modalities was used to judge the RoB in the primary studies: high, low, or unclear risk of bias. The category “unclear RoB” was applied whenever incomplete details or no information could be found in the study. RoB assessment was performed independently by the two of the authors (H.S., M.J.R).

### 2.5. Meta-Analysis and Synthesis of Results

Meta-analysis was performed using R Statistical Software (Version 4.1.1, R Core Team, Vienna, Austria) according to published procedures [36,37]. To be included in the meta-analysis, the sample size and the mean and standard deviation (SD) of the bracket transfer error expressed in millimeters (mm) or degrees (°) were required.

The overall mean transfer errors (MTE) and further subgroup analyses in linear (mesiodistal, buccolingual, vertical) and angular (angulation, rotation, torque) directions were performed in the following categories: overall MTE; tooth group related MTE; jaw-related MTE (left vs. right/upper vs. lower); MTE in relation to accuracy assessment method; MTE in relation to the type of IDB tray.

Data wrangling and manipulation were performed using the statistical packages “tidyverse” [37], “dplyr” [38], and “ggplot2” [39]. Meta-analytic syntheses and further investigations were performed by “meta” and “dmetar” in RStudio (Rstudio Inc., Boston, MA, USA) [36,40]. Effect sizes of the overall MTE and subgroup analyses were calculated by the metamean function provided by “meta” and are reported in Table 2. Heterogeneity was assessed using Cochran’s Q and *I*^2^-statistics. A random-effects model was retained to pool effect sizes to better account for the differences in design amongst the included studies for both overall category and subgroups analysis. The restricted maximum likelihood estimator was used to calculate the heterogeneity variance τ^2^ [41]. Knapp–Hartung adjustments were used to calculate the confidence interval around the pooled effect [42]. To investigate publication bias, funnel plots were prepared using the functionalities of the “meta” package. Additionally, drapery plots were produced based on *p*-value functions.

## 3. Results

### 3.1. Literature Search Results

**Figure 1:**
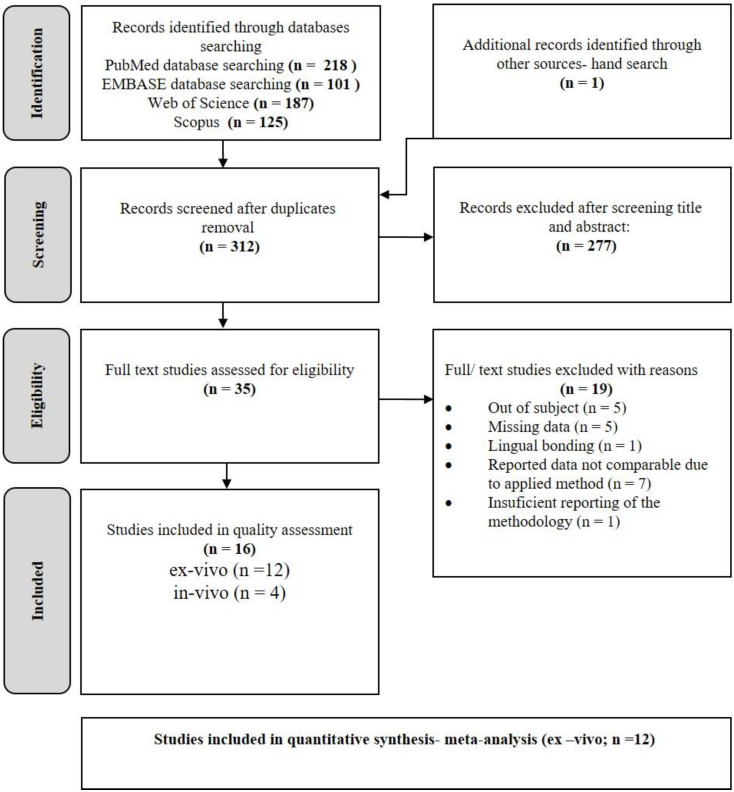
Flow diagram of information through the different phases of a systematic review according to Preferred Reporting Items for Systematic Reviews and Meta-Analyses (study selection process).

The PRISMA workflow illustrating the whole study selection process is summarized in Figure 1. The electronic search resulted in 218 records from PubMed, 187 records from Web of Science, 101 records from EMBASE, and 125 records from Scopus.

After duplicate elimination, altogether, 312 studies were identified. Upon checking the titles and abstracts of the identified records, 35 studies were selected for full-text reading. Studies that did not meet the eligibility criteria (*n* = 19) were excluded from further assessment, and the reasoning is summarized in Supplementary Table S2. Additionally, one more study was selected for inclusion by cross-checking the reference lists of literature selected for inclusion, resulting in a total number of 16 included studies. For two publications [7,28], additional data that was not included in the original manuscripts were provided by the respective authors.

### 3.2. Results of the Risk of Bias Assessment

**Figure 2:**
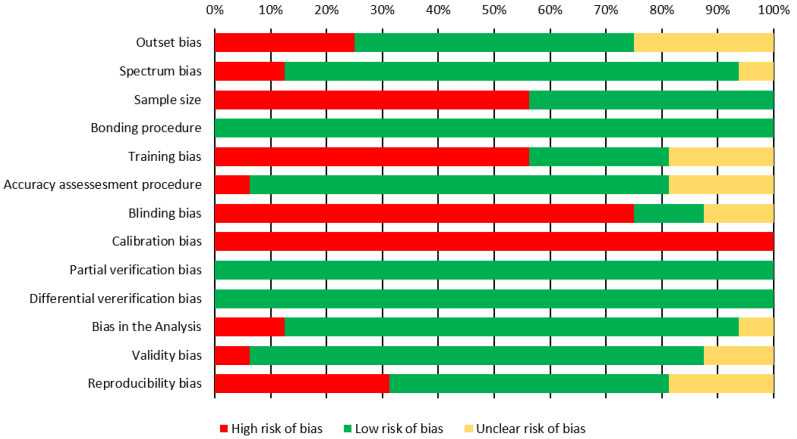
Overview of the overall RoB among different domains and items.

The overall risk of bias (RoB) of the different domains and items is given in Figure 2. Results of the RoB assessment of the individual studies are available in Supplementary Table S5. In eight studies, indirect bracket placement might have been affected by malocclusions, such as severe crowding or rotations, or no such information was provided [7,18,28,43,44,45,46,47]. Only seven studies provided information on sample size calculation [26,27,28,44,46,47,48]. Three studies considered only specific tooth groups in their investigations [44,45,48]. Nine studies did not report the experience and training of the bonding clinicians or indicated low experience [7,10,26,32,43,44,45,47,48], and in three studies, the bonding clinicians’ experience was unclear based on the provided information [27,46,49].

None of the included studies provided information on calibration, and only two studies provided information on blinding of the examiners [47,48]. Eight studies had a high or unclear risk of bias due to an insufficient method for reproducibility assessment or insufficient reporting [7,10,18,46,47,48,49,50].

### 3.3. Study Characteristics and Results of Individual Studies

The characteristics of the studies included for quality assessment are illustrated in Table 3.

#### 3.3.1. Study Characteristics and Results of the In Vivo Studies Not Included in the Quantitative Synthesis

Four in vivo studies were eligible for quality assessment after full-text reading [7,26,47,48]. Two of the studies investigated the bracket transfer accuracy of 3D printed trays [26,47], one of which compared 3D printed trays to silicone trays [47]. The other two studies investigated silicone trays [46] and vacuum-formed trays [48]. All included in vivo studies investigated the accuracy of bracket transfer with conventional brackets. In these studies, three different methods were used to evaluate accuracy: CBCT and software [7], photography [48], and scans and software [26,47]. Due to the small number of in vivo studies with different study characteristics, they were not included in the quantitative synthesis. The reported linear mean transfer errors ranged from 0.001 to 0.050 mm. The angular mean transfer errors ranged from 0.001 to 1.757°. The full extracted data is available in in Supplementary Tables S4.1–S4.6.

#### 3.3.2. Study Characteristics of the Ex vivo Studies Included in the Quantitative Synthesis

A total of 12 ex vivo studies were eligible for quality assessment after full text reading [10,18,27,28,32,43,44,45,46,49,50,51]. Of these, 7 studies investigated 3D printed trays [27,28,32,43,44,45,50], while 5 investigated vacuum-formed trays [10,32,46,49,51], and 4 studies investigated silicone trays [10,18,27,46], with 4 of the 12 included studies comparing more than 1 material group [10,27,32,46]. Three studies used self-ligating brackets for indirect bonding [43,44,50]. The most common method of analysis was the use of scans and software (*n* = 9) [10,27,28,32,43,44,45,46,49,50], followed by methods using photography (*n* = 3) [10,18,51].

### 3.4. Results of the Meta-Analysis

**Figure 3:**
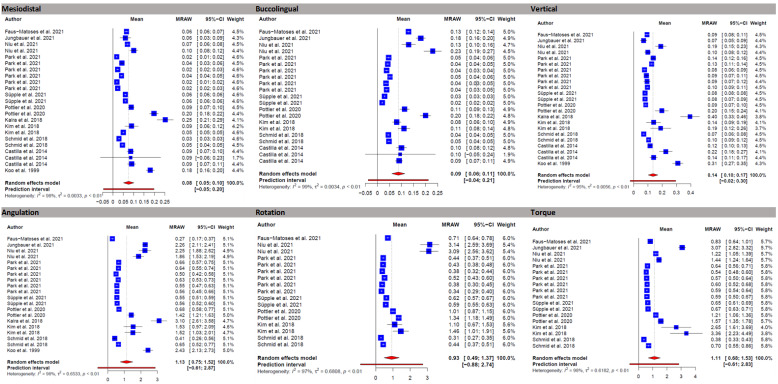
Forest plots showing overall linear and angular mean bracket transfer errors (MTE) [10,18,27,28,32,43,44,45,46,49,50,51].

The results of the meta-analysis are summarized in Table 4. The overall linear and angular mean bracket transfer errors are shown in forest plots in Figure 3. The full data sets, including forest plots, drapery plots, and funnel plots for different analysis groups, are available in Supplementary Tables S6.1–S8.12.

### 3.5. Linear Mean Transfer Errors

Overall linear mean transfer errors (MTE) in mesiodistal, buccolingual, and vertical directions were 0.08 mm, 0.09 mm, and 0.14 mm, respectively (Table 4). A comparison of linear MTE between different tooth groups revealed that IDB was less accurate in the incisor group, with an MTE of 0.14 mm in the buccolingual direction and 0.15 mm in the vertical direction. No significant differences could be observed in a comparison of IDB transfer accuracy between left and right sides in all three linear directions. The comparison between the upper and lower jaw showed slightly higher bracket transfer accuracy in the upper jaw in the mesiodistal and vertical directions (MTE 0.10 mm, 0.18 mm), whereas accuracy in the buccolingual direction was lower than in the lower jaw (MTE 0.09 mm). Among the different types of IDB trays, 3D printed trays showed the highest accuracy in the mesiodistal (MTE 0.06 mm) and vertical directions (MTE 0.12 mm) but the lowest accuracy in the buccolingual dimension (MTE 0.10 mm). 

In studies that used photography as a method to assess accuracy, the MTE was higher in the mesiodistal and vertical directions (MTE 0.12, 0.22) than in studies that used 3D assessment methods (MTE 0.06, 0.11).

### 3.6. Angular Mean Transfer Errors

Overall angular mean transfer errors (MTE) regarding angulation, rotation, and torque were 1.13°, 0.93°, and 1.11°, respectively. Compared to the other tooth groups, molar tubes showed the highest transfer accuracy in rotation (MTE 0.69°) but the lowest in torque (MTE 2.29°). In the premolar group, the highest accuracy was observed for angulation (MTE 0.13°) and torque (0.95°), while rotation (MTE 1.46°) showed the lowest accuracy compared to the other tooth groups. The comparisons between the left and right sides, between upper and lower jaws, and between 3D accuracy assessment and photography could only be partially evaluated on the basis of the available data for angular values. 

Studies that used photography as a method showed a lower bracket transfer accuracy for angulation (MTE 2.74°) compared to studies that used 3D assessment (MTE 0.95°). IDB showed higher accuracy regarding angulation in the upper jaw (MTE 1.26°) but lower accuracy for rotation (MTE 0.59°) and torque (0.73°). For 3D printed trays, higher torque deviations were observed (MTE 1.42°) than for other types of IDB trays. For silicone trays, the highest accuracy was observed for angulation (MTE 0.66°) and torque (0.79°).

## 4. Discussion

### 4.1. Overall

In this study, the available literature on the indirect bonding technique was systematically reviewed regarding the accuracy of bracket transfer and differences among available methods to draw conclusions on methodological and clinical aspects. 

The results of the meta-analysis showed an overall bracket transfer accuracy for the indirect bonding technique between 0.08 and 0.14 mm for linear and 0.93° and 1.13° for angular deviations, respectively. As there are no evidence-based limits for clinically acceptable bracket position deviations in the literature, most studies refer to the professional standards of the American Board of Orthodontics of 0.5 mm for linear and 2° for angular deviations [7,10,26,27,32,45,46,49,52]. However, these limits apply by definition to deviations of tooth positions. As full slot engagement with orthodontic archwires cannot be achieved in the straight-wire technique [27,53,54,55], exceeding these limits cannot be equated with malpositioning of the associated teeth. In view of these considerations and the limitations due to the current reference standard, the overall accuracy of the indirect bonding technique can be considered clinically acceptable.

Regarding linear deviations, a higher mean transfer error was observed for the vertical direction than for the mesiodistal and buccolingual directions, which is in line with previous studies [10,46,48,56] and mostly attributed by the authors to misfit phenomena of the indirect bonding trays. Therefore, it has been proposed to increase the distance between the dentition and the transfer tray by adapted designs to improve the fit and reduce vertical deviations [26]. Angular deviations (torque, rotation, and angulation), on the other hand, showed comparable values, although deviations for torque were reported to be highest in previous studies [26,27,28,32,57]. It is possible that the angular deviations are more dependent on the amount of adhesive, tray material, and tray design, and therefore different results are observed in the respective studies depending on the method used [28].

### 4.2. Tooth Groups

Subgroup analysis by tooth groups showed the lowest angular deviations in the premolar group for all directions but rotation, where transfer was most accurate for molar attachments. Interestingly, linear bracket transfer errors were higher for anterior teeth (incisors and canines) than for posterior teeth (premolars and molars), contrary to previous findings [7,32,57]. 

The high rotational accuracy of molar attachments could be explained by the larger mesio-distal extension compared to the attachments of other tooth groups. However, the overall differences between the tooth groups in the included ex vivo studies were small and likely to be clinically negligible.

### 4.3. Side Differences and Differences between Upper and Lower Jaw

It is considered that one of the advantages of the indirect bonding technique is that it allows for consistent accuracy in bracket placement, regardless of the practitioner’s handedness or direction of viewing direction and sitting position [32]. However, only a few studies that met the inclusion criteria provided accuracy data separately for the right and left sides, and for the upper and lower jaws, so only limited conclusions can be drawn. Based on data from five studies included in the meta-analysis, no differences in bracket transfer accuracy were found between the right and left sides. In contrast, slightly higher bracket transfer accuracy was found for the upper jaw than for the lower jaw. This result should be interpreted with caution, as it may be biased by the limited number of included studies providing accuracy data for the lower jaw.

### 4.4. Tray Materials

Regarding tray materials, silicone trays represent the reference in terms of accuracy [10,27,46,56]. In previous studies that compared 3D printed trays with other methods, 3D printed trays were found to have a higher bracket transfer accuracy than vacuum-formed trays [32] but lower than silicone trays [27,57]. Interestingly, in this study, 3D printed trays showed lower MTE in the mesiodistal and vertical directions and in angulation compared with the other tray material groups. The use of 3D-printed trays has been suggested to potentially increase treatment efficiency by improving treatment planning through digital setup, treatment simulation, implementation of 3D imaging data such as CBCT or MRI, and by simplifying the laboratory process [11,13,26]. However, further research is necessary to determine the influence of factors like tray design [26,32], material used [57], and manufacturing process.

### 4.5. Accuracy Assessment Method

Included studies using photography as a method for accuracy assessment showed a higher MTE in comparison to studies using 3D assessment. 3D assessment methods for bracket transfer accuracy using scanners or CBCT have been proposed to generally achieve higher accuracy [26,27]. However, most of the included studies did not adequately evaluate the accuracy of the assessment workflow or did not report all relevant reliability data. The sole use of Dahlberg’s formula, intraclass correlation coefficient, or analysis using a paired *t*-test for reliability reporting in orthodontic research is not adequate [58]. Furthermore, Jungbauer et al. [28] questioned the suitability of intraoral scanners for accurately determining the bracket transfer accuracy because of significant artifacts on scanned brackets and low intra- and inter-rater reliability in their study. The use of photographic methods, on the other hand, has the disadvantage that not all deviation directions can be evaluated. 

Accurate registration of achieved bracket positions is a technical challenge, which may partly explain why ex vivo studies are predominantly available on this topic. Despite the methodological limitations discussed, scans, photographs, and micro-Ct data appear to be suitable, in principle, for the assessment of IDB accuracy. However, adequate validation of the accuracy assessment method is required to reduce the risk of bias in future studies and to support more targeted research, in which the accuracy values obtained may be useful to practitioners with respect to the clinical protocols. Finally, all relevant data should be made available in future studies to allow for more comprehensive reviews. 

## 5. Strengths and Limitations

To the authors’ knowledge, to date, no systematic review has comprehensively addressed the assessment of bracket transfer accuracy, including methodological and clinical aspects of the IDB method. In addition, the number of available studies without standards on the methodological aspects of assessment, validation, and reporting is increasing, which limits the validity and generalizability of the results. However, it should not be neglected that conducting a meta-analysis with a small number of available studies is also subject to limitations.

As we anticipated considerable between-study heterogeneity, a random-effects model was used to pool effect sizes. The results of τ^2^, *I*^2^-statistics, and the corresponding *p*-values indicated that between-study heterogeneity existed in most of the categories and that the use of a random-effects model was appropriate. Nevertheless, the results of the subgroup analyses should be interpreted with caution. The statistical power of small subgroups is limited because the effects are smaller than in the meta-analysis performed for the overall group [36].

Moreover, a controversy around *p*-value and the sole use of forest plots to visualize results of meta-analyses is rising [59]. Forest plots can only display confidence intervals with the assumption of a fixed significance threshold (*p*-value < 0.05). Therefore, in this study, we used drapery plots in addition to forest plots. Drapery plots that present the *p*-value function for all individual studies are suggested as being complementary figures to forest plots for presentation and interpretation of the results of a meta-analysis, specifically with a low number of studies, such as our study [60]. This prevents researchers from solely relying on the *p*-value < 0.05 significance threshold when interpreting the results. The resulting drapery plots are documented in Supplementary Tables S8.1–S8.12. 

Due to the low number of in vivo studies (*n* = 4), with significant differences in the applied methodologies and an extensive and sometimes contradictory range of published results, a meta-analysis could only be carried out for ex vivo studies. The bracket transfer accuracy in in vivo settings could be lower due to limited accessibility of the oral cavity [46], moisture control and soft-tissue interference [32], patient management [45], malocclusion [10], and other factors. Therefore, further methodologically sound in vivo studies are necessary to evaluate the accuracy of the indirect bonding technique in clinical settings.

## 6. Clinical Implications

Accurate bracket placement is essential for effective and efficient treatment with fixed orthodontic appliances [1,7,10,26]. However, due to the complexity of the various clinical and technical aspects of bracket bonding and despite the large number of studies dealing with this topic, there is disagreement on the most appropriate techniques or methods [16]. Objective evidence from well-conducted, prospective, randomized clinical trials is still lacking [16,61]. 

The findings of this systematic review suggest that indirect bonding as a technique allows achieving planned bracket positions with high overall accuracy, even though the results addressed herein are not sufficient to reflect all of the various clinical aspects. It was shown that using indirect bonding, tooth-type-specific and jaw-related differences appear to have a rather negligible overall influence on accuracy. In contrast to previously published studies [27,47], indirect bracket positioning with 3D printed trays generally appears to be as accurate as silicone trays. Therefore, the selection of one of these techniques could be based on preferences or criteria such as fabrication cost, time, or cost-effectiveness, even though the reduced number of manufacturing steps and further advances in computer-aided technologies will likely favor 3D-printed trays [61].

Indirect bonding remains more time and cost-consuming overall than direct bonding due to the laboratory process required, although it has been shown to reduce clinical chair time [8,61,62]. Further research is needed to evaluate the correlation between the accuracy of bracket placement and the need for compensatory bends, bracket repositioning, and reduction in total treatment time, given the conflicting results to date [5,61,63,64].

## 7. Conclusions

The results of this meta-analysis indicate a generally precise implementation of planned bracket positions in the indirect bonding technique. Among tray materials, silicone trays and 3D printed trays showed higher accuracy compared to vacuum-formed trays. Subgroup analyses between tooth groups, right and left sides, and upper and lower jaw showed only minor differences. In addition to the main objectives, future studies should address the validation of the accuracy assessment methods and provide complete data sets, including adequate reliability data, to reduce the risk of bias.

## Figures and Tables

**Table 1:** Concept of the search strategy.

Domain	Search Term
Field	orthodont*
	AND
Intervention	bonding
	AND
Outcome	positioning differences OR accuracy OR transfer accuracy OR ideal bracket placement OR accurate bracket positioning OR accurat*

**Table 2:** List of adapted search strategies used for different databases and number of identified records.

Database	Search Strategies	Results
PubMed	orthodont* [All Fields] AND bonding [All Fields] AND ((positioning [All Fields] differences [All Fields]) OR accuracy [All Fields] OR (transfer [All Fields] accuracy [All Fields]) OR (ideal [All Fields] bracket [All Fields] placement [All Fields]) OR (accurate bracket [All Fields] positioning [All Fields]) [All Fields] OR accurat* [All Fields])	218
Embase	orthodont*.mp. AND bonding.mp. AND ((positioning differences).mp. OR accuracy.mp. OR (transfer accuracy).mp. OR (ideal bracket placement).mp. OR (accurate bracket positioning).mp. OR accurat*.mp.)	101
Web of Science	orthodont* AND bonding AND (positioning differences OR accuracy OR transfer accuracy OR ideal bracket placement OR accurate bracket positioning OR accurat*)	187
Scopus	TITLE-ABS-KEY (orthodont* AND bonding AND (“positioning differences” OR “positioning difference” OR accurac* OR “transfer accuracy” OR “ideal bracket placement” OR “ideal bracket placements” OR “accurate bracket positioning” OR accurat*))	125
	Total	312

**Table 3:** Study characteristics of the included studies.

Study Details		Sample Details		Bonding Procedure (Indirect)						Transfer Accuracy Assessment	
Author (Year)	Type of Study	Sample Size Calculation/Method	No. of Assessed Brackets	No. of Bonding Clinicians	Type of IDB Tray	Bonded Subject (s)/Object (s)	Data for Reference Model(s)	Tray Construction	Type of Brackets	No. of Examiners	Measuring Method
			Total/I/C/PM/M								
Jungbauer et al. [28], 2021	ex vivo	Yes	280/80/40/80/80	NR	3D printed (soft)	bonding on plaster or printed model	impression	Virtual model, Rapid prototyping	conventional	NR	Scan + Software
			280/80/40/80/80		3D printed (hard)	bonding on plaster or printed model	impression	Virtual model, Rapid prototyping			
Park et al. [43], 2021	ex vivo	No	506/147/79/122/158	1	3D printed	bonding on plaster or printed model	model scan	Virtual model, Rapid prototyping	self-ligating	1	Scan + Software
Park et al. [44], 2021	ex vivo	Yes	225/NR	1	3D printed	bonding on plaster or printed model	model scan	Virtual model, Rapid prototyping	self-ligating	1	Scan + Software
Faus-Matoses et al. [50], 2021	ex vivo	No	335/NR	NR	3D printed	bonding on plaster or printed model	scan	Virtual model, Rapid prototyping	self-ligating	NR	Scan + Software
Niu et al. [32], 2021	ex vivo	Yes	108/37/10 19/32/20	NR	3D printed	bonding on plaster or printed model	intraoral scan	Virtual model, Rapid prototyping	conventional	NR	Scan + Software
		Yes	104/31/18/35/20	NR	Vacuum Form	bonding on plaster or printed model	intraoral scan	Virtual model, Rapid prototyping	conventional	NR	Scan + Software
Süpple et al. [49], 2021	ex vivo	No	729/210/107/207/205	NR	Vacuum Form (group H)	bonding on plaster or printed model	scan	Virtual model, Rapid prototyping	conventional	NR	Scan + Software
		No	724/209/106/206/203		Vacuum Form	bonding on plaster or printed model	scan	Model and laboratory process	conventional	NR	Scan + Software
					(group V)						
Pottier et al. [27], 2020	ex vivo	Yes	97/38/20/39/-	1	Silicone	bonding on plaster or printed model	intraoral scan	Virtual model, Rapid prototyping	conventional	1	Scan + Software
		Yes	98/40/19/39/-		3D printed tray	bonding on plaster or printed model	intraoral scan	Virtual model, Rapid prototyping	conventional	1	Scan + Software
Kalra et al. [51], 2018	ex vivo	No	100/20/10/20/0	5	Vacuum Form	bonding on plaster or printed model	impression	Model cast and laboratory process	conventional	NR	Photography
Kim et al. [45], 2018	ex vivo	No	60/-/-/40/20	1	3D printed tray	bonding on plaster or printed model	model scan	Virtual model, Rapid prototyping	conventional	NR	Scan + Software
			30/-/-/20/10								
		No	60/-/-/40/20		3D printed tray	bonding on plaster or printed model	model scan	Virtual model, Rapid prototyping	conventional	NR	Scan + Software
			30/-/-/20/10								
Schmid et al. [46], 2018	ex vivo	Yes	132/54/24/54/-	1	Silicone	bonding on plaster or printed model	impression	Model cast and laboratory process	conventional	NR	Scan + Software
		Yes	134/52/29/53/-	1	Vacuum form	bonding on plaster or printed model	impression	Model cast and laboratory process	conventional	NR	Scan + Software
Castilla et al. [10], 2014	ex vivo	No	296/98/50/98/50	NR	Double PVS	bonding on plaster or printed model	impression	Model cast and laboratory process	conventional	NR	Photography, digital caliper
			60/20/10/20/10								

		No	296/98/50/98/50		PVS putty	bonding on plaster or printed model	impression	Model cast and laboratory process	conventional	NR	Photography, digital caliper
			60/20/10/20/1								
		No	296/98/50/98/50		PVS-VF	bonding on plaster or printed model	impression	Model cast and laboratory process	conventional	NR	Photography, digital caliper
			60/20/10/20/10								
		No	296/98/50/98/50		Double Vacuum Form	bonding on plaster or printed model	impression	Model cast and laboratory process	conventional	NR	Photography, digital caliper
			58/20/10/18/10								
		No	296/98/50/98/50		Single Vacuum Form	bonding on plaster or printed model	impression	Model cast and laboratory process	conventional	NR	Photography, digital caliper
			58/18/10/20/10								
Koo et al. [18], 1999	ex vivo	No	180/72/26/72/0	9	Silicone	bonding on plaster or printed model	impression	Model cast and laboratory process	conventional	NR	Photography
Chaudhary et al. [47], 2021	in vivo	Yes	300/120/60/120/0	NR	3D printed	bonding on patient	intraoral scan	Virtual model, Rapid prototyping	conventional	NR	Scan + Software
		Yes	300/120/60/120/0		PVS	bonding on patient	intraoral scan	Model cast and laboratory process	conventional	NR	Scan + Software
Xue et al. [26], 2020	in vivo	Yes	205/71/36/62/36	1	3D printed tray	digital or virtual bonding procedure	intraoral scan	Virtual model, Rapid prototyping	conventional	NR	Scan + Software
Grünheid et al. [7], 2016	in vivo	No	136/54/26/46/10	4	Silicone	Bonding on patient	impression	Model cast and laboratory process	conventional	1	CBCT + Software
Hodge et al. [48], 2004	in vivo	Yes	156/104/52/0/0	NR	Vacuum Form	Bonding on patient	impression	Model cast and laboratory process	conventional	NR	Photography, acetate copies

**Table 4:** Summary of the results of the meta-analysis. MTE, mean transfer errors.

Analyzed Parameters		Mesiodistal	Buccolingual	Vertical	Angulation	Rotation	Torque
**Overall accuracy**							
	*n*	23	21	23	20	10	10
	MTE (95% CI)	0.08 (0.05; 0.10)	0.09 (0.06; 0.11)	0.14 (0.10; 0.17)	1.13 (0.75; 1.52)	0.93 (0.49; 1.37)	1.11 (0.68; 1.53)
	Prediction interval	[−0.05; 0.20]	[−0.04; 0.21]	[−0.02; 0.30]	[−0.61; 2.87]	[−0.88; 2.74]	[−0.61; 2.83]
**Tooth group comparison**							
Incisors	*n*	14	12	14	14	8	12
	MTE (95% CI)	0.09 (0.05; 0.12)	0.14 (0.07; 0.21)	0.15 (0.10; 0.20)	1.43 (0.97; 1.89)	0.74 (0.43; 1.05)	1.63 (0.95; 2.32)
	Prediction interval	[−0.04; 0.22]	[−0.11; 0.40]	[−0.09; 0.39]	[−0.32; 3.18]	[−0.18; 1.66]	[−0.81; 4.08]
Canines	*n*	14	12	14	14	8	12
	MTE (95% CI)	0.09 (0.05; 0.13)	0.13 (0.07; 0.19)	0.15 (0.09; 0.24)	1.95 (1.15; 2.75)	0.90 (0.47; 1.32)	2.11 (1.13;3.09)
	Prediction interval	[−0.04; 0.22]	[−0.09; 0.34]	[−0.09; 0.40]	[−1.07; 4.97]	[−0.35; 2.15]	[−1.36; 5.58]
Premolars	*n*	16	14	16	16	16	10
	MTE (95% CI)	0.09 (0.05; 0.13)	0.10 (0.06; 0.14)	0.13 (0.10;0.17)	0.13 (0.10; 0.17)	1.46 (0.97;1.94)	0.95 (0.37; 1.53)
	Prediction interval	[−0.06; 0.24]	[−0.05; 0.24]	[−0.01; 0.27]	[−0.01; 0.27]	[−0.45; 3.36]	[−0.81; 2.71]
Molars	*n*	10	10	10	10	6	10
	MTE (95% CI)	0.06 (0.04; 0.08)	0.09 (−0.04; 0.13)	0.11 (0.04; 0.18)	1.47 (0.70; 2.23)	0.69 (0.32; 1.06)	2.29 (1.20; 3.38)
	Prediction interval	[0.01; 0.11]	[−0.04; 0.21]	[−0.08; 0.31]	[−0.99; 3.92]	[−0.26; 1.64]	[−1.24; 5.82]
**Left vs. Right**							
Left	*n*	5	3	5	2	-	-
	MTE (95% CI)	0.14 (0.04; 0.24)	0.11 (0.06; 0.17)	0.22 (0.10; 0.35)	2.91 (−1.59; 7.41)		
	Prediction interval	[−0.14; 0.42]	[−0.12; 0.35]	[−0.13; 0.57]	-		
Right	*n*	5	3	5	2	-	-
	MTE (95% CI)	0.14 (0.05; 0.22)	0.10 (0.02; 0.17)	0.23 (0.04; 0.42)	2.66 (2.59; 2.72)		
	Prediction interval	[−0.10: 0.37]	[−0.29; 0.48]	[−0.30; 0.76]			
**Upper vs. Lower**							
Upper	*n*	9	7	9	6	4	4
	MTE (95% CI)	0.10 (0.05; 0.16)	0.09 (0.02; 0.15)	0.18 (0.09; 0.26)	1.26 (0.00; 2.53)	0.59 (−0.49; 1.6)	0.73 (−0.50; 1.96)
	Prediction interval	[−0.08; 0.29]	[−0.10; 0.27]	[−0.10; 0.45]	[−2.34; 4.86]	[−2.67; 3.85]	[−2.97; 4.43]
Lower	*n*	4	2	4	4	2	2
	MTE (95% CI)	0.12 (−0.09; 0.33)	0.01 (−0.04; 0.05)	0.22 (−0.00; 0.44)	1.49 (−1.10; 4.08)	0.01 (−0.09;0.10)	0.18 (0.01; 0.35)
	Prediction interval	[−0.52; 0.76]		[−0.10; 0.45]-	[−6.32; 9.31]		
**3D accuracy assessment vs. Photography**							
3D	*n*	18	18	18	18	17	18
	MTE (95% CI)	0.06 (0.04; 0.08)	0.09 (0.05; 0.12)	0.11 (0.09; 0.13)	0.95 (0.63; 1.27)	0.93 (0.49; 1.37)	1.11 (0.68; 1.53)
	Prediction interval	[−0.03; 0.15]	[−0.05; 0.22]	[0.03; 0.18]	[−0.42; 2.32]	[−0.88; 2.74]	[−0.61; 2.83]
Photography	*n*	7	5	7	2	-	-
	MTE (95% CI)	0.12 (0.06; 0.18)	0.09 (0.09; 0.10)	0.22 (0.12; 0.31)	2.74 (−1.50; 6.97)		
	Prediction interval	[−0.05; 0.30]	[ 0.09; 0.10]	[−0.07; 0.50]	-		
**Type of tray**							
3D printed	*n*	13	13	4	13	11	13
	MTE (95% CI)	0.06 (0.03; 0.09)	0.10 (0.06; 0.13)	0.12 (0.09; 0.15)	1.14 (0.69; 1.60)	0.90 (0.36; 1.45)	1.42 (0.76; 2.09)
	Prediction interval	[−0.05; 0.16]	[−0.04; 0.24]	[ 0.02; 0.21]	[−0.57; 2.86]	[−0.94; 2.75]	[−1.01; 3.86]
Silicone	*n*	4	3	4	3	2	2
	MTE (95% CI)	0.10 (0.00; 0.19)	0.08 (−0.01; 0.18)	0.14 (−0.03; 0.32)	1.17 (−1.55; 3.88)	0.66 (−3.82; 5.13)	0.79 (−4.47; 6.05)
	Prediction interval	[−0.20; 0.39]	[−0.44; 0.61]	[−0.38; 0.67]	[−14.17; 17.12]		
Combined Silicone/Vacuum Form	*n*	1	1	1	-	-	-
	MTE (95% CI)	0.09 (0.07; 0.11)	0.09 (0.07; 0.11)	0.14 (0.11; 0.17)			
	Prediction interval	-	-	-			
Vacuum Form	*n*	6	5	6	5	4	4
	MTE (95% CI)	0.10 (0.02; 0.18)	0.08 (−0.03; 0.19)	0.16 (0.03; 0.29)	1.32 (−0.06; 2.71)	1.16 (−0.84; 3.16)	0.86 (0.26;1.46)
	Prediction interval	[−0.13; 0.33]	[−0.22; 0.39]	[−0.20; 0.52]	[−2.52; 5.17]	[−4.80; 7.13]	[−0.92; 2.63]

## Data Availability

Not applicable.

## Supplementary Materials

The following supporting information can be downloaded at: https://www.mdpi.com/article/10.3390/jcm11092568/s1, Supplementary Tables S1–S9. Additional references [65,66,67,68,69,70,71,72,73,74,75,76,77,78,79] are cited in the supplementary content.
