# Supplementary material for: Bracket Transfer Accuracy with the Indirect Bonding Technique—A Systematic Review and Meta-Analysis
Source: J Clin Med. 2022 May 4;11(9):2568. doi: 10.3390/jcm11092568 (PMC9099689; doi:10.3390/jcm11092568)
Supplement: Supplementary file 1 [file jcm-11-02568-s001.zip › jcm-1674713-supplementary.pdf]

# Supplementary content

## Contents

PICO/Eligibility Criteria/Search strategy ........ 3
*Table S1. PICO, eligibility criteria and research question formulation* ........ 3
Excluded studies ........ 4
*Table S2. List of excluded studies with reasons for exclusion.* ........ 4
Risk of bias assessment tool ........ 5
*Table S3. Risk of bias assessment tool* ........ 5
Extracted data from the studies ........ 10
*Table S4.1. Overall mean transfer errors* ........ 10
*Table S4.2. Mean transfer errors in relation to Tooth group* ........ 12
*Table S4.3. Mean transfer errors in relation to side (left vs. right)* ........ 16
*Table S4.4. Mean transfer errors in relation to jaw (upper vs. lower)* ........ 17
*Table S4.5. Mean transfer errors in relation to accuracy assessment method* 18
*Table S4.6. Mean transfer errors in relation to type of tray* ........ 19
Risk of bias assessment ........ 21
*Table S5. Results of risk of bias assessment of individual bracket placement accuracy studies* ........ 21
Meta analytic statistics- Forest Plots ........ 22
*Table S6.1. Linear parameters: Tooth group comparison* ........ 22
*Table S6.2. Linear parameters: Left vs. Right side* ........ 23
*Table S6.3. Linear parameters: Upper vs. Lower jaw* ........ 24
*Table S6.4. Linear parameters: Accuracy assessment method* ........ 25
*Table S6.5. Linear parameters: Type of tray* ........ 26
*Table S6.6. Angular parameters: Tooth group comparison* ........ 27
*Table S6.7. Angular parameters: Left vs. Right side* ........ 28
*Table S6.8. Angular parameters: Upper vs. Lower jaw* ........ 28
*Table S6.9. Angular parameters: Accuracy assessment method* ........ 29
*Table S6.10. Angular parameters: Type of tray* ........ 30
Meta analytic statistics – Funnel Plots ........ 31

*Table S7.1. Linear parameters: Overall accuracy* ............................................ *31*
*Table S7.2. Linear parameters: Tooth group comparison* ................................ *31*
*Table S7.3. Linear parameters: Left vs. Right side* ........................................... *33*
*Table S7.4. Linear parameters: Upper vs. Lower jaw* ...................................... *34*
*Table S7.5. Linear parameters: Accuracy assessment method*......................... *35*
*Table S7.6. Linear parameters: Type of tray*.................................................... *36*
*Table S7.7. Angular parameters: Overall accuracy* .......................................... *37*
*Table S7.8. Angular parameters: Tooth group comparison* ............................. *38*
*Table S7.9. Angular parameters: Left vs. Right side* ........................................ *39*
*Table S7.10. Angular parameters: Upper vs. Lower jaw* .................................. *40*
*Table S7.11. Angular parameters: Accuracy assessment method*.................... *41*
*Table S7.12. Angular parameters: Type of tray*............................................... *42*

Meta analytic statistics – Drapery Plots ............................................................ 44
*Table S8.1. Linear parameters: Overall accuracy* ............................................ *44*
*Table S8.2. Linear parameters: Tooth group comparison* ................................ *44*
*Table S8.3. Linear parameters: Left vs. Right side* ........................................... *46*
*Table S8.4. Linear parameters: Upper vs. Lower jaw* ...................................... *47*
*Table S8.5. Linear parameters: Accuracy assessment method*......................... *48*
*TableS8.6. Linear parameters: Type of tray* ..................................................... *49*
*Table S8.7. Angular parameters: Overall accuracy* .......................................... *50*
*Table S8.8. Angular parameters: Tooth group comparison* ............................. *50*
*Table S8.9. Angular parameters: Left vs. Right side* ........................................ *52*
*Table S8.10. Angular parameters: Upper vs. Lower jaw* .................................. *52*
*Table S8.11. Angular parameters: Accuracy assessment method* .................... *53*
*Table S8.12. Angular parameters: Type of tray* ............................................... *54*

PRISMA 2020 Checklist............................................................................................56
*Table S9. Prisma 2020 checklist* ........................................................................ *56*

References...............................................................................................................61
*Studies excluded after full text reading* ........................................................... *61*
*Studies included in quality assessment and quantitative synthesis*.................. *61*

## PICO/Eligibility Criteria/Search strategy

### Table S1. PICO, eligibility criteria and research question formulation

**PICO model for formulation a research question**

| PICO component | Description | Eligibility criteria |
|---|---|---|
| **P** (problem) | bonding accuracy | Studies investigating bracket transfer accuracy in buccal bracket bonding |
| **I** (Intervention) | indirect bonding | Studies investigating indirect bonding on plaster or printed models (objects) or on patients (subjects) |
| **C** (comparison) | planned bracket position | Actual status of the bracket position was confirmed by comparing to the planned bracket position |
| **O** (outcome) | mean transfer error, accuracy measurement | At least one of the measurements in the linear (mesiodistal, buccolingual, vertical) and/or angular (angulation, rotation, torque) directions was reported |
| **S** (study design) | - | Prospective and retrospective in-vivo and ex-vivo studies |
| **Research question:** To asses the accuracy of indirect bracket bonding by means of the deviations between planned and achieved bracket position in buccal bracket bonding. | | |

## Excluded studies

### Table S2. List of excluded studies with reasons for exclusion.

| Reason for exclusion | Study |
|---|---|
| Out of subject | El Nigoumi et al. (2016); Hiro et al. (2008); Brignardello-Petersen et al. (2020); Mazzeo et al. (2013); Mota Júnior et al. (2018) |
| Missing data | Duarte et al. (2020); Israel et al. (2011); Gayake et al. 2013; Nichols et al. (2013); Nojima et al. (2015) |
| Lingual bonding | Schubert et al. (2013) |
| Reported data not comparable due to applied method | Wendl et al. (2008); Zhang et al. (2020); Mohlhenrich et al. (2020); De Oliveira et al. (2019); Mota Júnior et al. (2015); Oliveira et al. (2019); Shin et al. (2021) |
| Insufficient reporting of the methodology | Aguirre et al. (1982) |

## Risk of bias assessment tool

### Table S3. Risk of bias assessment tool

| Signalling questions | Risk of Bias | Description of the criteria | | |
|---|---|---|---|---|
| **Selection bias** | | | | |
| ***Outset bias*** 1. Was the indirect bracket placement affected by the malocclusion? | Indicators for low RoB | Indirect placement of brackets was not affected by the malocclusion (e.g. severe crowding or rotations). | | |
| | Indicators for high RoB | Indirect placement of brackets was affected by the malocclusion (e.g. severe crowding or rotations). | | |
| | Response options | Yes (Low RoB) | No (High RoB) | Unclear |
| ***Spectrum bias*** 2. Is an appropriate spectrum of teeth selected? | Indicators for low RoB | A relevant group of teeth is assessed (e.g. Incisors, canines, premolars, molars). Number of assessed brackets per group or teeth reported. | | |
| | Indicators for high RoB | Non-tooth related assessment. Number of assessed brackets per group or teeth not reported. | | |
| | Response options | Yes (Low RoB) | No (High RoB) | Unclear |
| ***Sample size*** 3. Is the sample size appropriate? | Indicators for low RoB | The sample size was statistically determined/the sample size is approptiate | | |
| | Indicators for high RoB | No sample size calculation/ the sample size is not approptiate | | |

| | | | | |
|---|---|---|---|---|
| | Response options | Yes (Low RoB) | No (High RoB) | Unclear |
| **Bonding procedure bias** | | | | |
| ***Bonding procedure***<br>*4.* Is procedure correctly performed and described? | Indicators for low RoB | Bonding procedure applied according to the state of the art (justified on basis of references). Same material and method used in all samples of one group. Sufficient description of the bonding procedure. | | |
| | Indicators for high RoB | Modifications of bonding procedure which could bias the assessment. Insufficient description. | | |
| | Response options | Yes (Low RoB) | No (High RoB) | Unclear |
| ***Training bias***<br>5. Were the bonding clinicians trained/calibrated to perform the bonding procedure? | Indicators for low RoB | Reporting of experience. Bonding clinician(s) were experienced/trained for the bonding procedure (e.g. instructions for bonding procedure, predefined workflow for bonding procedure).<br>Ideally full calibration procedure was performed on a separate sample & intra-/interoperator reproducibility values for calibration are given. | | |
| | Indicators for high RoB | Experience not reported or possible bias due to lack of experience. No training performed or insufficiently reported. | | |
| | Response options | Yes (Low RoB) | No (High RoB) | Unclear |
| **Accuracy assessment bias** | | | | |
| ***Accuracy assessment procedure***<br>6. Does the accuracy assessment method correctly measure transfer errors? | Indicators for low RoB | Precise data collection (CBCT, intraoral or model scan) with indication of the system used and its accuracy. No inaccuracys due to further steps within assessment process. Assessment independent from operator (e.g. algorithm-based). | | |
| | Indicators for high RoB | Data collection not precise (impression or photography, caliper), inaccuracy due to further steps in assessment (transfer jigs, tracing, transfer via orthodontic archwires). Assessment dependent on operator (manual measurement). | | |

| | | | | |
|---|---|---|---|---|
| | Response options | Yes (Low RoB) | No (High RoB) | Unclear |
| ***Blinding bias***<br><br>***(Accuracy assessment procedure)***<br><br>7. Is the ***accuracy assessment*** data interpreted independently of the bonding procedure? | Indicators for low RoB | Appropriate blinding of the examiners who are assessing accuracy.<br><br>E.g. randomized/ shuffled allocation of the order of specimen/ images and/or inclusion of multiple examiners, wash-out period between examinations. | | |
| | Indicators for high RoB | Insufficient/no blinding of the examiner/operator. | | |
| | Response options | Yes (Low RoB) | No (High RoB) | Unclear |
| ***Training/Calibration bias***<br><br>***(***Accuracy assessment procedure***)***<br><br>8. Are the examiners/the used computation tool calibrated for performing the accuracy assessment procedure? | Indicators for low RoB | For operator dependent methods (manual measurements/tracings) and non-operator dependent methods (computed accuracy assessment), details and outcomes of training/calibration are given. Calibration training must include an independent sample of individuals or teeth. Calibration data should not interpreted/ misunderstood as intra-examiner reliability. | | |
| | Indicators for high RoB | Calibration not performed or sufficiently reported. | | |
| | Response options | Yes (Low RoB) | No (High RoB) | Unclear |
| **Verification bias** | | | | |
| ***Partial verification bias***<br><br>9. Do all patients/ teeth undergo both the bonding and accuracy assessment procedure? | Indicators for low RoB | Ensure that all patients/teeth/surfaces undergo both procedures (bonding and accuracy assessment). | | |
| | Indicators for high RoB | When a non-random set of patients/teeth/surfaces does not undergo the bonding/accuracy assessment. | | |
| | Categories of RoB | Yes (Low RoB) | No (High RoB) | Unclear |

| | | | | |
|---|---|---|---|---|
| ***Differential verification bias***<br>10. Do all patients/ teeth receive the same accuracy assessment procedure? | Indicators for low RoB | Ensure that all patients/teeth/surfaces received the same accuracy assessment procedure. | | |
| | Indicators for high RoB | When a non-random set of patients/teeth/surfaces is verified with a second or third accuracy assessment procedure. | | |
| | Response options | Yes (Low RoB) | No (High RoB) | Unclear |
| ***Bias in the analysis***<br>11. Are all patient/ teeth, uninterpretable or intermediate test results and withdrawals included in the analysis? | Indicators for low RoB | All patients/teeth who entered the study are accounted for, all uninterpretable or intermediate test results and withdrawals are explained including lost specimen of the brackets/teeth. Numbers of assessed teeth/brackets per group are reported or possible to calculate. | | |
| | Indicators for high RoB | Not all patients/teeth who entered the study are accounted for and not all uninterpretable or intermediate test results and withdrawals are explained including lost specimen of the teeth. Number of assessed teeth/brackets per group are not reported or not possible to calculate. | | |
| | Response options | Yes (Low RoB) | No (High RoB) | Unclear |
| **Outcome bias** | | | | |
| ***Validity bias***<br>12. Are the validation of results for the test method(s) included in the analysis? | Indicators for low RoB | Full presentation of results: data distribution; according to distribution, correct selection and application of the statistical method; indication of all relevant results | | |
| | Indicators for high RoB | Insufficient/ incomplete presentation of results: no indication of data distribution, statistical method insufficiently described or incorrectly selected/applied, relevant results not reported | | |
| | Response options | Yes (Low RoB) | No (High RoB) | Unclear |
| ***Reproducibility bias*** | Indicators for low RoB | Adequate method for reproducibility assessement; correct choice and use of statistical a methods and sufficient reporting of intra-/ inter-examiner reliability, reproducibility (repeated measurements). Data from calibration not missinterpreted as reliability. | | |

| | | | | |
|---|---|---|---|---|
| 13. Are the reliability data of results for the test method(s) included in analysis? | Indicators for high RoB | Insufficient method for reproducibility assessment; incorrect statistical analysis or insufficient reporting. Assessment of intra-/inter-examiner reliability, reproducibility (repeated measurements) not performed or reported. Data from calibration purposes only. Biased interpretation. | | |
| | Response options | Yes (Low RoB) | No (High RoB) | Unclear |

## Extracted data from the studies

### Table S4.1. Overall mean transfer errors

| Author/ Year | Type of study | Type of IDB tray | N | Mesiodistal (mm) | | Buccolingual (mm) | | Vertical (mm) | | Angulation (°) | | Rotation (°) | | Torque (°) | |
|---|---|---|---|---|---|---|---|---|---|---|---|---|---|---|---|
| | | | | Mean | SD | Mean | SD | Mean | SD | Mean | SD | Mean | SD | Mean | SD |
| Jungbauer et al. (2021) | ex vivo | 3D printed | 560 | 0.06 | 0.32 | 0.18 | 0.24 | 0.07 | 0.29 | 2.26 | 1.87 | - | - | 3.07 | 3.01 |
| Faus-Matoses et al. (2021) | ex vivo | 3D printed | 335 | 0.065 | 0.081 | 0.129 | 0.067 | 0.094 | 0.147 | 0.271 | 0.92 | 0.707 | 0.648 | 0.826 | 1.712 |
| Park et al. (2021b) | ex vivo | 3D printed (group A1) | 76 | 0.018 | 0.014 | 0.047 | 0.038 | 0.137 | 0.091 | 0.662 | 0.405 | 0.437 | 0.318 | 0.636 | 0.340 |
| | | 3D printed (group B1) | 76 | 0.043 | 0.056 | 0.043 | 0.028 | 0.127 | 0.066 | 0.644 | 0.435 | 0.433 | 0.230 | 0.542 | 0.255 |
| | | 3D printed (group A2) | 81 | 0.023 | 0.024 | 0.038 | 0.032 | 0.077 | 0.059 | 0.502 | 0.357 | 0.382 | 0.272 | 0.571 | 0.323 |
| | | 3D printed (group B2) | 81 | 0.041 | 0.025 | 0.052 | 0.049 | 0.090 | 0.080 | 0.628 | 0.462 | 0.515 | 0.370 | 0.602 | 0.359 |
| | | 3D printed (group A3) | 68 | 0.019 | 0.018 | 0.036 | 0.038 | 0.094 | 0.097 | 0.549 | 0.337 | 0.377 | 0.328 | 0.592 | 0.217 |
| | | 3D printed (group B3) | 68 | 0.023 | 0.018 | 0.038 | 0.030 | 0.097 | 0.042 | 0.555 | 0.439 | 0.342 | 0.235 | 0.587 | 0.359 |
| Niu et al. (2021) | ex vivo | 3D printed | 108 | 0.07 | 0.06 | 0.13 | 0.15 | 0.19 | 0.2 | 2.25 | 1.97 | 3.14 | 2.91 | 1.22 | 0.9 |
| | | Vacuum Form | 104 | 0.1 | 0.1 | 0.23 | 0.19 | 0.1 | 0.12 | 1.86 | 1.72 | 3.09 | 2.78 | 1.44 | 1.05 |
| Süpple et al. (2021) | ex vivo | Vacuum Form (group H) | 729 | 0.06 | 0.06 | 0.03 | 0.03 | 0.08 | 0.06 | 0.55 | 0.55 | 0.62 | 0.64 | 0.65 | 0.51 |
| | | Vacuum Form (group V) | 724 | 0.06 | 0.06 | 0.02 | 0.04 | 0.08 | 0.07 | 0.56 | 0.55 | 0.59 | 0.61 | 0.67 | 0.57 |
| Pottier et al. (2020) | ex vivo | Silicone | 97 | 0.088 | 0.071 | 0.113 | 0.101 | 0.085 | 0.08 | 0.677 | 0.489 | 1.011 | 0.691 | 1.21 | 0.742 |
| | | 3D printed tray | 98 | 0.198 | 0.11 | 0.2 | 0.111 | 0.197 | 0.233 | 1.422 | 1.067 | 1.336 | 0.782 | 1.566 | 1.1 |
| Kalra et al. (2018) | ex vivo | Vacuum Form | 100 | 0.252 | 0.2 | - | - | 0.395 | 0.337 | 3.099 | 2.477 | - | - | - | - |
| Kim et al. (2018) | ex vivo | 3D printed tray | 30 | 0.09 | 0.09 | 0.08 | 0.05 | 0.14 | 0.14 | 1.53 | 1,57 | 1.1 | 1.19 | 2.65 | 2,91 |
| | | 3D printed tray | 30 | 0.05 | 0.01 | 0.11 | 0.07 | 0.19 | 0.2 | 1.52 | 1.37 | 1.46 | 1.26 | 3.36 | 3.15 |
| Schmid et al. (2018) | ex vivo | Silicone | 132 | 0.029 | 0.022 | 0.045 | 0.031 | 0.071 | 0,052 | 0.414 | 0.882 | 0.306 | 0.234 | 0.382 | 0.292 |
| | | Vacuum form | 134 | 0.046 | 0.039 | 0.048 | 0.035 | 0.105 | 0.078 | 0.647 | 0.755 | 0.438 | 0.42 | 0.703 | 0.913 |
| Grünheid et al. (2016) | ex vivo | Silicone | 136 | 0.07 | 0.117 | 0.001 | 0.131 | 0.025 | 0.160 | 0.159 | 1.574 | 0.197 | 1.374 | 0.120 | 1.757 |

| Author/ Year | Type of study | Type of IDB tray | N | Mesiodistal (mm) | | Buccolingual (mm) | | Vertical (mm) | | Angulation (°) | | Rotation (°) | | Torque (°) | |
|---|---|---|---|---|---|---|---|---|---|---|---|---|---|---|---|
| | | | | **Mean** | **SD** | **Mean** | **SD** | **Mean** | **SD** | **Mean** | **SD** | **Mean** | **SD** | **Mean** | **SD** |
| Castilla et al. (2014)***** | ex vivo | Silicone | 120 | 0.085 | 0.1 | 0.1 | 0.12 | 0.115 | 0.095 | - | - | - | - | - | - |
| | | Vacuum form | 116 | 0.085 | 0.8 | 0.095 | 0.8 | 0.225 | 0.25 | - | - | - | - | - | - |
| | | Silicone + VF | 60 | 0.09 | 0.08 | 0.09 | 0.06 | 0.14 | 0.11 | - | - | - | - | - | - |
| Koo et al. (1999) | ex vivo | Silicone | 180 | 0.18 | 0.14 | - | - | 0.31 | 0.25 | 2.43 | 2.03 | - | - | - | - |
| Xue et al. (2020) | in vivo | 3D printed | 205 | 0.009 | 0.091 | 0.060 | 0.131 | 0.087 | 0.047 | 0.061 | 0.806 | 0.015 | 0.903 | 0.286 | 1.276 |
| Grunheid et al. (2016) | in vivo | Silicone | 136 | 0.007 | 0.117 | 0.001 | 0.131 | 0.025 | 0.160 | 0.159 | 1.574 | 0.197 | 1.374 | 0.120 | 1.757 |
| Hodge et al. (2004) | in vivo | Vacuum Form | 156 | 0.05 | 0.10 | - | - | 0.20 | 0.08 | 0.02 | 0.05 | - | - | - | - |
| Chaudhary et al. (2021) | in vivo | Silicone | 300 | 0.061 | 0.024 | 0.015 | 0.012 | 0.050 | 0.032 | 0.004 | 0.004 | 0.011 | 0.006 | 0.010 | 0.008 |
| | | 3D printed | 300 | 0.010 | 0.022 | 0.005 | 0.003 | 0.026 | 0.010 | 0.001 | 0.002 | 0.003 | 0.005 | 0.007 | 0.003 |

* Data was collected from graphs using a data extraction software

## Table S4.2 Mean transfer errors in relation to Tooth group

| Author/ Year | Type of study | Type of IDB tray | Tooth group | N | Mesiodistal (mm) | | Buccolingual (mm) | | Vertical (mm) | | Angulation (°) | | Rotation (°) | | Torque (°) | |
|---|---|---|---|---|---|---|---|---|---|---|---|---|---|---|---|---|
| | | | | | Mean | SD | Mean | SD | Mean | SD | Mean | SD | Mean | SD | Mean | SD |
| Jungbauer et al. (2021) | ex vivo | 3D printed (hard, crowding) | Canines | 20 | 0.06 | 0.21 | 0.22 | 0.09 | 0.20 | 0.18 | 4.66 | 2.38 | - | - | 3.12 | 1.49 |
| | | 3D printed (hard, no crowding) | Canines | 20 | 0.08 | 0.34 | 0.28 | 0.17 | 0.18 | 0.27 | 4.21 | 1.37 | - | - | 4.23 | 2.09 |
| | | 3D printed (soft, crowding) | Canines | 20 | 0.06 | 0.30 | 0.24 | 0.16 | 0.02 | 0.25 | 2.03 | 1.49 | - | - | 3.34 | 2.94 |
| | | 3D printed (soft, no crowding) | Canines | 20 | 0.00 | 0.31 | 0.27 | 0.21 | 0.1 | 0.25 | 2.38 | 1.44 | - | - | 3.87 | 2.64 |
| Niu et al. (2021) | ex vivo | 3D printed | Canines | 19 | 0.05 | 0.03 | 0.1 | 0.08 | 0.16 | 0.06 | 2.53 | 2.11 | 1.48 | 1.13 | 2.38 | 2.56 |
| | | Vacuum Form | Canines | 18 | 0.12 | 0.08 | 0.07 | 0.06 | 0.15 | 0.07 | 1.85 | 1.09 | 1.65 | 1.16 | 2.6 | 2.4 |
| Süpple et al. (2021) | ex vivo | Vacuum Form | Canines | 107 | 0.07 | 0.06 | 0.03 | 0.05 | 0.07 | 0.07 | 0.72 | 0.65 | 0.82 | 0.8 | 0.64 | 0.55 |
| | | Vacuum Form | Canines | 106 | 0.06 | 0.06 | 0.03 | 0.03 | 0.07 | 0.07 | 0.67 | 0.54 | 0.72 | 0.69 | 0.64 | 0.53 |
| Pottier et al. (2020) | ex vivo | Silicone | Canines | 20 | 0.087 | 0.065 | 0.082 | 0.06 | 0.067 | 0.041 | 0.666 | 0.431 | 0.994 | 0.849 | 1.403 | 0.758 |
| | | 3D printed | Canines | 19 | 0.2 | 0.091 | 0.175 | 0.072 | 0.239 | 0.035 | 1.176 | 0.931 | 1.318 | 0.907 | 1.478 | 0.654 |
| Kalra et al. (2018)* | ex vivo | Vacuum Form | Canines | 10 | 0.255 | 0.171 | - | - | 0.49 | 0.221 | 3.377 | 2.076 | - | - | - | - |
| Schmid et al. (2018) | ex vivo | Silicone | Canines | 24 | 0.024 | 0.016 | 0.04 | 0.036 | 0.076 | 0.066 | 0.225 | 0.199 | 0.237 | 0.166 | 0.456 | 0.298 |
| | | Vacuum form | Canines | 29 | 0.054 | 0.042 | 0.054 | 0.04 | 0.094 | 0.088 | 0.867 | 1.321 | 0.338 | 0.275 | 0.59 | 0.559 |
| Koo et al. (1999)* | ex vivo | Silicone | Canines | 26 | 0.16 | 0.1 | - | - | 0.34 | 0.22 | 2.78 | 1.87 | - | - | - | - |
| Hodge et al. (2004)* | in vivo | Vacuum Form | Canines | 52 | 0.075 | 0.025 | - | - | 0.27 | 0.17 | 0.025 | 0.16 | - | - | - | - |
| Grünheid et al. (2016) | in vivo | Silicone | Canines | 26 | 0.013 | 0.009 | 0.053 | 0.031 | 0.034 | 0.005 | 0.198 | 0.055 | 0.210 | 0 | 0.228 | 0.050 |
| Xue et al. (2020) | in vivo | 3D printed | Canines | 36 | 0.016 | 0.097 | 0.033 | 0.126 | 0.083 | 0.041 | 0.087 | 0.912 | 0.007 | 0.909 | 0.343 | 1.152 |

| Author/ Year | Type of study | Type of IDB tray | Tooth group | N | Mesiodistal (mm) | | Buccolingual (mm) | | Vertical (mm) | | Angulation (°) | | Rotation (°) | | Torque (°) | |
|---|---|---|---|---|---|---|---|---|---|---|---|---|---|---|---|---|
| | | | | | Mean | SD | Mean | SD | Mean | SD | Mean | SD | Mean | SD | Mean | SD |
| Chaudhary et al. (2021)* | in vivo | PVS | Canines | 60 | 0.059 | 0.028 | 0.007 | 0.006 | 0.044 | 0.034 | 0 | 0 | 0 | 0 | 0 | 0 |
| | | 3D printed | | 60 | 0.006 | 0.012 | 0.002 | 0.001 | 0.057 | 0.026 | 0 | 0 | 0 | 0 | 0 | 0 |
| Jungbauer et al. (2021) | ex vivo | 3D printed (hard, crowding) | Incisors | 40 | 0.07 | 0.14 | 0.29 | 0.12 | 0.04 | 0.21 | 2.69 | 1.62 | - | - | 3.12 | 1.49 |
| | | 3D printed (hard, no crowding) | | 40 | 0.10 | 0.30 | 0.33 | 0.15 | 0.20 | 0.34 | 1.96 | 1.05 | - | - | 2.54 | 1.86 |
| | | 3D printed (soft, crowding) | | 40 | 0.10 | 0.22 | 0.27 | 0.18 | 0.07 | 0.28 | 1.62 | 1.49 | - | - | 1.89 | 1.49 |
| | | 3D printed (soft, no crowding) | | 40 | 0.03 | 0.25 | 0.23 | 0.19 | 0.18 | 0.34 | 1.74 | 0.99 | - | - | 2.26 | 1.97 |
| Niu et al. (2021) | ex vivo | 3D printed | Incisors | 37 | 0.06 | 0.04 | 0.08 | 0.06 | 0.19 | 0.11 | 2.2 | 1.13 | 1.28 | 1.03 | 3.14 | 2.77 |
| | | Vacuum Form | | 31 | 0.06 | 0.06 | 0.11 | 0.08 | 0.2 | 0.13 | 1.32 | 0.73 | 1.32 | 0.97 | 2.92 | 2.65 |
| Süpple et al. (2021) | ex vivo | Vacuum Form | Incisors | 210 | 0.05 | 0.04 | 0.02 | 0.02 | 0.07 | 0.05 | 0.77 | 0.61 | 0.53 | 0.47 | 0.49 | 0.36 |
| | | Vacuum Form | | 209 | 0.05 | 0.04 | 0.02 | 0.02 | 0.07 | 0.06 | 0.79 | 0.6 | 0.55 | 0.46 | 0.5 | 0.4 |
| Pottier et al. (2020) | ex vivo | Silicone | Incisors | 38 | 0.082 | 0.065 | 0.134 | 0.12 | 0.072 | 0.054 | 0.459 | 0.335 | 0.652 | 0.538 | 1.011 | 0.711 |
| | | 3D printed | | 40 | 0.181 | 0.095 | 0.179 | 0.104 | 0.151 | 0.102 | 1.245 | 0.724 | 1.013 | 0.709 | 1.41 | 0.659 |
| Kalra et al. (2018)* | ex vivo | Vacuum Form | Incisors | 20 | 0.266 | 0.188 | - | - | 0.47 | 0.279 | 2.479 | 1.697 | - | - | - | - |
| Schmid et al. (2018) | ex vivo | Silicone | Incisors | 54 | 0.021 | 0.016 | 0.04 | 0.021 | 0.063 | 0.04 | 0.345 | 0.474 | 0.299 | 0.258 | 0.331 | 0.291 |
| | | Vacuum form | | 52 | 0.046 | 0.04 | 0.046 | 0.033 | 0.113 | 0.073 | 0.525 | 0.455 | 0.499 | 0.396 | 0.641 | 0.534 |
| Grünheid et al. (2016) | ex vivo | Silicone | Incisors | 54 | 0.12 | 0.009 | 0.054 | 0.041 | 0.026 | 0.013 | 0.061 | 0.005 | 0.235 | 0.010 | 0.103 | 0.085 |
| Koo et al. (1999)* | ex vivo | Silicone | Incisors | 72 | 0.16 | 0.13 | - | - | 0.33 | 0.23 | 2.34 | 1.67 | - | - | - | - |
| Hodge et al. (2004)* | in vivo | Vacuum Form | Incisors | 104 | 0.16 | 0.18 | - | - | 0.04 | 0.20 | 0.02 | 0.11 | - | - | - | - |
| Grünheid et al. (2016) | in vivo | Silicone | Incisors | 54 | 0.12 | 0.009 | 0.054 | 0.041 | 0.026 | 0.013 | 0.061 | 0.005 | 0.235 | 0.010 | 0.103 | 0.085 |
| Xue et al. (2020) | in vivo | 3D printed | Incisors | 71 | 0.013 | 0.079 | 0.123 | 0.064 | 0.088 | 0.041 | 0.061 | 0.660 | 0.014 | 0.821 | 0.371 | 0.742 |

| Author/ Year | Type of study | Type of IDB tray | Tooth group | N | Mesiodistal (mm) | | Buccolingual (mm) | | Vertical (mm) | | Angulation (°) | | Rotation (°) | | Torque (°) | |
|---|---|---|---|---|---|---|---|---|---|---|---|---|---|---|---|---|
| | | | | | Mean | SD | Mean | SD | Mean | SD | Mean | SD | Mean | SD | Mean | SD |
| Chaudhary et al. (2021)* | in vivo | PVS | Incisors | 120 | 0.059 | 0.020 | 0.036 | 0.028 | 0.053 | 0.030 | 0.010 | 0.013 | 0.025 | 0.008 | 0.010 | 0.014 |
| | | 3D printed | | 120 | 0.019 | 0.043 | 0.012 | 0.009 | 0.060 | 0.029 | 0.003 | 0.005 | 0.002 | 0.006 | 0.001 | 0.001 |
| Jungbauer et al. (2021) | ex vivo | 3D printed (hard, crowding) | Molars | 40 | 0.04 | 0.29 | 0.04 | 0.25 | 0.01 | 0.21 | 1.96 | 1.70 | - | - | 3.08 | 2.05 |
| | | 3D printed (hard, no crowding) | | 40 | 0.10 | 0.37 | 0.15 | 0.17 | 0.02 | 0.27 | 2.41 | 1.81 | - | - | 3.73 | 2.68 |
| | | 3D printed (soft, crowding) | | 40 | 0.00 | 0.42 | 0.06 | 0.28 | 0.13 | 0.35 | 2.41 | 2.73 | - | - | 3.97 | 3.73 |
| | | 3D printed (soft, no crowding) | | 40 | 0.01 | 0.44 | 0.09 | 0.22 | 0.03 | 0.29 | 2.15 | 1.85 | - | - | 2.89 | 2.02 |
| Park et al. (2021a) | ex vivo | 3D printed (A) | Molars | 158 | 0,03 | 0,027 | 0,057 | 0,062 | 0,131 | 0,084 | 0,671 | 0,545 | 0,435 | 0,3 | 0,679 | 0,358 |
| | | 3D printed (B) | | 158 | 0,065 | 0,08 | 0,097 | 0,054 | 0,111 | 0,103 | 0,633 | 0,531 | 0,447 | 0,344 | 0,726 | 0,436 |
| Niu et al. (2021) | ex vivo | 3D printed | Molars | 20 | 0.1 | 0.08 | 0.25 | 0.28 | 0.29 | 0.39 | 1.29 | 0.8 | 1.18 | 0.71 | 4.4 | 4.45 |
| | | Vacuum Form | | 20 | 0.13 | 0.16 | 0.18 | 0.22 | 0.33 | 0.24 | 3.52 | 2.81 | 1.29 | 1.18 | 3.32 | 4.03 |
| Süpple et al. (2021) | ex vivo | Vacuum Form | Molars | 205 | 0.06 | 0.07 | 0.03 | 0.03 | 0.09 | 0.06 | 0.23 | 0.26 | 0.56 | 0.62 | 0.68 | 0.49 |
| | | Vacuum Form | | 203 | 0.06 | 0.07 | 0.03 | 0.03 | 0.1 | 0.08 | 0.26 | 0.37 | 0.56 | 0.64 | 0.79 | 0.68 |
| Grünheid et al. (2016) | ex vivo | Silicone | Molars | 10 | 0.063 | 0.033 | 0.045 | 0.074 | 0.035 | 0.017 | 0.213 | 0.445 | 0.405 | 0.335 | 0.268 | 0.520 |
| Xue et al. (2020) | in vivo | 3D printed | Molars | 36 | 0.007 | 0.094 | 0.125 | 0.087 | 0.091 | 0.054 | 0.060 | 0.900 | 0.047 | 0.906 | 0.690 | 1.473 |
| Niu et al. (2021) | ex vivo | 3D printed | Premolars | 32 | 0.07 | 0.08 | 0.16 | 0.13 | 0.13 | 0.19 | 2.68 | 2.18 | 0.98 | 0.66 | 2.77 | 1.72 |
| | | Vacuum Form | | 35 | 0.11 | 0.1 | 0.07 | 0.03 | 0.23 | 0.18 | 1.36 | 0.36 | 3.32 | 3.05 | 1.52 | 1.06 |
| Süpple et al. (2021) | ex vivo | Vacuum Form | Premolars | 207 | 0.07 | 0.07 | 0.02 | 0.02 | 0.09 | 0.07 | 0.55 | 0.48 | 0.67 | 0.69 | 0.8 | 0.59 |
| | | Vacuum Form | | 206 | 0.06 | 0.07 | 0.02 | 0.05 | 0.08 | 0.06 | 0.56 | 0.54 | 0.59 | 0.67 | 0.74 | 0.58 |
| Pottier et al. (2020) | ex vivo | Silicone | Premolars | 39 | 0.095 | 0.74 | 0.109 | 0.083 | 0.108 | 0.113 | 0.895 | 0.56 | 1.371 | 0.545 | 1.305 | 0.722 |
| | | 3D printed | | 39 | 0.215 | 0.134 | 0.234 | 0.137 | 0.225 | 0.249 | 1.724 | 1.335 | 1.677 | 0.653 | 1.769 | 1.53 |
| Kalra et al. (2018)* | ex vivo | Vacuum Form | Premolars | 20 | 0.238 | 0.16 | - | - | 0.266 | 0.202 | 3.247 | 2.239 | - | - | - | - |

| Author/ Year | Type of study | Type of IDB tray | Tooth group | N | Mesiodistal (mm) | | Buccolingual (mm) | | Vertical (mm) | | Angulation (°) | | Rotation (°) | | Torque (°) | |
|---|---|---|---|---|---|---|---|---|---|---|---|---|---|---|---|---|
| | | | | | Mean | SD | Mean | SD | Mean | SD | Mean | SD | Mean | SD | Mean | SD |
| Schmid et al. (2018) | ex vivo | Silicone | Premolars | 54 | 0.04 | 0.026 | 0.052 | 0.037 | 0.077 | 0.056 | 0.568 | 1.28 | 0.344 | 0.23 | 0.401 | 0.286 |
| | | Vacuum form | | 53 | 0.043 | 0.036 | 0.046 | 0.034 | 0.102 | 0.077 | 0.647 | 0.522 | 0.431 | 0.496 | 0.825 | 1.288 |
| Koo et al. (1999)* | ex vivo | Silicone | Premolars | 72 | 0.21 | 0.14 | - | - | 0.27 | 0.18 | 2.74 | 2.23 | - | - | - | - |
| Grünheid et al. (2016) | in vivo | Silicone | Premolars | 46 | 0.380 | 0.003 | 0.492 | 0.062 | 0.216 | 0.053 | 0.047 | 0.145 | 0.040 | 0.010 | 0.401 | 0.415 |
| Xue et al. (2020) | in vivo | 3D printed | Premolars | 62 | 0.001 | 0.099 | 0.031 | 0.152 | 0.089 | 0.049 | 0.078 | 0.823 | 0.064 | 0.986 | 0.794 | 1.359 |
| Chaudhary et al. (2021)* | in vivo | PVS | Premolars | 120 | 0.066 | 0.025 | 0.001 | 0.003 | 0.054 | 0.032 | 0 | 0 | 0.009 | 0.012 | 0.022 | 0.009 |
| | | 3D printed | | 120 | 0.005 | 0.012 | 0.003 | 0 | 0.070 | 0.025 | 0 | 0 | 0.008 | 0.009 | 0.020 | 0.007 |
| Park et al. (2021a) | ex vivo | 3D printed (A) | Premolars | 122 | 0,022 | 0,026 | 0,053 | 0,07 | 0,1 | 0,055 | 0,545 | 0,442 | 0,467 | 0,396 | 0,551 | 0,299 |
| | | 3D printed (B) | | 122 | 0,034 | 0,033 | 0,06 | 0,071 | 0,092 | 0,085 | 0,639 | 0,488 | 0,459 | 0,342 | 0,598 | 0,414 |

*Data was calculated on the basis of published data for individual teeth

## Table S4.3. Mean transfer errors in relation to side (left vs. right)

| Author/ Year | Type of study | Type of IDB tray | Left/Right | N | Mesiodistal (mm) | | Buccolingual (mm) | | Vertical (mm) | | Angulation (°) | | Rotation (°) | | Torque (°) | |
|---|---|---|---|---|---|---|---|---|---|---|---|---|---|---|---|---|
| | | | | | Mean | SD | Mean | SD | Mean | SD | Mean | SD | Mean | SD | Mean | SD |
| Kalra et al. (2018)* | ex vivo | Vacuum Form | left | 100 | 0.2743 | 0.2 | - | - | 0.34 | 0.22 | 3.28 | 2.11 | - | - | - | - |
| Castilla et al. (2014) | ex vivo | Silicone | left | 60 | 0.085 | 0.093 | 0.094 | 0.141 | 0.135 | 0.109 | - | - | - | - | - | - |
| Castilla et al. (2014) | ex vivo | Vacuum Form | left | 60 | 0.089 | 0.095 | 0.110 | 0.089 | 0.256 | 0.267 | - | - | - | - | - | - |
| Castilla et al. (2014) | ex vivo | Silicone + VF | left | 30 | 0.090 | 0.069 | 0.141 | 0.098 | 0.101 | 0.069 | - | - | - | - | - | - |
| Koo et al. (1999)* | ex vivo | Silicone | left | 180 | 0.163 | 0.124 | - | - | 0.286 | 0.168 | 2,571 | 1,958 | - | - | - | - |
| Kalra et al. (2018)* | ex vivo | Vacuum Form | right | 100 | 0.2305 | 0.15 | - | - | 0.44 | 0.25 | 2.65 | 1.87 | - | - | - | - |
| Castilla et al. (2014) | ex vivo | Silicone | right | 56 | 0.081 | 0.103 | 0.095 | 0.07 | 0.091 | 0.074 | - | - | - | - | - | - |
| Castilla et al. (2014) | ex vivo | Vacuum Form | right | 56 | 0.081 | 0.060 | 0.079 | 0.069 | 0.20 | 0.194 | - | - | - | - | - | - |
| Castilla et al. (2014) | ex vivo | Silicone + VF | right | 30 | 0.103 | 0.074 | 0.143 | 0.127 | 0.090 | 0.058 | - | - | - | - | - | - |
| Koo et al. (1999)* | ex vivo | Silicone | right | 180 | 0.191 | 0.131 | - | - | 0.333 | 0.243 | 2,661 | 1,909 | - | - | - | - |

*Data was calculated on the basis of published data for individual teeth

## Table S4.4. Mean transfer errors in relation to jaw (upper vs. lower)

| Author/ Year | Type of study | Type of IDB tray | Upper/ Lower | N | Mesiodistal (mm) | | Buccolingual (mm) | | Vertical (mm) | | Angulation (°) | | Rotation (°) | | Torque (°) | |
|---|---|---|---|---|---|---|---|---|---|---|---|---|---|---|---|---|
| | | | | | Mean | SD | Mean | SD | Mean | SD | Mean | SD | Mean | SD | Mean | SD |
| Kalra et al. (2018)* | ex vivo | Vacuum Form | lower | 100 | 0.28 | 0.19 | - | - | 0.3849 | 0.213 | 3.07 | 1.89 | - | - | - | - |
| Koo et al. (1999)* | ex vivo | Silicone | lower | 180 | 0.18 | 0.13 | - | - | 0.28 | 0.17 | 2.74 | 1.78 | - | - | - | - |
| Park et al. (2021b) | ex vivo | 3D printed (A) | lower | 109 | 0.005 | 0.048 | 0.009 | 0.052 | 0.097 | 0.096 | 0.095 | 0.649 | 0.001 | 0.540 | 0.168 | 0.651 |
| | | 3D printed (B) | | 109 | 0.012 | 0.038 | 0.002 | 0.064 | 0.119 | 0.087 | 0.091 | 0.781 | 0.016 | 0.598 | 0.195 | 0.677 |
| Pottier et al. (2020) | ex vivo | Silicone | upper | 97 | 0.088 | 0.071 | 0.113 | 0.101 | 0.085 | 0.08 | 0.677 | 0.489 | 1,011 | 0.691 | 1,210 | 0.742 |
| | | 3D printed tray | | 98 | 0.198 | 0.110 | 0.2 | 0.111 | 0.197 | 0.233 | 1,422 | 1,067 | 1,336 | 0.782 | 1,566 | 1.1 |
| Kalra et al. (2018)* | ex vivo | Vacuum Form | upper | 100 | 0.21 | 0.16 | - | - | 0.40 | 0.2605 | 2.86 | 2.09 | - | - | - | - |
| Castilla et al. (2014) | ex vivo | Silicone | upper | 120 | 0.085 | 0.1 | 0.1 | 0.12 | 0.115 | 0.095 | - | - | - | - | - | - |
| | | Vacuum Form | | 116 | 0.085 | 0.8 | 0.095 | 0.8 | 0.225 | 0.25 | - | - | - | - | - | - |
| | | Silicone + VF | | 60 | 0.09 | 0.08 | 0.09 | 0.06 | 0.14 | 0.11 | - | - | - | - | - | - |
| Koo et al. (1999)* | ex vivo | Silicone | upper | 180 | 0.16 | 0.12 | - | - | 0.29 | 0.17 | 2.52 | 1.96 | - | - | - | - |
| Park et al. (2021b) | ex vivo | 3D printed (A) | upper | 116 | 0.004 | 0.048 | 0.010 | 0.051 | 0.071 | 0.095 | 0.139 | 0.691 | 0.029 | 0.458 | 0.151 | 0.680 |
| | | 3D printed (B) | | 116 | 0.012 | 0.029 | 0.008 | 0.052 | 0.075 | 0.056 | 0.014 | 0.754 | 0.007 | 0.467 | 0.004 | 0.627 |

* Data was calculated on the basis of published data for individual teeth

## Table S4.5. Mean transfer errors in relation to accuracy assessment method

| Author/ Year | Type of study | Accuracy assessment method | N | Mesiodistal (mm) | | Buccolingual (mm) | | Vertical (mm) | | Angulation (°) | | Rotation (°) | | Torque (°) | |
|---|---|---|---|---|---|---|---|---|---|---|---|---|---|---|---|
| | | | | Mean | SD | Mean | SD | Mean | SD | Mean | SD | Mean | SD | Mean | SD |
| Jungbauer et al. (2021) | ex vivo | 3D | 560 | 0.06 | 0.32 | 0.18 | 0.24 | 0.07 | 0.29 | 2.26 | 1.87 | - | - | 3.07 | 3.01 |
| Faus-Matoses et al. (2021) | ex vivo | 3D | 335 | 0.065 | 0.081 | 0.129 | 0.067 | 0.094 | 0.147 | 0.271 | 0.92 | 0.707 | 0.648 | 0.826 | 1.712 |
| Park et al. (2021b) | ex vivo | 3D | 76 | 0.018 | 0.014 | 0.047 | 0.038 | 0.137 | 0.091 | 0.662 | 0.405 | 0.437 | 0.318 | 0.636 | 0.340 |
| | | | 76 | 0.043 | 0.056 | 0.043 | 0.028 | 0.127 | 0.066 | 0.644 | 0.435 | 0.433 | 0.230 | 0.542 | 0.255 |
| | | | 81 | 0.023 | 0.024 | 0.038 | 0.032 | 0.077 | 0.059 | 0.502 | 0.357 | 0.382 | 0.272 | 0.571 | 0.323 |
| | | | 81 | 0.041 | 0.025 | 0.052 | 0.049 | 0.090 | 0.080 | 0.628 | 0.462 | 0.515 | 0.370 | 0.602 | 0.359 |
| | | | 68 | 0.019 | 0.018 | 0.036 | 0.038 | 0.094 | 0.097 | 0.549 | 0.337 | 0.377 | 0.328 | 0.592 | 0.217 |
| | | | 68 | 0.023 | 0.018 | 0.038 | 0.030 | 0.097 | 0.042 | 0.555 | 0.439 | 0.342 | 0.235 | 0.587 | 0.359 |
| Niu et al. (2021) | ex vivo | 3D | 108 | 0.07 | 0.06 | 0.13 | 0.15 | 0.19 | 0.2 | 2.25 | 1.97 | 3.14 | 2.91 | 1.22 | 0.9 |
| | | | 104 | 0.1 | 0.1 | 0.23 | 0.19 | 0.1 | 0.12 | 1.86 | 1.72 | 3.09 | 2.78 | 1.44 | 1.05 |
| Süpple et al. (2021) | ex vivo | 3D | 729 | 0.06 | 0.06 | 0.03 | 0.03 | 0.08 | 0.06 | 0.55 | 0.55 | 0.62 | 0.64 | 0.65 | 0.51 |
| | | | 724 | 0.06 | 0.06 | 0.02 | 0.04 | 0.08 | 0.07 | 0.56 | 0.55 | 0.59 | 0.61 | 0.67 | 0.57 |
| Pottier et al. (2020) | ex vivo | 3D | 97 | 0.088 | 0.071 | 0.113 | 0.101 | 0.085 | 0.08 | 0.677 | 0.489 | 1.011 | 0.691 | 1.21 | 0.742 |
| | | | 98 | 0.198 | 0.11 | 0.2 | 0.111 | 0.197 | 0.233 | 1.422 | 1.067 | 1.336 | 0.782 | 1.566 | 1.1 |
| Kim et al. (2018) | ex vivo | 3D | 30 | 0.09 | 0.09 | 0.08 | 0.05 | 0.14 | 0.14 | 1.53 | 1,57 | 1.1 | 1.19 | 2.65 | 2,91 |
| | | | 30 | 0.05 | 0.01 | 0.11 | 0.07 | 0.19 | 0.2 | 1.52 | 1.37 | 1.46 | 1.26 | 3.36 | 3.15 |
| Schmid et al. (2018) | ex vivo | 3D | 132 | 0.029 | 0.022 | 0.045 | 0.031 | 0.071 | 0,052 | 0.414 | 0.882 | 0.306 | 0.234 | 0.382 | 0.292 |
| | | | 134 | 0.046 | 0.039 | 0.048 | 0.035 | 0.105 | 0.078 | 0.647 | 0.755 | 0.438 | 0.42 | 0.703 | 0.913 |
| Grünheid et al. (2016) | in vivo | 3D | 136 | 0.07 | 0.117 | 0.001 | 0.131 | 0.025 | 0.160 | 0.159 | 1.574 | 0.197 | 1.374 | 0.120 | 1.757 |
| Xue et al. (2020) | in vivo | 3D | 205 | 0.009 | 0.091 | 0.060 | 0.131 | 0.087 | 0.047 | 0.061 | 0.806 | 0.015 | 0.903 | 0.286 | 1.276 |
| Chaudhary et al. (2021) | in vivo | 3D | 300 | 0.010 | 0.022 | 0.005 | 0.003 | 0.026 | 0.010 | 0.001 | 0.002 | 0.003 | 0.005 | 0.007 | 0.003 |
| | | | 300 | 0.061 | 0.024 | 0.015 | 0.012 | 0.050 | 0.032 | 0.004 | 0.004 | 0.011 | 0.006 | 0.010 | 0.008 |
| Kalra et al. (2018) | ex vivo | Photography | 100 | 0.252 | 0.2 | - | - | 0.395 | 0.337 | 3.099 | 2.477 | - | - | - | - |

| Author/ Year | Type of study | Accuracy assessment method | N | Mesiodistal (mm) | | Buccolingual (mm) | | Vertical (mm) | | Angulation (°) | | Rotation (°) | | Torque (°) | |
|---|---|---|---|---|---|---|---|---|---|---|---|---|---|---|---|
| | | | | Mean | SD | Mean | SD | Mean | SD | Mean | SD | Mean | SD | Mean | SD |
| Castilla et al. (2014) | ex vivo | Photography | 60 | 0.09 | 0.14 | 0.1 | 0.07 | 0.11 | 0.1 | - | - | - | - | - | - |
| | | | 60 | 0.08 | 0.06 | 0.1 | 0.17 | 0.12 | 0.09 | - | - | - | - | - | - |
| | | | 60 | 0.09 | 0.08 | 0.09 | 0.06 | 0.14 | 0.11 | - | - | - | - | - | - |
| | | | 58 | 0.08 | 0.07 | 0.09 | 0.07 | 0.21 | 0.22 | - | - | - | - | - | - |
| | | | 58 | 0.09 | 0.09 | 0.1 | 0.09 | 0.24 | 0.28 | - | - | - | - | - | - |
| Koo et al. (1999) | ex vivo | Photography | 180 | 0.18 | 0.14 | - | - | 0.31 | 0.25 | 2.43 | 2.03 | - | - | - | - |

## Table S4.6. Mean transfer errors in relation to type of tray

| Author/ Year | Type of study | Type of tray | N | Mesiodistal (mm) | | Buccolingual (mm) | | Vertical (mm) | | Angulation (°) | | Rotation (°) | | Torque (°) | |
|---|---|---|---|---|---|---|---|---|---|---|---|---|---|---|---|
| | | | | Mean | SD | Mean | SD | Mean | SD | Mean | SD | Mean | SD | Mean | SD |
| Jungbauer et al. (2021) | ex vivo | 3D printed (hard) | 280 | 0,08 | 0,3 | 0,2 | 0,22 | 0,1 | 0,27 | 2,55 | 1,85 | - | - | 3,27 | 2,36 |
| | | 3D printed (soft) | 280 | 0,04 | 0,35 | 0,17 | 0,25 | 0,03 | 0,3 | 1,98 | 1,85 | - | - | 2,86 | 3,54 |
| Faus-Matoses et al. (2021) | ex vivo | 3D printed | 335 | 0.065 | 0.081 | 0.129 | 0.067 | 0.094 | 0.147 | 0.271 | 0.92 | 0.707 | 0.648 | 0.826 | 1.712 |
| Park et al. (2021b) | ex vivo | 3D printed (group A1) | 76 | 0.018 | 0.014 | 0.047 | 0.038 | 0.137 | 0.091 | 0.662 | 0.405 | 0.437 | 0.318 | 0.636 | 0.340 |
| | | 3D printed (group B1) | 76 | 0.043 | 0.056 | 0.043 | 0.028 | 0.127 | 0.066 | 0.644 | 0.435 | 0.433 | 0.230 | 0.542 | 0.255 |
| | | 3D printed (group A2) | 81 | 0.023 | 0.024 | 0.038 | 0.032 | 0.077 | 0.059 | 0.502 | 0.357 | 0.382 | 0.272 | 0.571 | 0.323 |
| | | 3D printed (group B2) | 81 | 0.041 | 0.025 | 0.052 | 0.049 | 0.090 | 0.080 | 0.628 | 0.462 | 0.515 | 0.370 | 0.602 | 0.359 |
| | | 3D printed (group A3) | 68 | 0.019 | 0.018 | 0.036 | 0.038 | 0.094 | 0.097 | 0.549 | 0.337 | 0.377 | 0.328 | 0.592 | 0.217 |
| | | 3D printed (group B3) | 68 | 0.023 | 0.018 | 0.038 | 0.030 | 0.097 | 0.042 | 0.555 | 0.439 | 0.342 | 0.235 | 0.587 | 0.359 |
| Niu et al. (2021) | ex vivo | 3D printed | 108 | 0.07 | 0.06 | 0.13 | 0.15 | 0.19 | 0.2 | 2.25 | 1.97 | 3.14 | 2.91 | 1.22 | 0.9 |

| Author/ Year | Type of study | Type of tray | N | Mesiodistal (mm) | | Buccolingual (mm) | | Vertical (mm) | | Angulation (°) | | Rotation (°) | | Torque (°) | |
|---|---|---|---|---|---|---|---|---|---|---|---|---|---|---|---|
| | | | | Mean | SD | Mean | SD | Mean | SD | Mean | SD | Mean | SD | Mean | SD |
| Pottier et al. (2020) | ex vivo | 3D printed | 98 | 0.198 | 0.110 | 0.200 | 0.111 | 0.197 | 0.233 | 1.422 | 1.067 | 1.336 | 0.782 | 1.566 | 1.100 |
| Kim et al. (2018) * | ex vivo | 3D printed | 30 | 0.09 | 0.09 | 0.08 | 0.05 | 0.14 | 0.14 | 1.53 | 1.57 | 1.1 | 1.19 | 2.65 | 2.91 |
| | | 3D printed | 30 | 0.05 | 0.01 | 0.11 | 0.07 | 0.19 | 0.2 | 1.52 | 1.37 | 1.46 | 1.26 | 3.36 | 3.15 |
| Xue et al. (2020) | in vivo | 3D printed | 205 | 0.009 | 0.091 | 0.060 | 0.131 | 0.087 | 0.047 | 0.061 | 0.806 | 0.015 | 0.903 | 0.286 | 1.276 |
| Chaudhary et al. (2021)* | in vivo | 3D printed | 300 | 0.010 | 0.022 | 0.005 | 0.003 | 0.026 | 0.010 | 0.001 | 0.002 | 0.003 | 0.005 | 0.007 | 0.003 |
| Pottier et al. (2020) | ex vivo | Silicone | 97 | 0.088 | 0.071 | 0.113 | 0.101 | 0.085 | 0.08 | 0.677 | 0.489 | 1.011 | 0.691 | 1.210 | 0.742 |
| Schmid et al. (2018) | ex vivo | Silicone | 132 | 0.029 | 0.022 | 0.045 | 0.031 | 0.071 | 0.052 | 0.414 | 0.882 | 0.306 | 0.234 | 0.382 | 0.292 |
| Grünheid et al. (2016) | in vivo | Silicone | 136 | 0.07 | 0.117 | 0.001 | 0.131 | 0.025 | 0.160 | 0.159 | 1.574 | 0.197 | 1.374 | 0.120 | 1.757 |
| Castilla et al. (2014) | ex vivo | Silicone | 120 | 0.085 | 0.1 | 0.1 | 0.12 | 0.115 | 0.095 | - | - | - | - | - | - |
| Koo et al. (1999)* | ex vivo | Silicone | 180 | 0.18 | 0.14 | - | - | 0.31 | 0.25 | 2.43 | 2.03 | - | - | - | - |
| Chaudhary et al. (2021)* | in vivo | Silicone | 300 | 0.061 | 0.024 | 0.015 | 0.012 | 0.050 | 0.032 | 0.004 | 0.004 | 0.011 | 0.006 | 0.010 | 0.008 |
| Castilla et al. (2014) | ex vivo | Silicone + VF | 60 | 0.09 | 0.08 | 0.09 | 0.06 | 0.14 | 0.11 | - | - | - | - | - | - |
| Niu et al. (2021) | ex vivo | Vacuum Form | 104 | 0.1 | 0.1 | 0.23 | 0.19 | 0.1 | 0.12 | 1.86 | 1.72 | 3.09 | 2.78 | 1.44 | 1.05 |
| Süpple et al. (2021) | ex vivo | Vacuum Form | 729 | 0.06 | 0.06 | 0.03 | 0.03 | 0.08 | 0.06 | 0.55 | 0.55 | 0.62 | 0.64 | 0.65 | 0.51 |
| | | Vacuum Form | 724 | 0.06 | 0.06 | 0.02 | 0.04 | 0.08 | 0.07 | 0.56 | 0.55 | 0.59 | 0.61 | 0.67 | 0.57 |
| Kalra et al. (2018)* | ex vivo | Vacuum Form | 100 | 0.252 | 0.2 | - | - | 0.395 | 0.337 | 3.099 | 2.477 | - | - | - | - |
| Schmid et al. (2018) | ex vivo | Vacuum Form | 134 | 0.046 | 0.039 | 0.048 | 0.035 | 0.105 | 0.078 | 0.647 | 0.755 | 0.438 | 0.420 | 0.703 | 0.913 |
| Castilla et al. (2014) | ex vivo | Vacuum Form | 116 | 0.085 | 0.8 | 0.095 | 0.8 | 0.225 | 0.25 | - | - | - | - | - | - |
| Hodge et al. (2004)* | in vivo | Vacuum Form | 156 | 0.05 | 0.10 | - | - | 0.20 | 0.08 | 0.02 | 0.05 | - | - | - | - |

* Data was calculated on the basis of published data for individual teeth

## Risk of bias assessment

### Table S5. Results of risk of bias assessment of individual bracket placement accuracy studies

| Visual examination of *in vitro* validation studies on proximal surfaces | Signaling questions | | | | | | | | | | | | |
|---|---|---|---|---|---|---|---|---|---|---|---|---|---|
| | Selection bias | | | Bonding procedure bias | | Accuracy assessment bias | | | Verification bias | | | Outcome bias | |
| | Outset bias | Spectrum bias | Sample size | Bonding procedure | Training bias | Accuracy assess. procedure | Blinding bias | Calibration bias | Partial ver. bias | Differential ver. bias | Bias in the Analysis | Validity bias | Reproducibility bias |
| Jungbauer et al. Appl Sci 2021 | - | + | + | + | + | + | - | - | + | + | + | + | + |
| Faus-Matoses et al. JPM 2021 | + | + | - | + | + | + | - | - | + | + | ? | + | - |
| Park et al. KJO 2021 | - | ? | + | + | - | + | - | - | + | + | + | + | + |
| Park et al. Sensors 2021 | - | + | - | + | - | + | - | - | + | + | + | + | + |
| Castilla et al. Angle Orthod 2014 | + | + | - | + | - | ? | - | - | + | + | - | ? | - |
| Grünheid et al. Angle Orthod 2016 | ? | + | - | + | - | + | ? | - | + | + | + | + | ? |
| Hodge et al. J Orthod 2004 | + | - | + | + | - | - | + | - | + | + | + | + | ? |
| Kalra et al. J Clin Diagn Res 2018 | + | + | - | + | + | ? | - | - | + | + | - | + | + |
| Kim et al. Am J Orthod Dentofacial Orthop 2018 | - | - | - | + | - | + | - | - | + | + | + | ? | + |
| Koo et al. Am J Orthod Dentofacial Orthop 1999 | ? | + | - | + | + | ? | - | - | + | + | + | + | - |
| Niu et al. Angle Ortho. 2021 | + | + | - | + | - | + | ? | - | + | + | + | + | + |
| Pottier et al. Clin Oral Investig 2020 | + | + | + | + | ? | + | - | - | + | + | + | + | + |
| Schmid et al. Eur J Orthod 2018 | ? | + | + | + | ? | + | - | - | + | + | + | + | - |
| Süpple et al. J Clin Med 2021 | + | + | - | + | ? | + | - | - | + | + | + | + | - |
| Xue et al. Am J Orthod Dentofacial Orthop 2020 | + | + | + | + | - | + | - | - | + | + | + | + | + |
| Chaudhary et. al J Orthod 2021 | ? | + | + | + | - | + | + | - | + | + | + | - | ? |

Legend: +=Low risk of bias (Yes); -=High risk of bias (Probably No, No); ?=Unclear (No information, Incomplete reporting, Probably Yes) x=Question for in vivo studies

## Meta analytic statistics- Forest Plots

### Table S6.1. Linear parameters: Tooth group comparison

| Analysed linear parameters | Mesiodistal | Buccolingual | Vertical |
|---|---|---|---|
| Incisors | 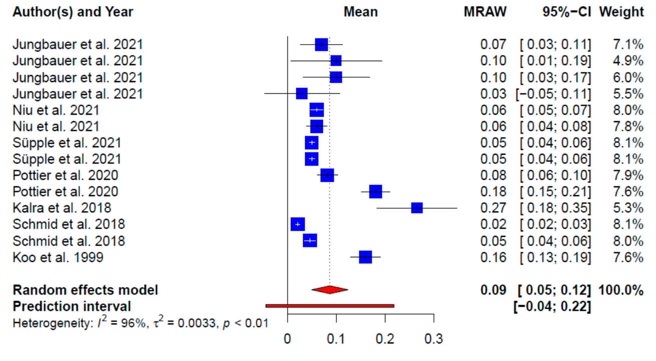 | 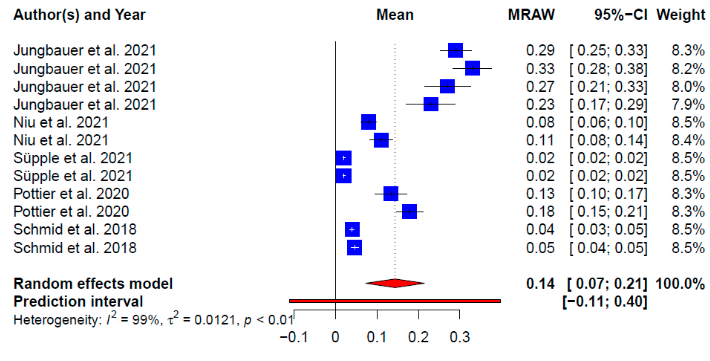 | 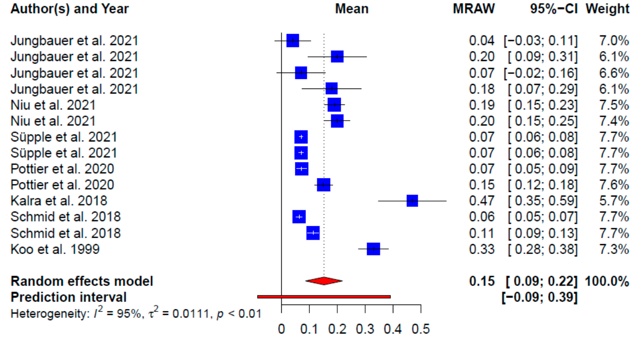 |
| Canines | 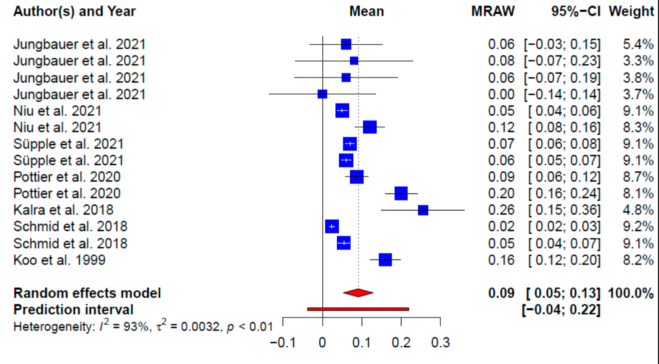 | 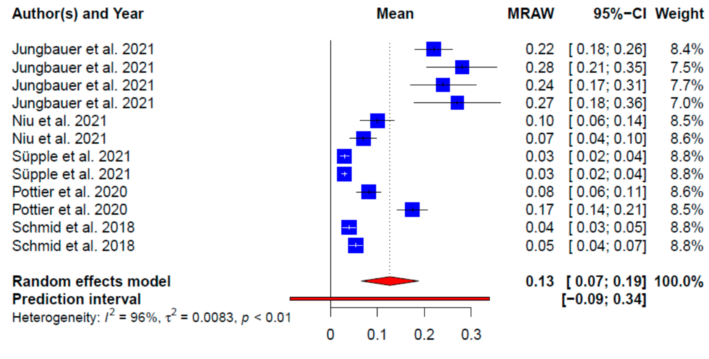 | 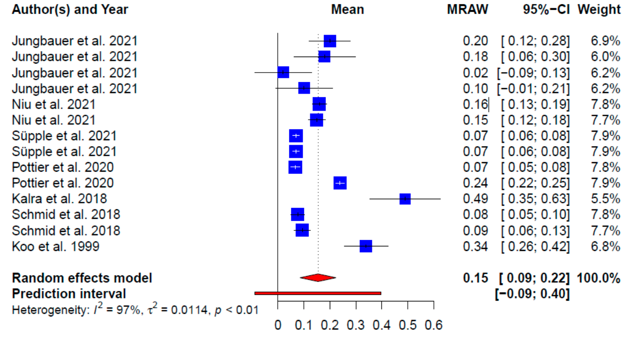 |

Premolars

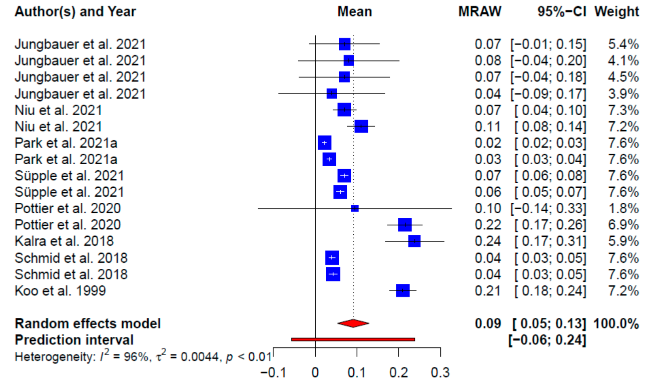


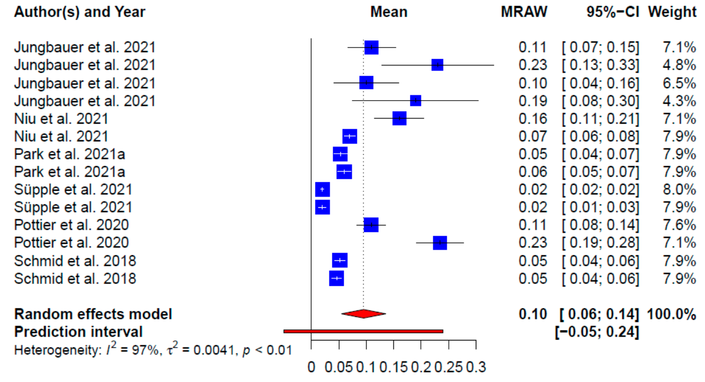


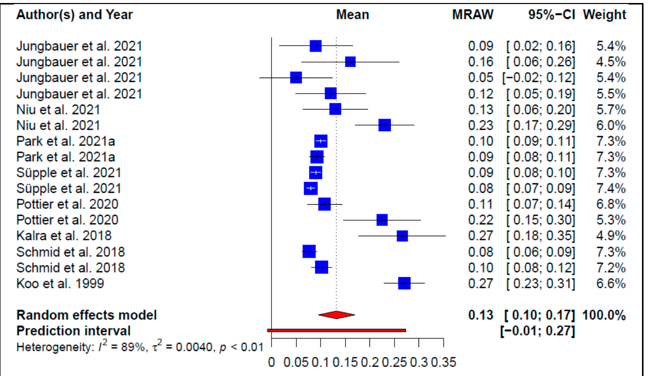


Molars

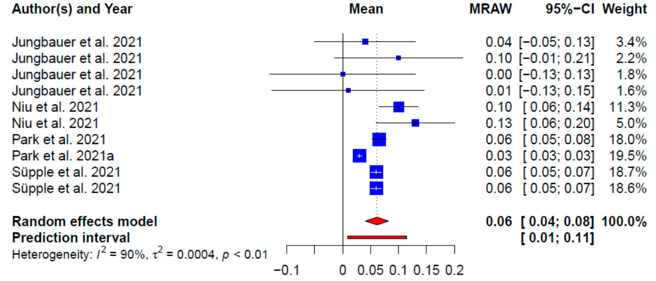


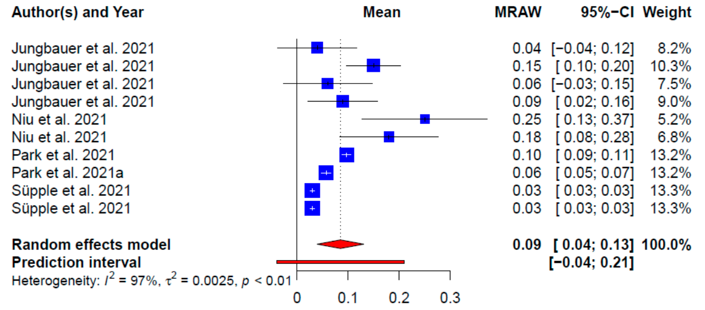


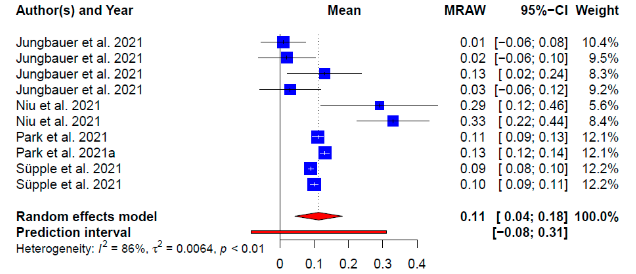


## Table S6.2. Linear parameters: Left vs. Right side

| Analysed linear parameters | Mesiodistal | Buccolingual | Vertical |
|---|---|---|---|

Left

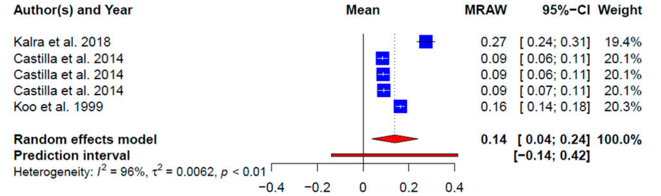


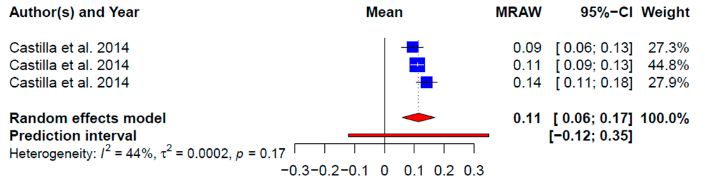


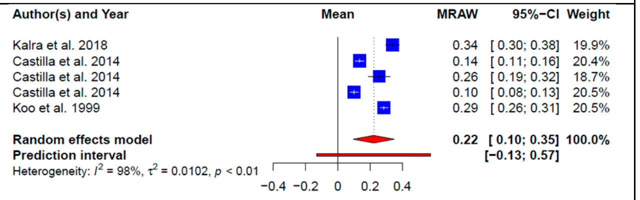

<table>
<tr><td>Right</td><td>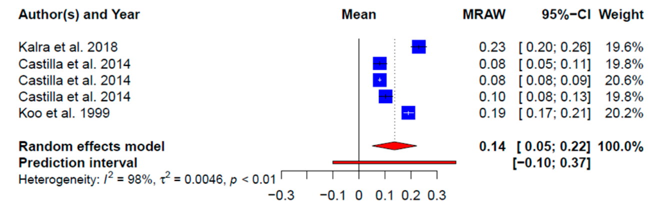
</td><td>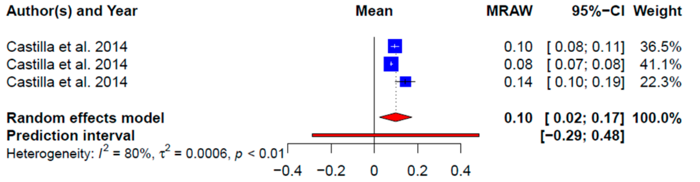
</td><td>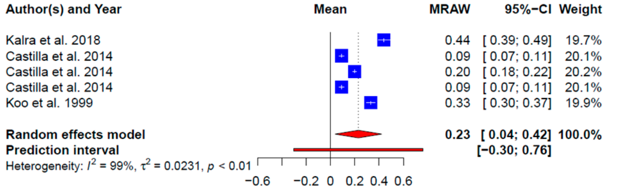
</td></tr>
</table>

## Table S6.3. Linear parameters: Upper vs. Lower jaw

<table>
<tr><th>Analysed linear parameters</th><th>Mesiodistal</th><th>Buccolingual</th><th>Vertical</th></tr>
<tr><td>Upper jaw</td><td>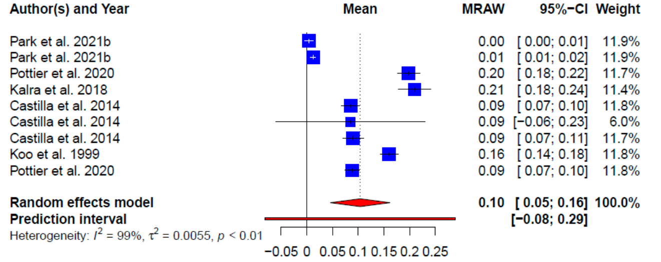
</td><td>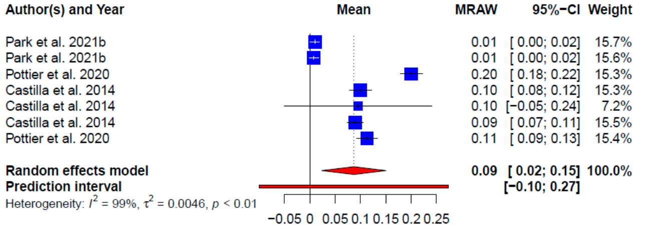
</td><td>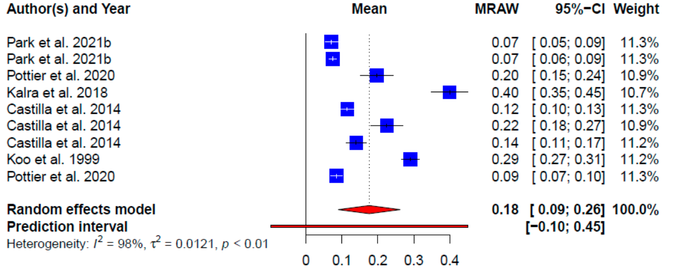
</td></tr>
<tr><td>Lower jaw</td><td>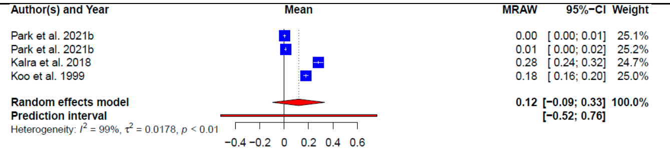
</td><td>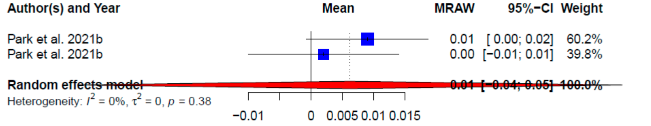
</td><td>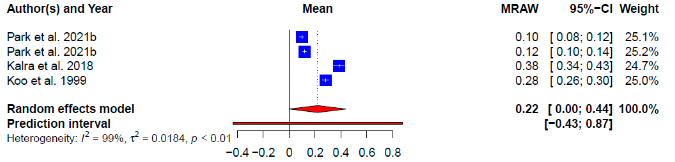
</td></tr>
</table>

## Table S6.4. Linear parameters: Accuracy assessment method

<table>
<tr><th>Analysed linear parameters</th><th>Mesiodistal</th><th>Buccolingual</th><th>Vertical</th></tr>
<tr><td>3D</td><td>
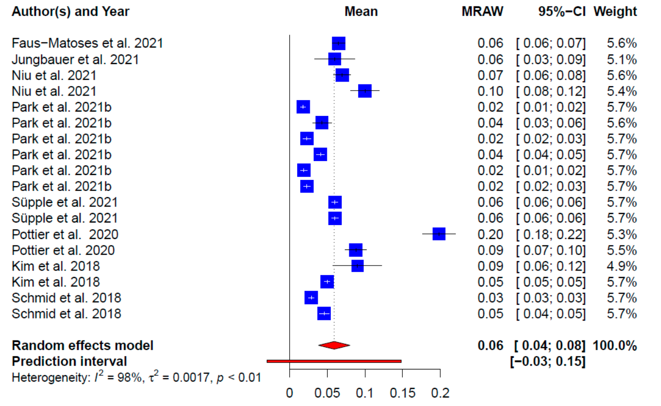

</td><td>
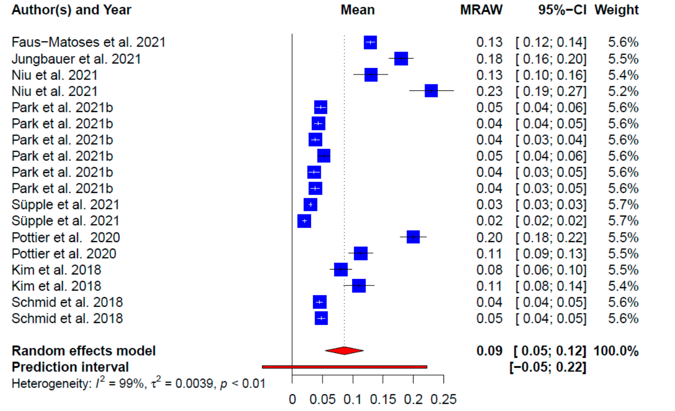

</td><td>
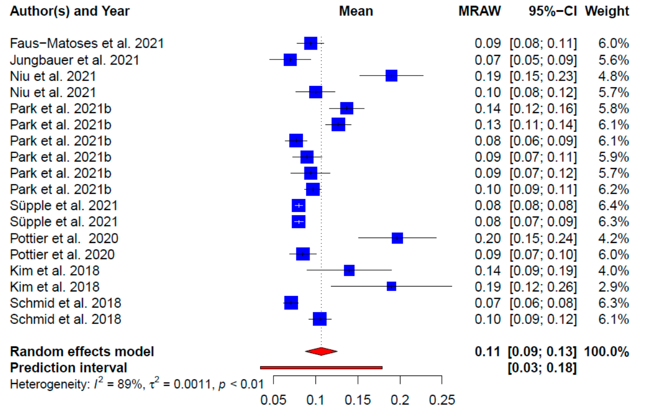

</td></tr>
<tr><td>Photography</td><td>
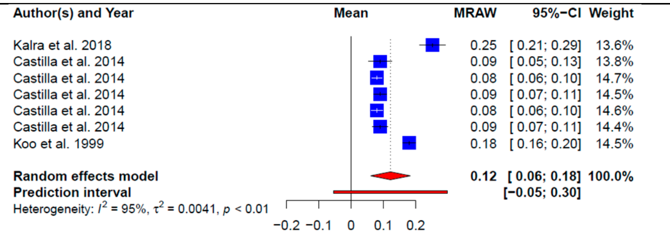

</td><td>
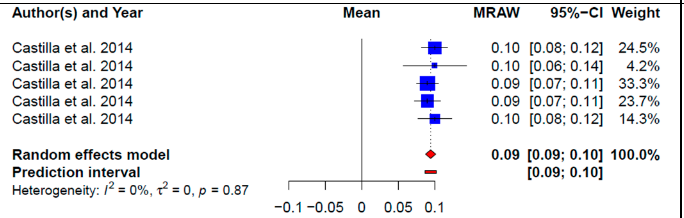

</td><td>
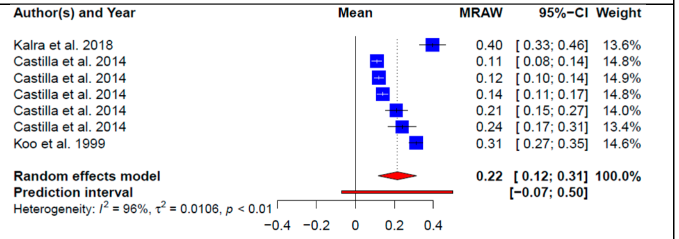

</td></tr>
</table>

# Table S6.5. Linear parameters: Type of tray

<table>
<tr><th>Analysed linear parameters</th><th>Mesiodistal</th><th>Buccolingual</th><th>Vertical</th></tr>
<tr><td>3D printed</td><td>
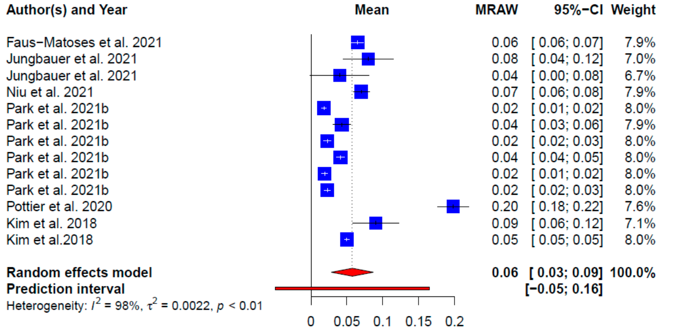

</td><td>
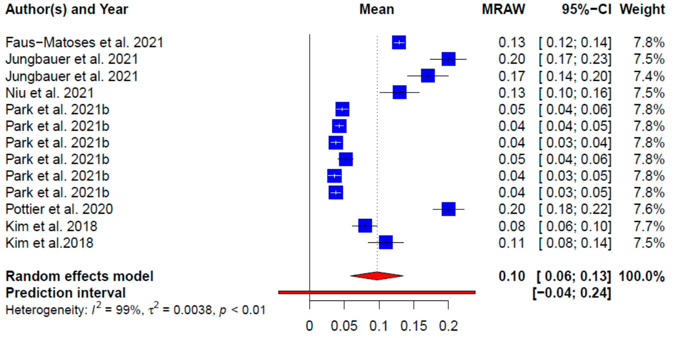

</td><td>
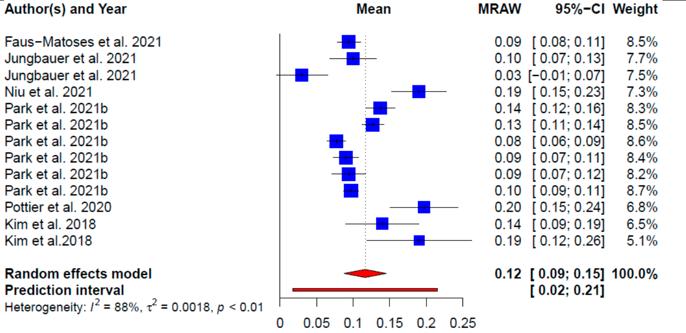

</td></tr>
<tr><td>Silicone</td><td>
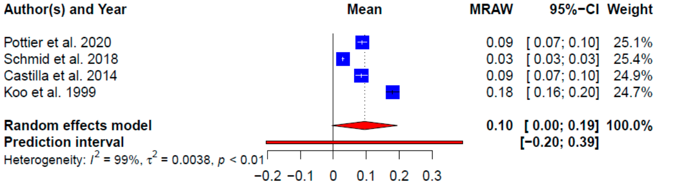

</td><td>
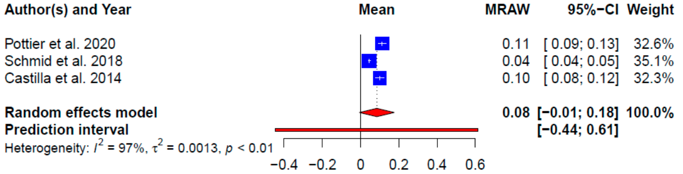

</td><td>
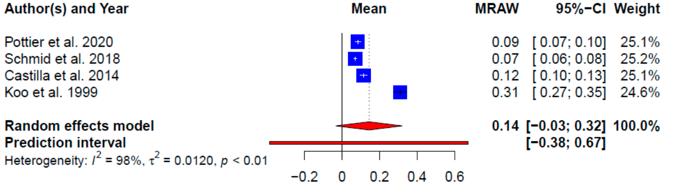

</td></tr>
<tr><td>Vacuum form</td><td>
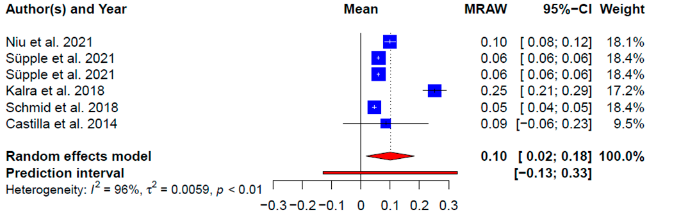

</td><td>
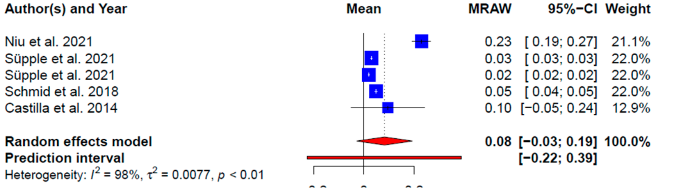

</td><td>
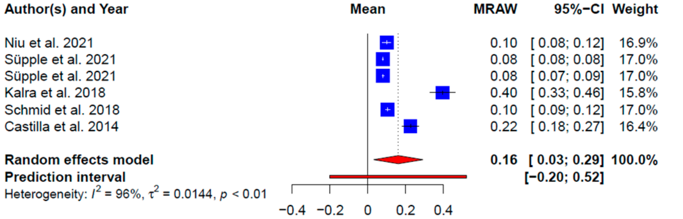

</td></tr>
<tr><td>Combined Silicone/ Vacuum Form</td><td></td><td></td><td></td></tr>
</table>

## Table S6.6. Angular parameters: Tooth group comparison

<table>
<tr><th>Analysed linear parameters</th><th>Angulation</th><th>Rotation</th><th>Torque</th></tr>
<tr><td>Incisors</td><td>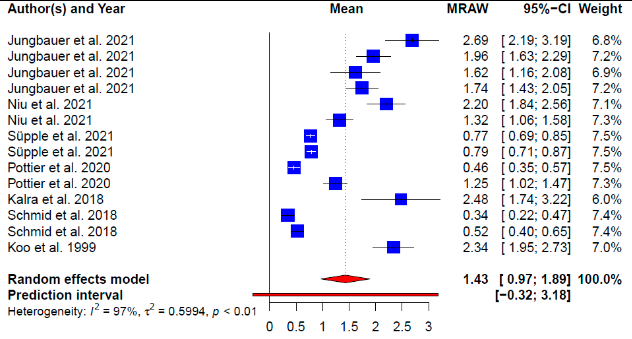
</td><td>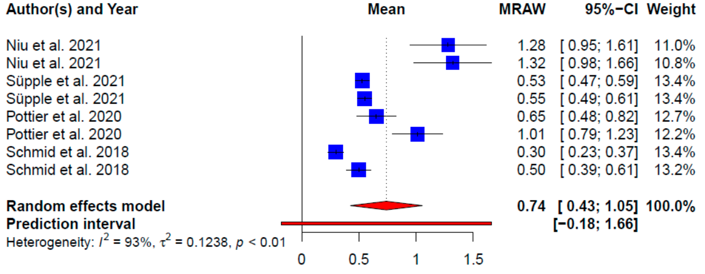
</td><td>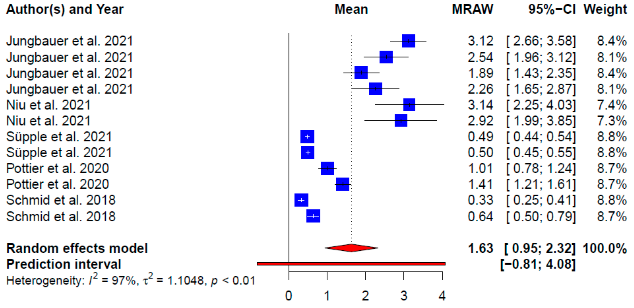
</td></tr>
<tr><td>Canines</td><td>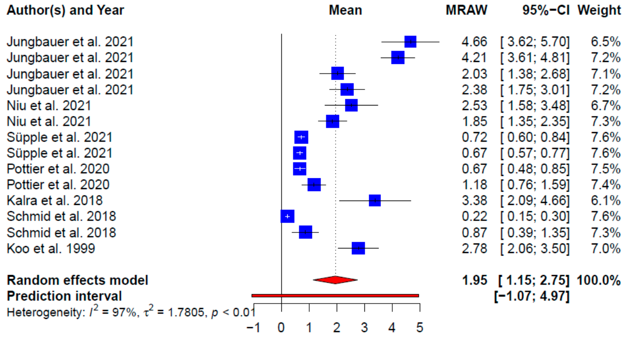
</td><td>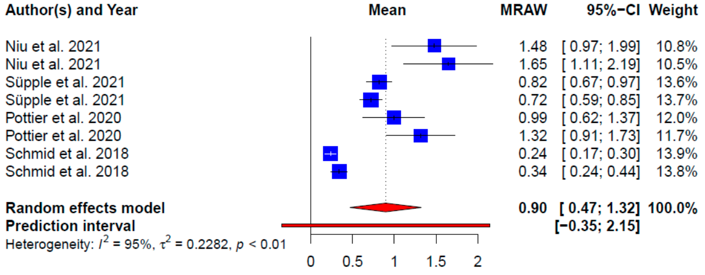
</td><td>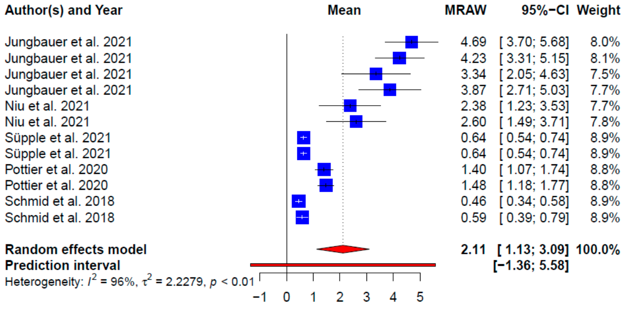
</td></tr>
<tr><td>Premolars</td><td>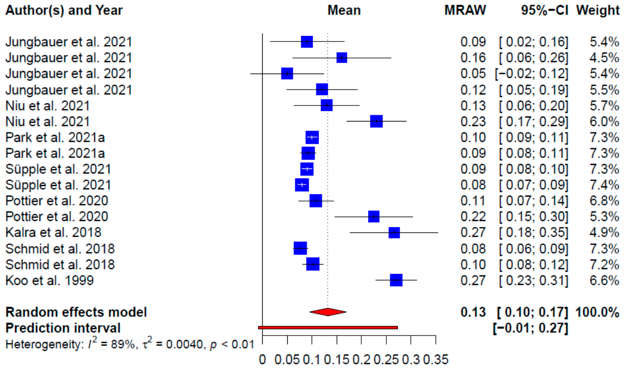
</td><td>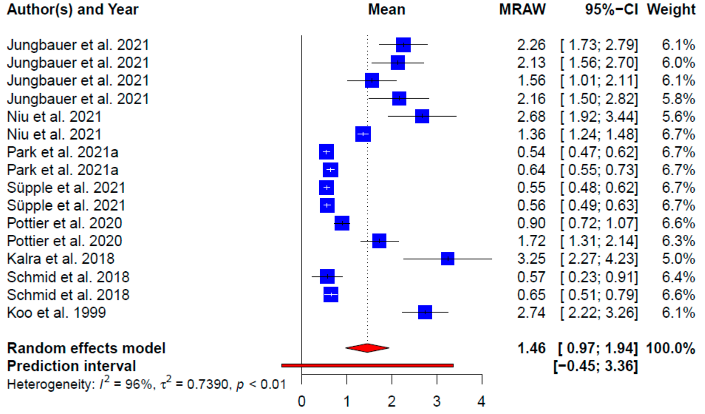
</td><td>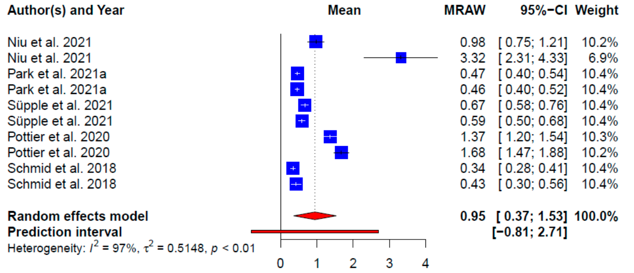
</td></tr>
</table>

| | | | |
|---|---|---|---|
| Molars | 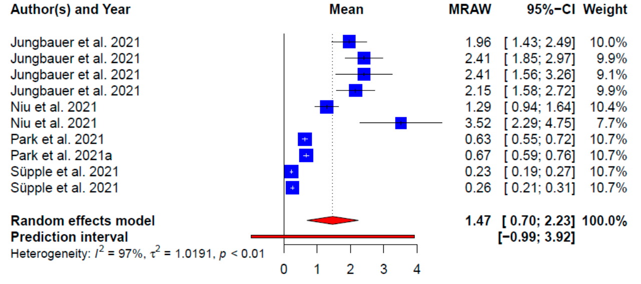 | 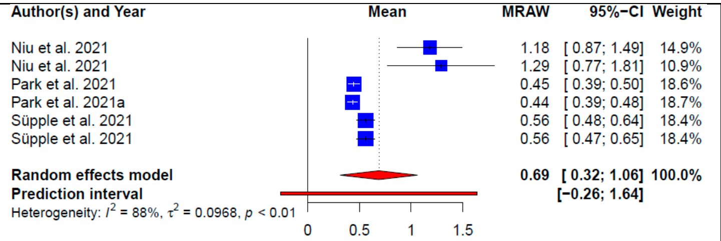 | 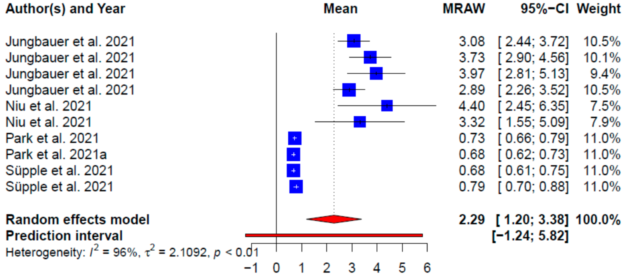 |

## Table S6.7. Angular parameters: Left vs. Right side

| Analysed linear parameters | Angulation | Rotation | Torque |
|---|---|---|---|
| Left | 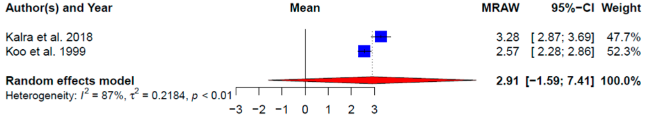 | | |
| Right | 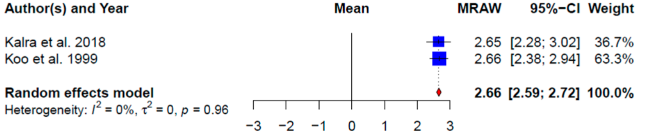 | | |

## Table S6.8. Angular parameters: Upper vs. Lower jaw

| Analysed linear parameters | Angulation | Rotation | Torque |
|---|---|---|---|
| Upper jaw | 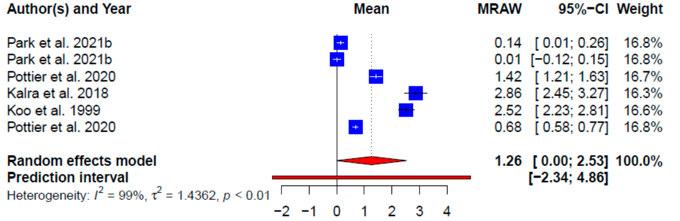 | 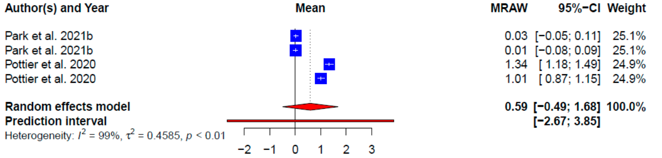 | 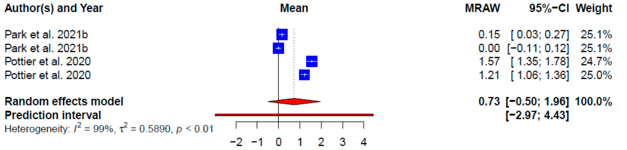 |

Lower jaw

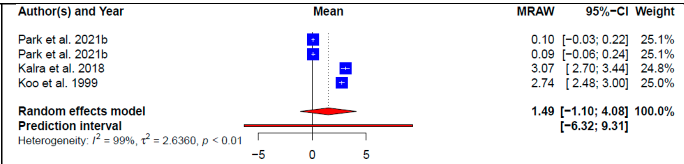


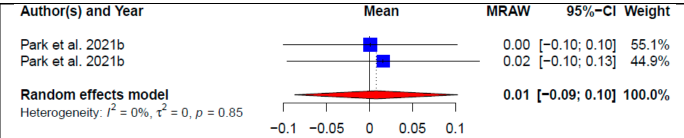


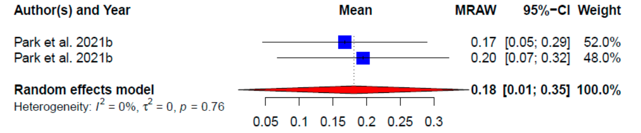


## Table S6.9. Angular parameters: Accuracy assessment method

| **Analysed linear parameters** | Angulation | Rotation | Torque |
|---|---|---|---|
| 3D | see below | see below | see below |
| Photography | see below | | |

**3D – Angulation**

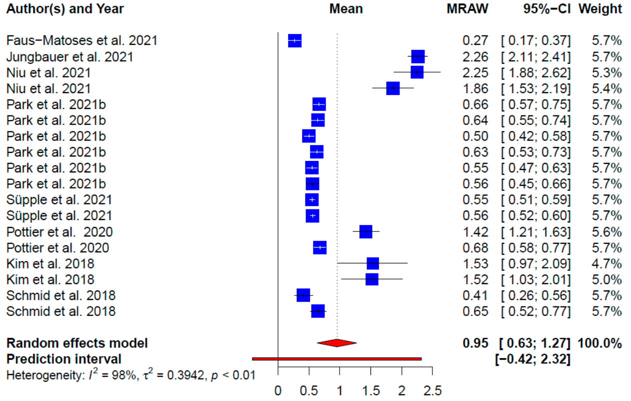


**3D – Rotation**

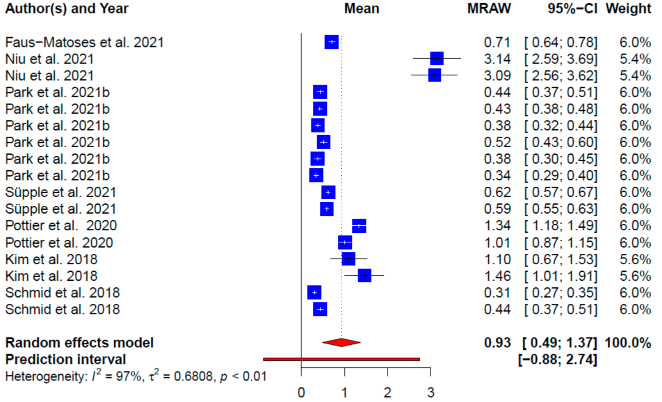


**3D – Torque**

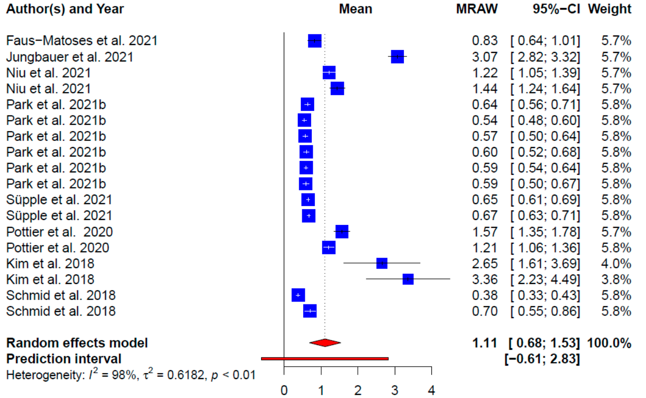


**Photography – Angulation**

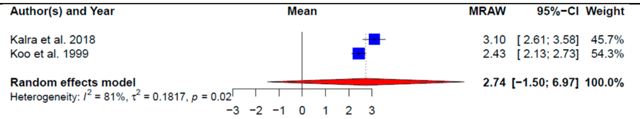

# Table S6.10. Angular parameters: Type of tray

<table>
<tr><th>Analysed linear parameters</th><th>Angulation</th><th>Rotation</th><th>Torque</th></tr>
<tr><td>3D printed</td><td>
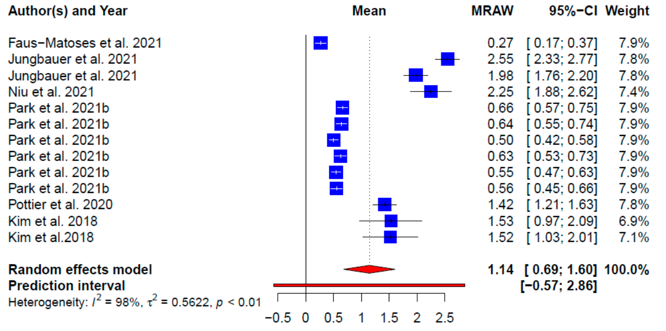

</td><td>
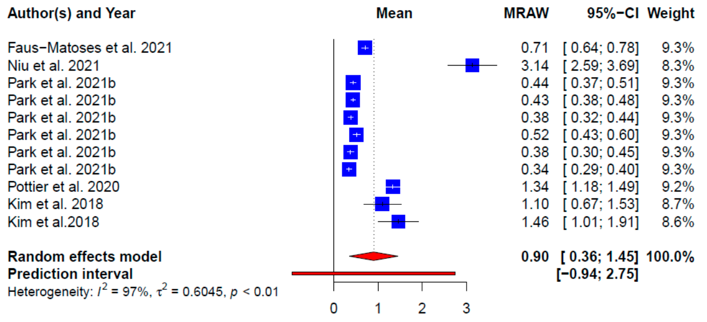

</td><td>
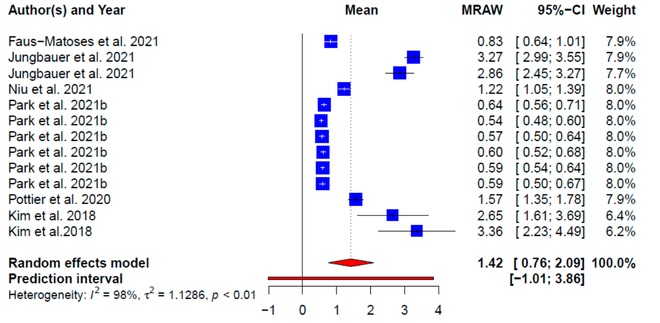

</td></tr>
<tr><td>Silicone</td><td>
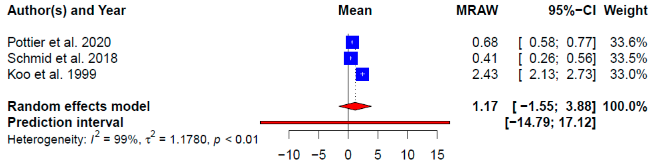

</td><td>
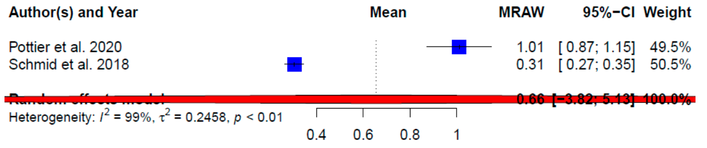

</td><td>
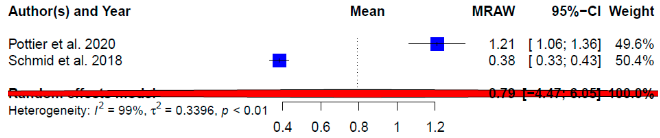

</td></tr>
<tr><td>Vacuum form</td><td>
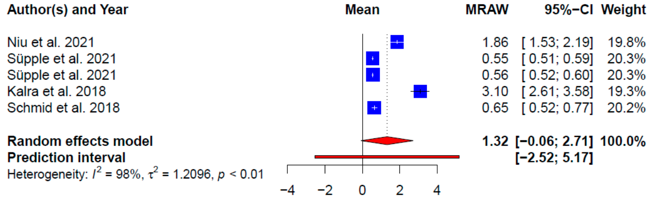

</td><td>
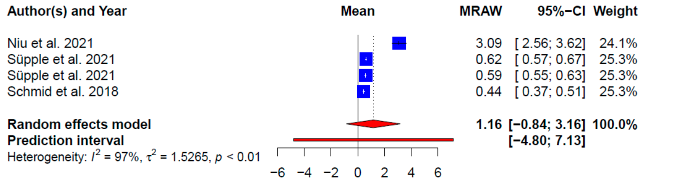

</td><td>
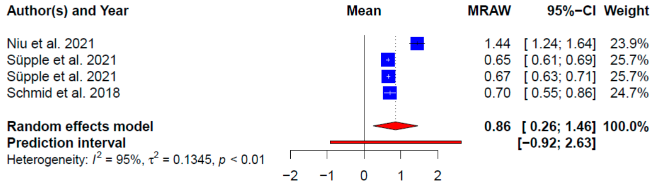

</td></tr>
<tr><td>Combined Silicone/ Vacuum Form</td><td></td><td></td><td></td></tr>
</table>

# Meta analytic statistics – Funnel Plots

## Table S7.1. Linear parameters: Overall accuracy

| Analysed parameters | Mesiodistal | Buccolingual | Vertical |
|---|---|---|---|
| Overall accuracy | 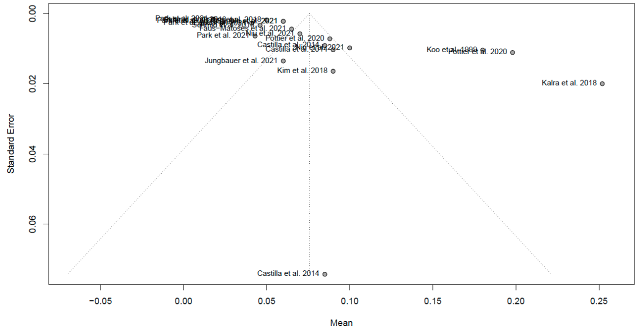 | 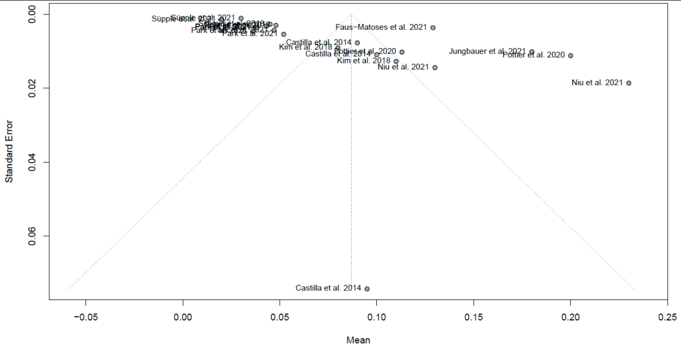 | 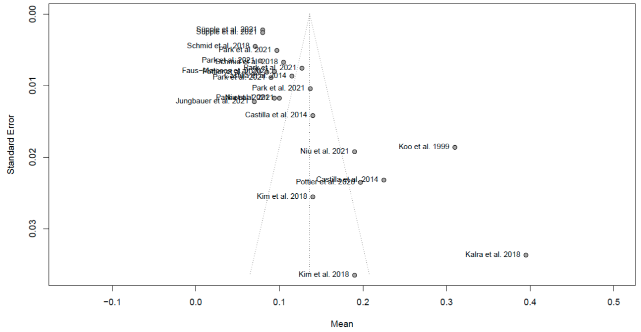 |

## Table S7.2. Linear parameters: Tooth group comparison

| Analysed linear parameters | Mesiodistal | Buccolingual | Vertical |
|---|---|---|---|
| Incisors | 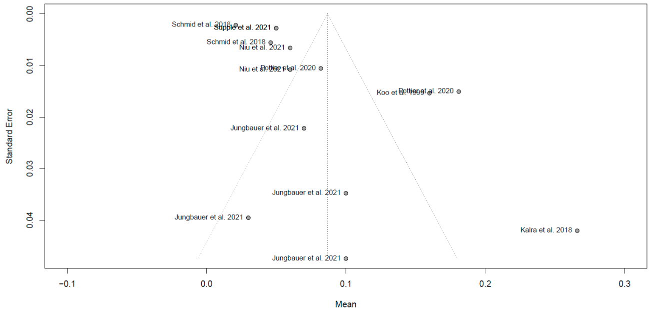 | 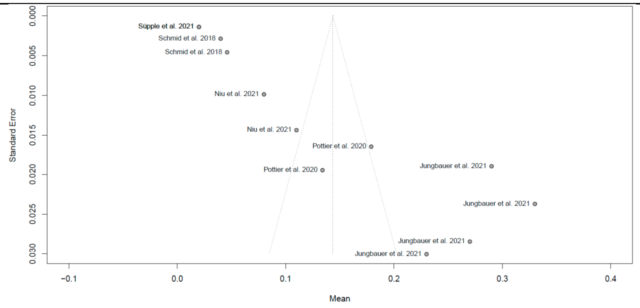 | 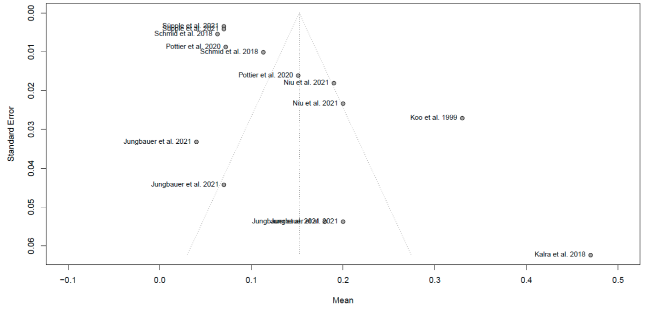 |

Canines

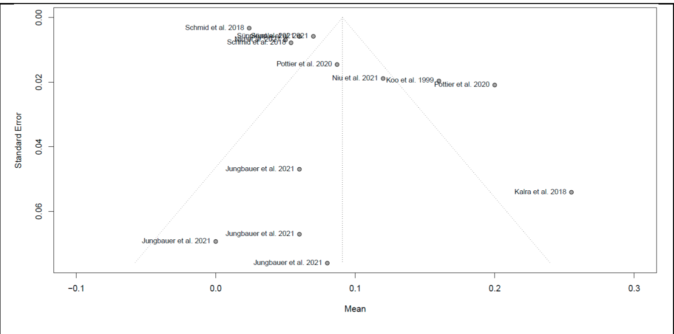


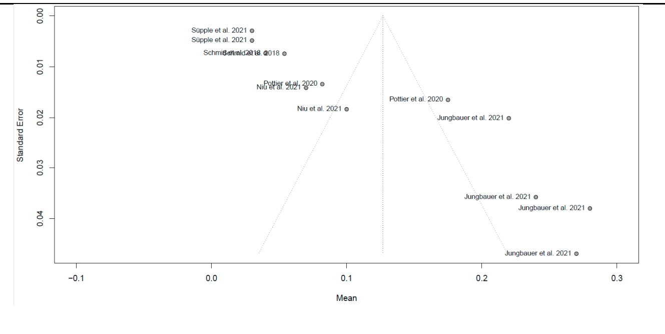


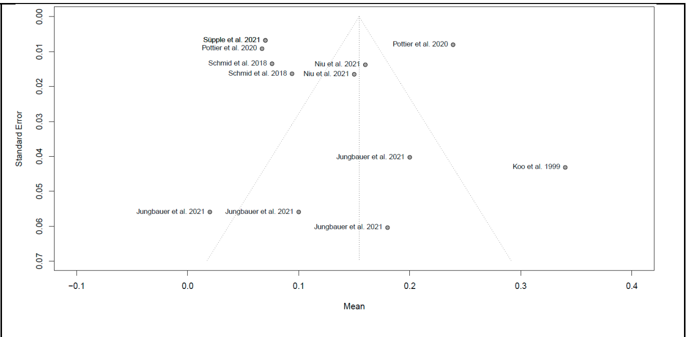


Premolars

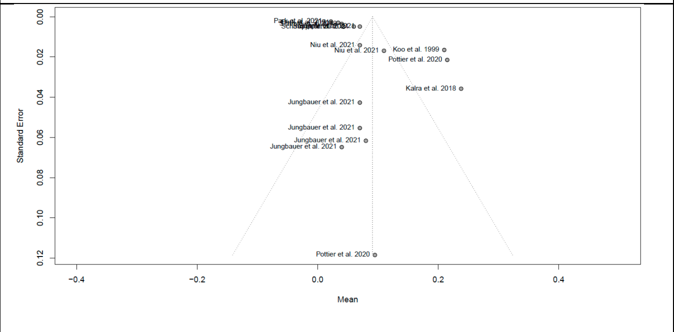


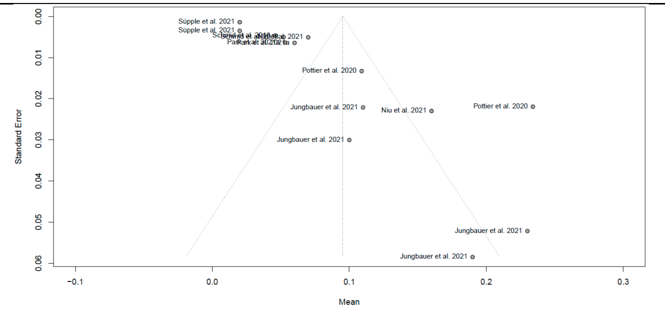


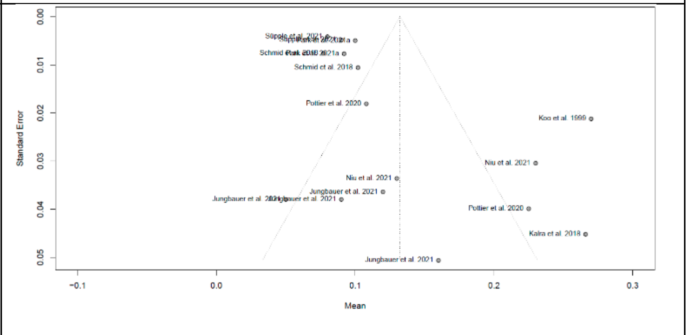


Molars

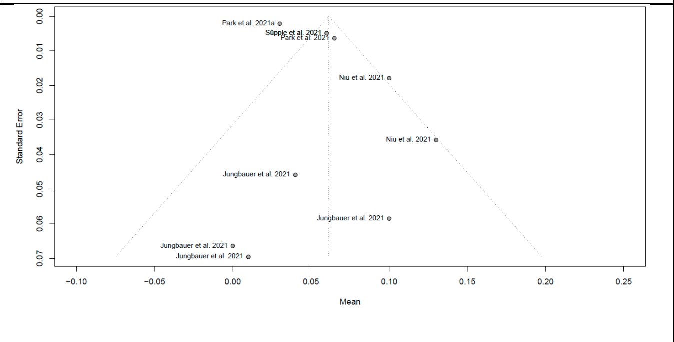


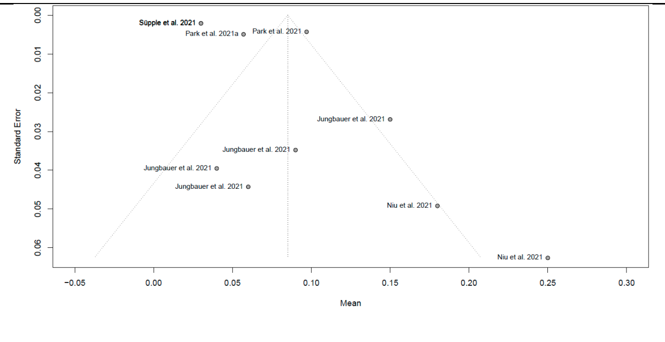


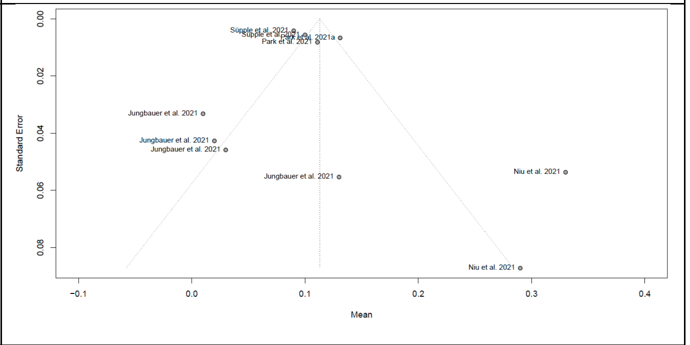

## Table S7.3. Linear parameters: Left vs. Right side

<table>
<tr><th>Analysed linear parameters</th><th>Mesiodistal</th><th>Buccolingual</th><th>Vertical</th></tr>
<tr><td>Left</td><td>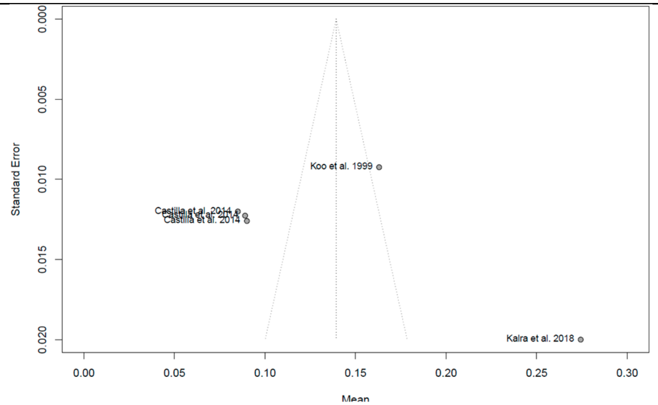
</td><td>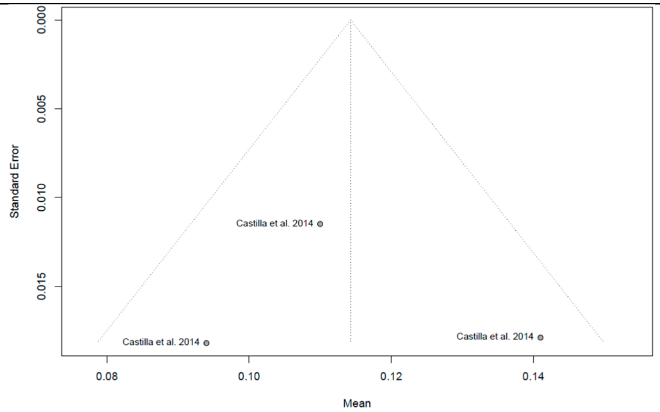
</td><td>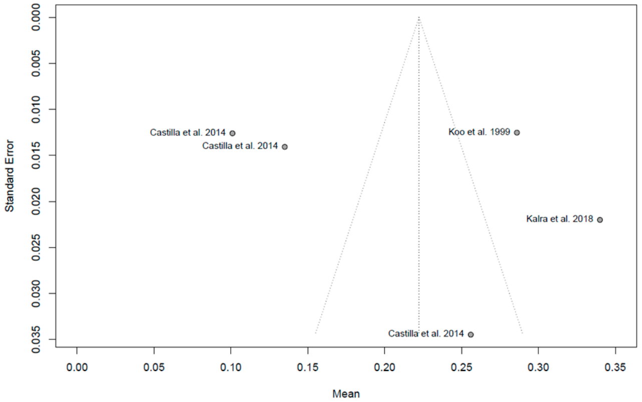
</td></tr>
<tr><td>Right</td><td>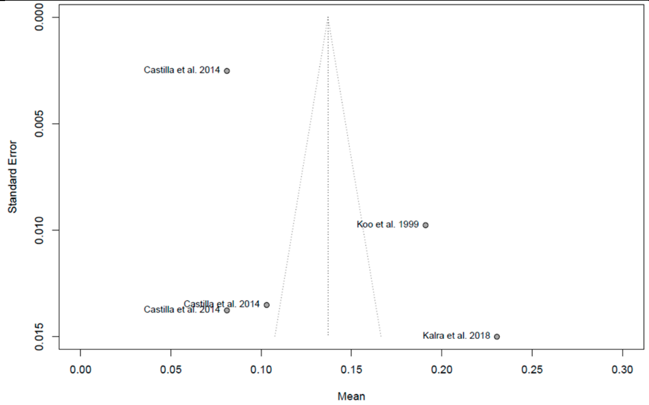
</td><td>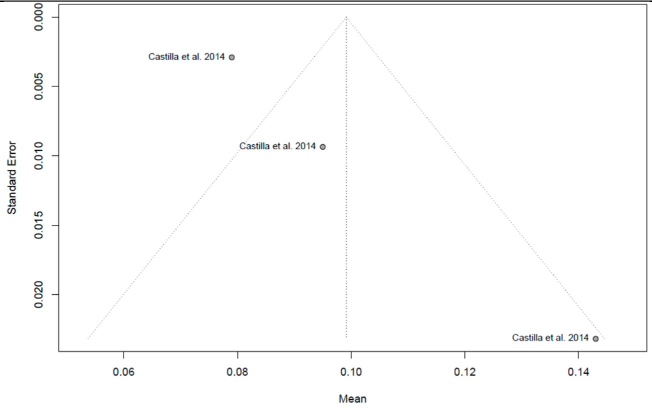
</td><td>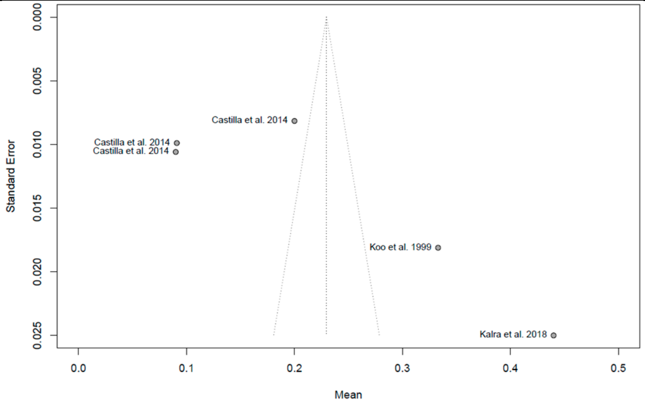
</td></tr>
</table>

## Table S7.4. Linear parameters: Upper vs. Lower jaw

| Analysed linear parameters | Mesiodistal | Buccolingual | Vertical |
|---|---|---|---|
| Upper jaw | 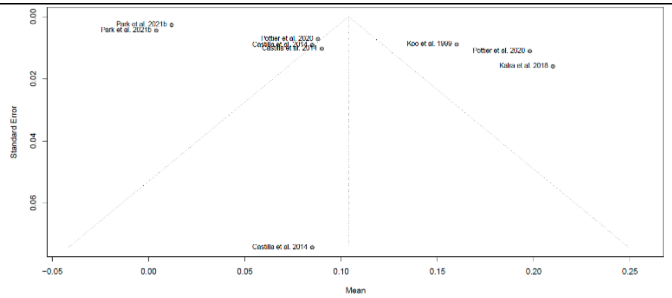 | 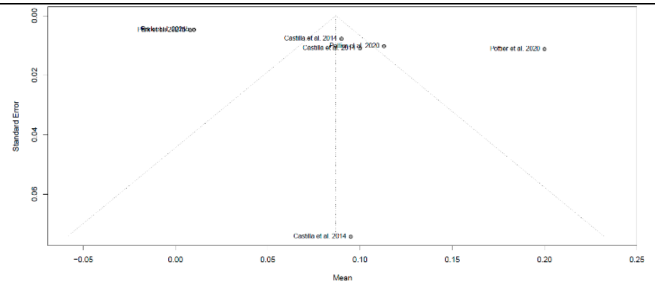 | 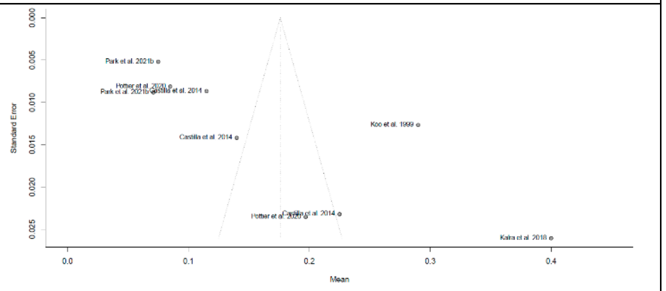 |
| Lower jaw | 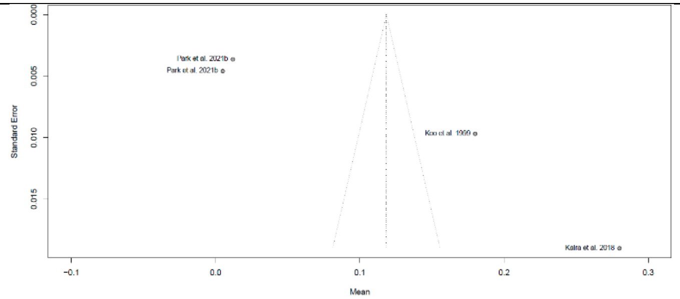 | 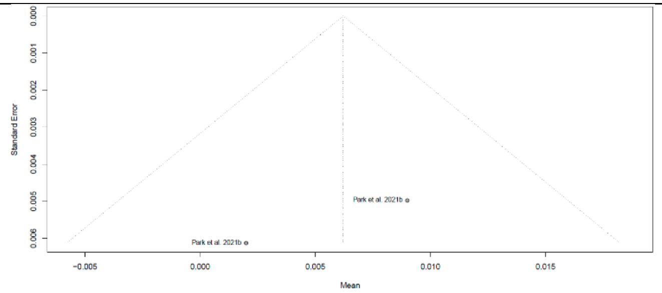 | 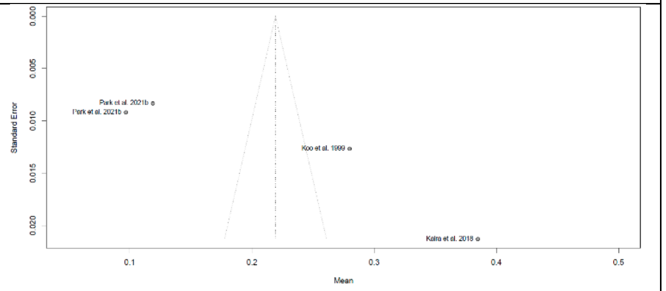 |

# Table 7.5. Linear parameters: Accuracy assessment method

<table>
<tr><th>Analysed linear parameters</th><th>Mesiodistal</th><th>Buccolingual</th><th>Vertical</th></tr>
<tr><td>3D</td><td>
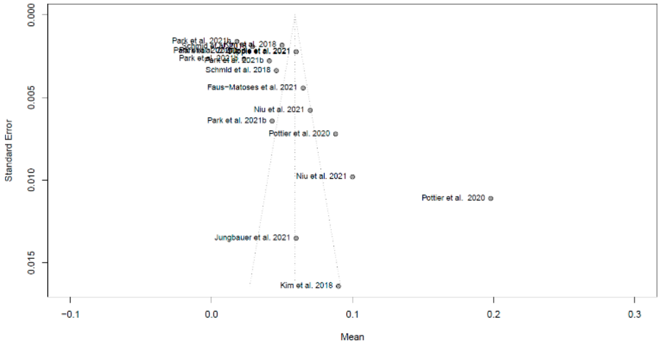

</td><td>
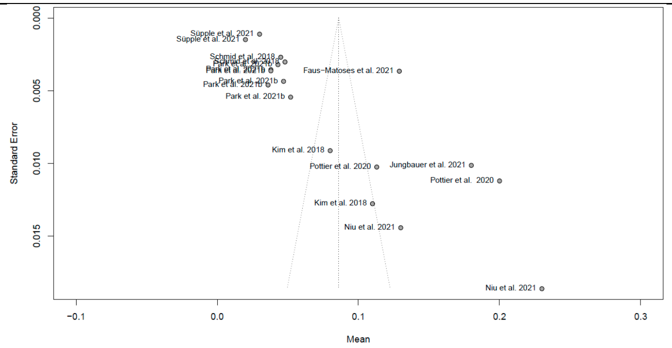

</td><td>
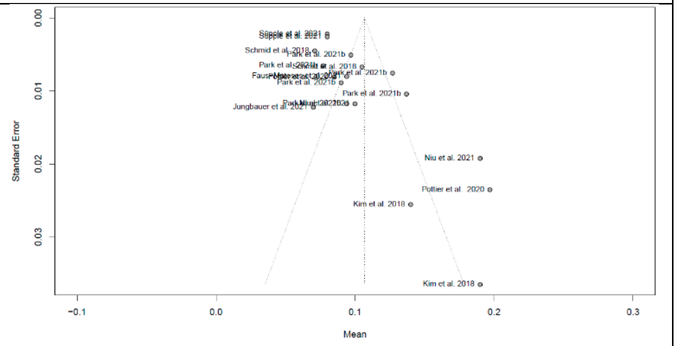

</td></tr>
<tr><td>Photography</td><td>
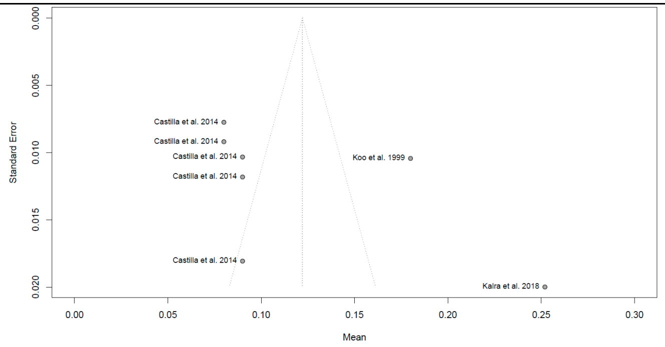

</td><td>
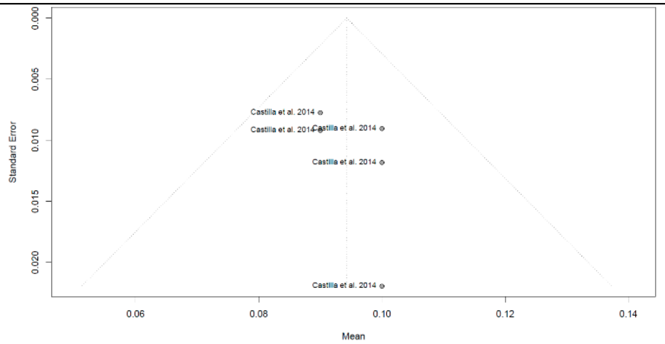

</td><td>
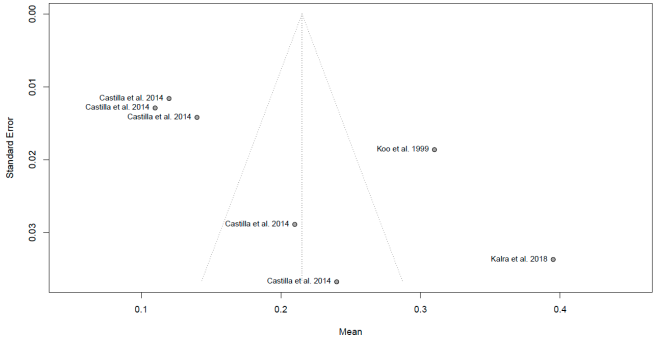

</td></tr>
</table>

## Table 7.6. Linear parameters: Type of tray

| Analysed linear parameters | Mesiodistal | Buccolingual | Vertical |
|---|---|---|---|
| 3D printed | 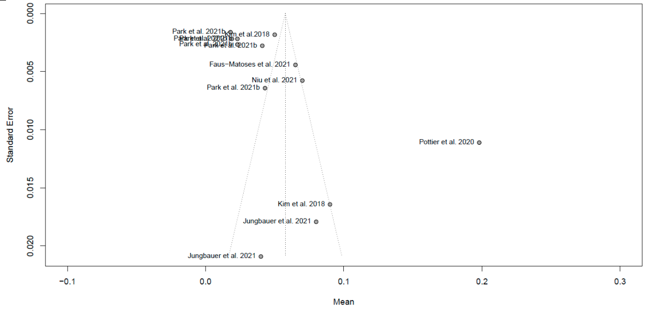 | 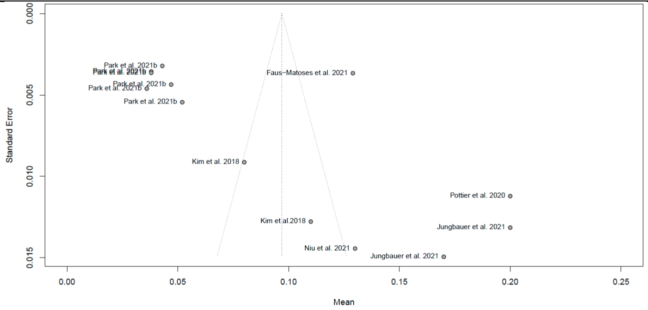 | 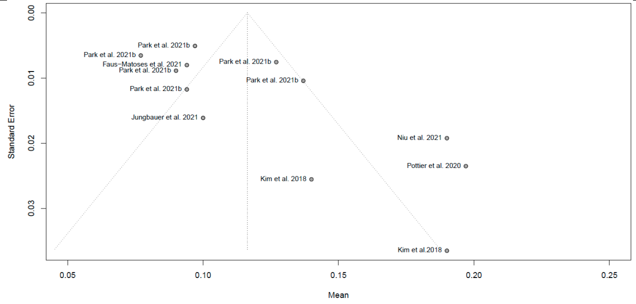 |
| Silicone | 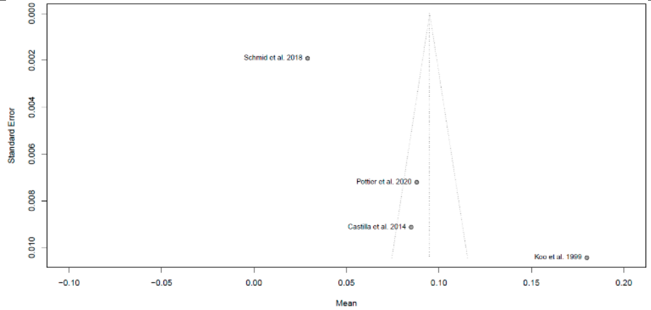 | 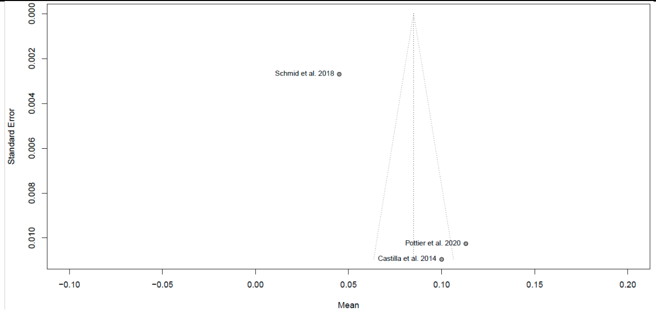 | 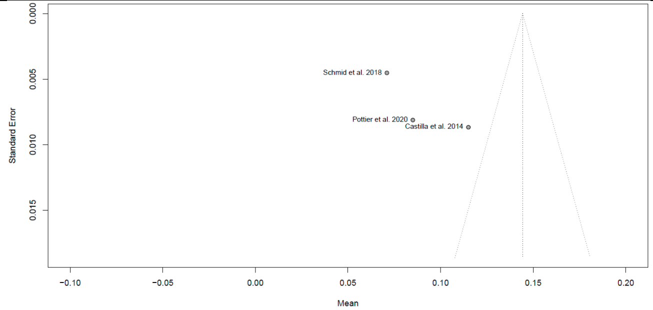 |
| Vacuum form | 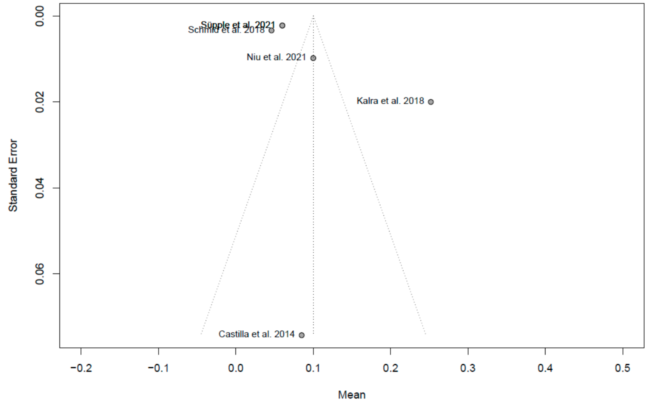 | 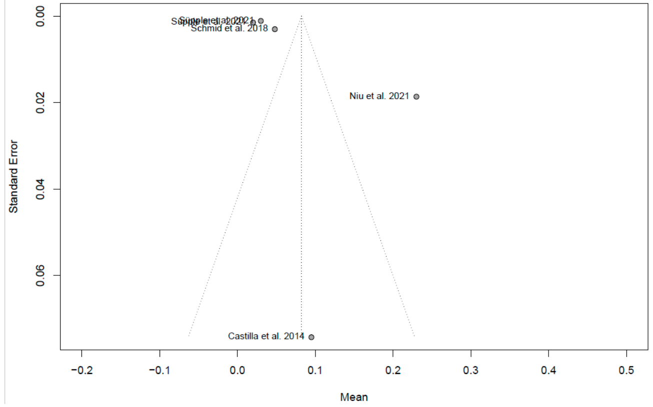 | 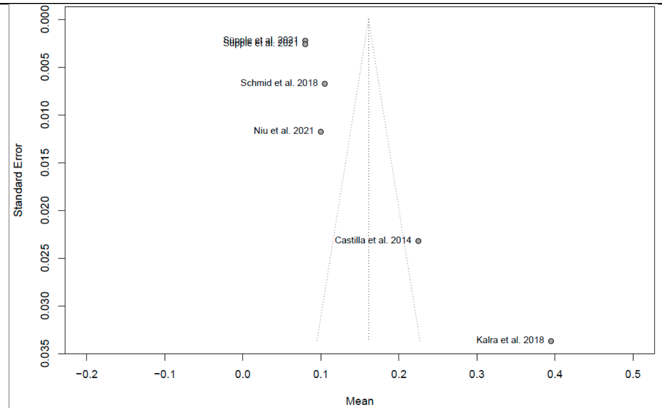 |
| Combined Silicone/ | | | |

| | | | |
|---|---|---|---|
| Vacuum Form | | | |

## Table S7.7. Angular parameters: Overall accuracy

| Analysed parameters | Angulation | Rotation | Torque |
|---|---|---|---|
| **Overall accuracy** | 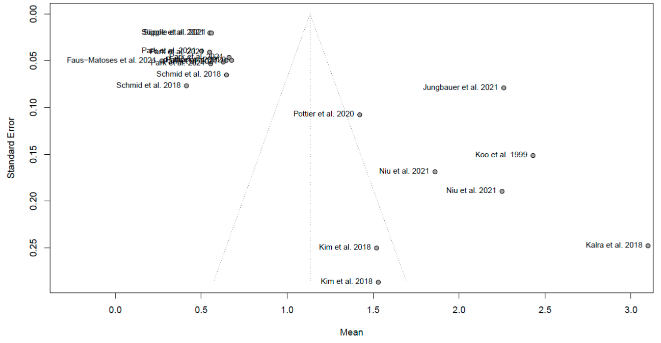 | 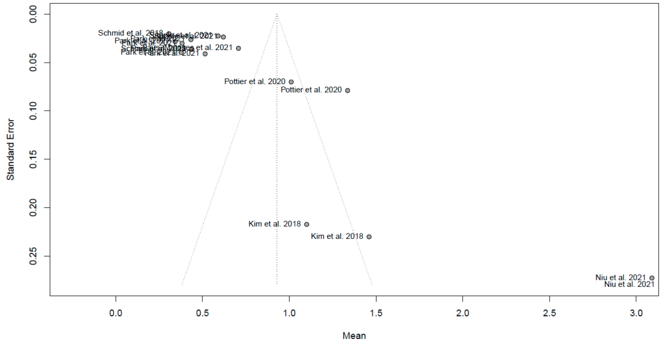 | 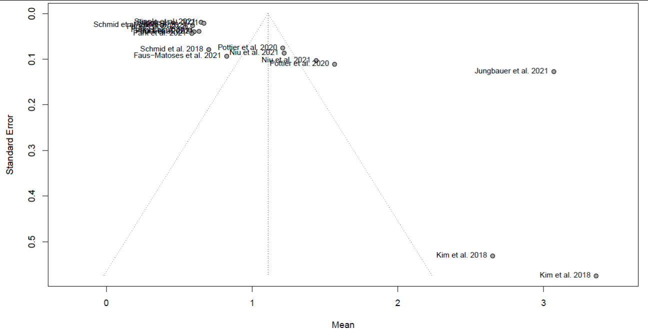 |

## Table S7.8. Angular parameters: Tooth group comparison

| Analysed linear parameters | Angulation | Rotation | Torque |
|---|---|---|---|
| Incisors | 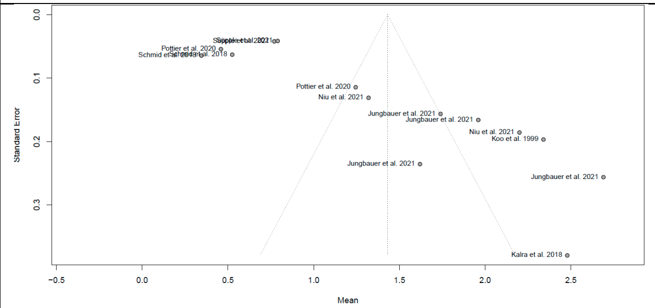  | 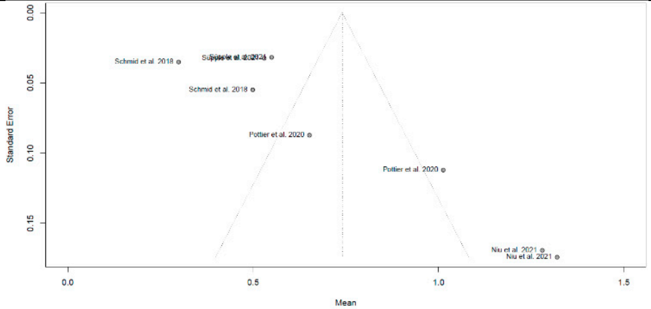  | 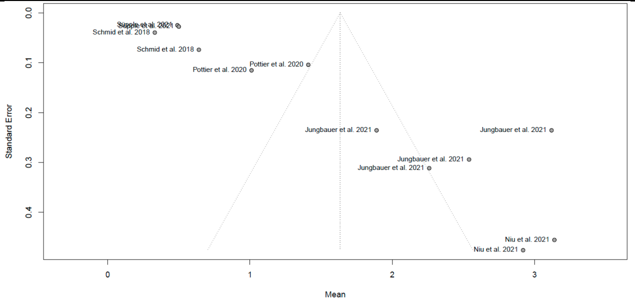  |
| Canines | 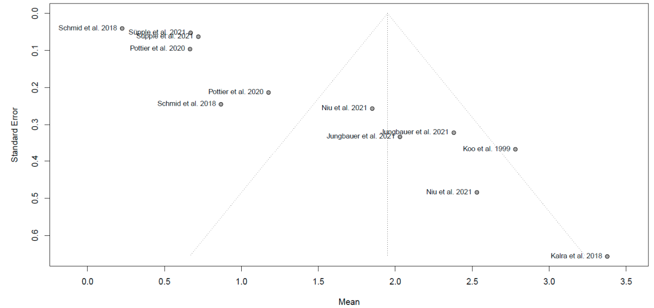  | 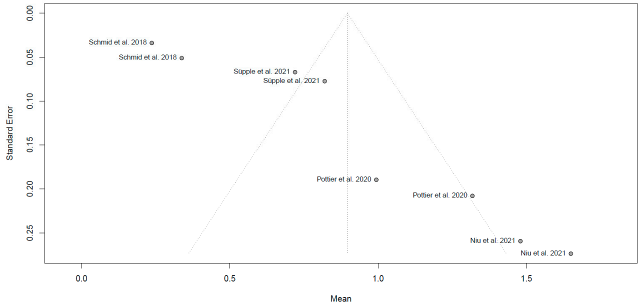  | 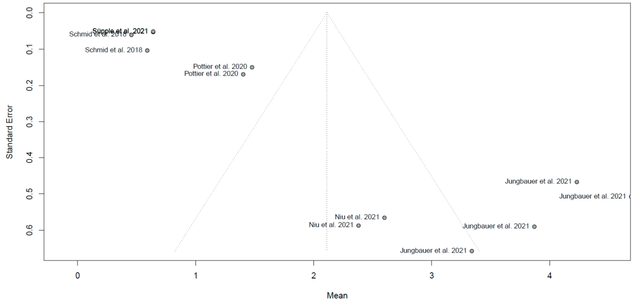  |
| Premolars | 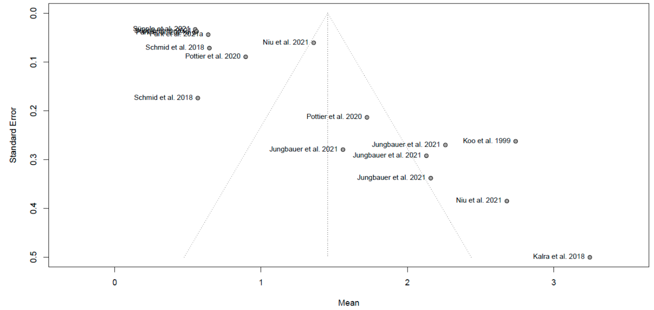  | 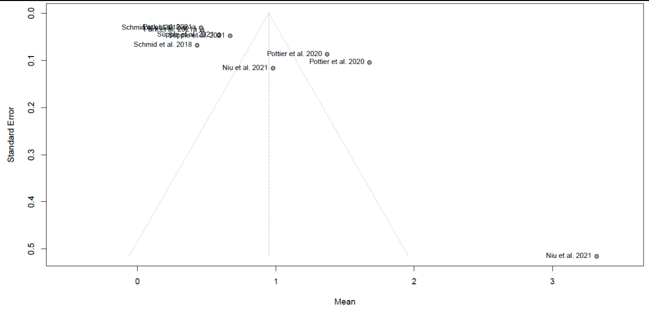  | 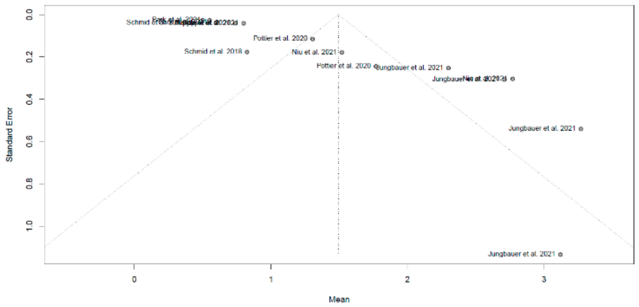  |

| | | | |
|---|---|---|---|
| Molars | 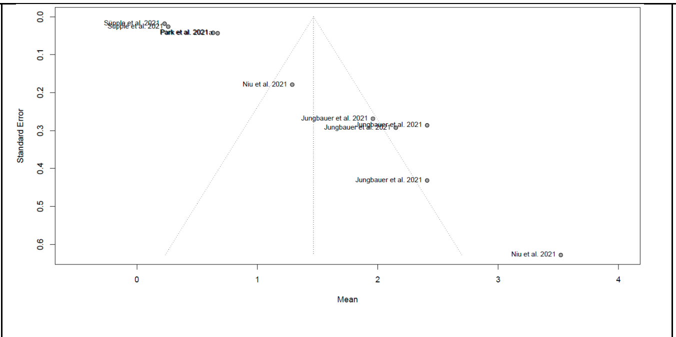 | 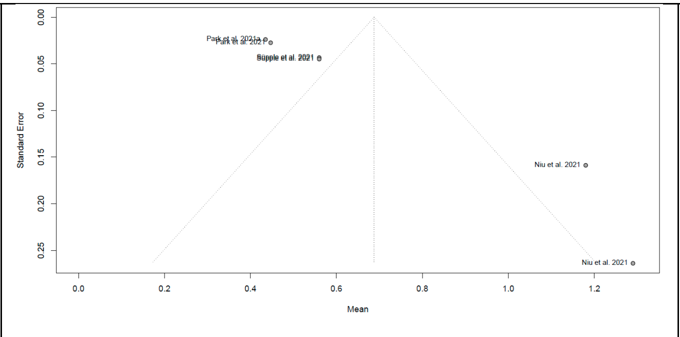 | 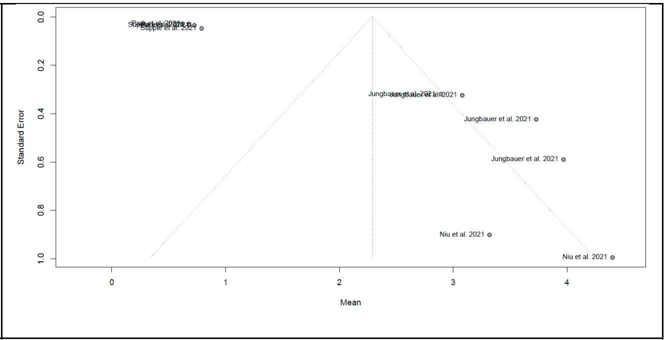 |

## Table S7.9. Angular parameters: Left vs. Right side

| Analysed linear parameters | Angulation | Rotation | Torque |
|---|---|---|---|
| Left | | | |
| Right | | | |

## Table S7.10. Angular parameters: Upper vs. Lower jaw

| Analysed linear parameters | Angulation | Rotation | Torque |
|---|---|---|---|
| Upper jaw | 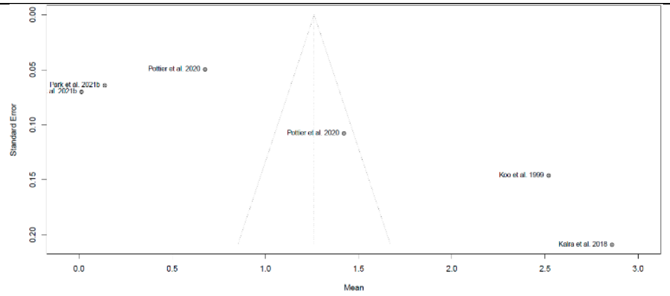 | 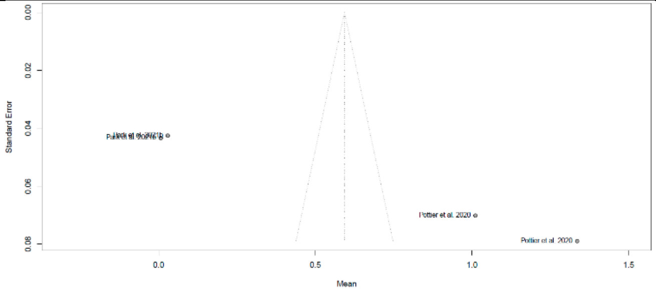 | 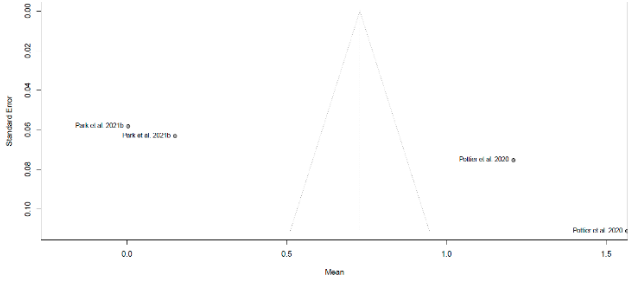 |
| Lower jaw | 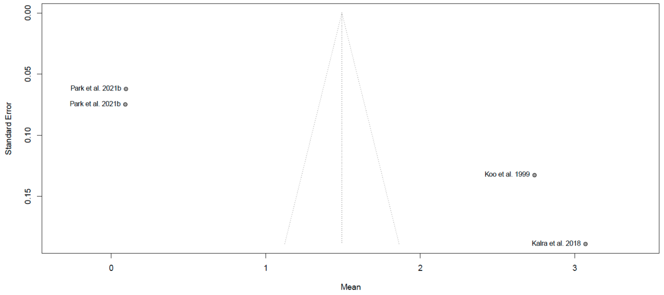 | 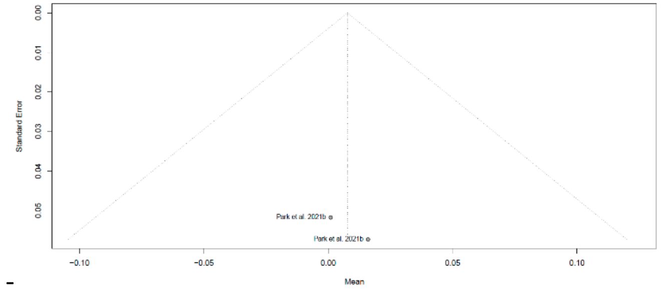 - | 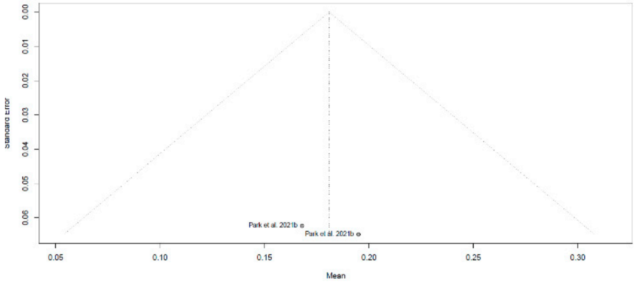 |

# Table S7.11. Angular parameters: Accuracy assessment method

| **Analysed linear parameters** | Angulation | Rotation | Torque |
|---|---|---|---|
| 3D | 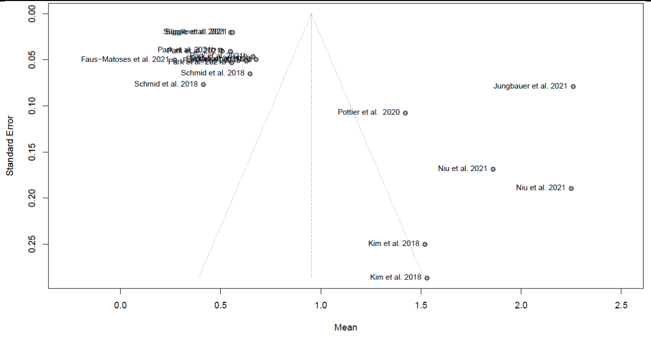 | 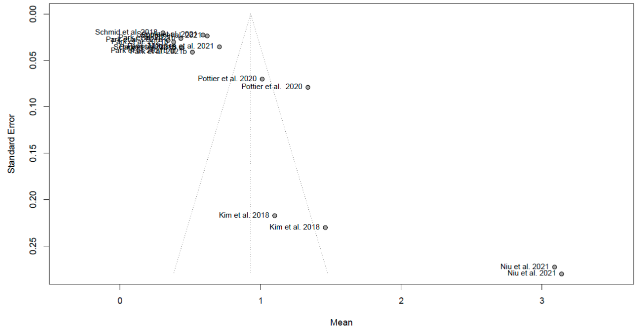 | 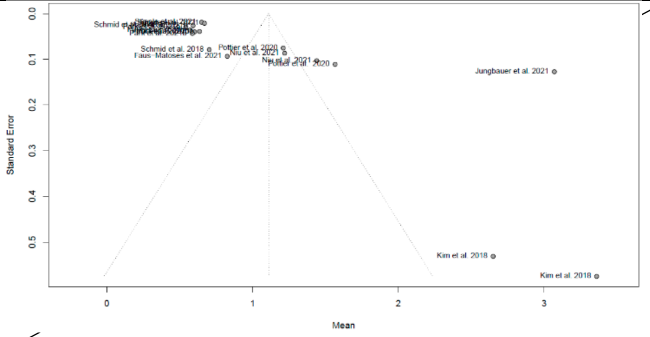 |
| Photography | 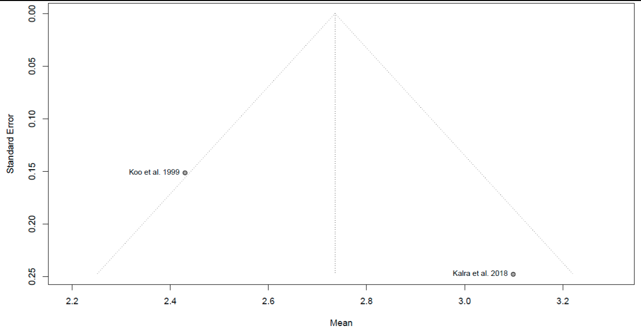 | | |

# Table S7.12. Angular parameters: Type of tray

| Analysed linear parameters | Angulation | Rotation | Torque |
|---|---|---|---|
| 3D printed | 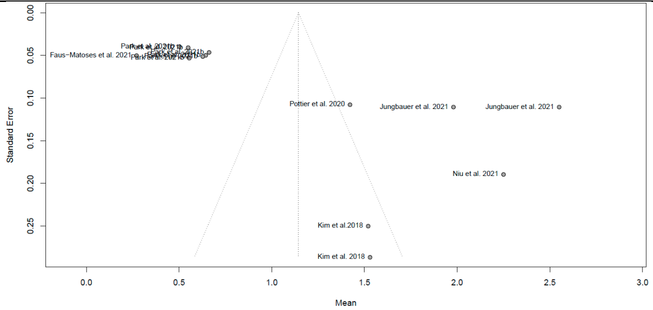  | 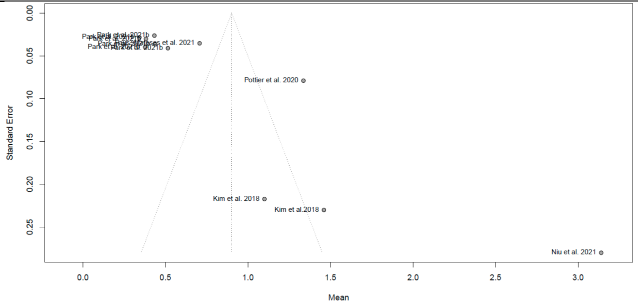  | 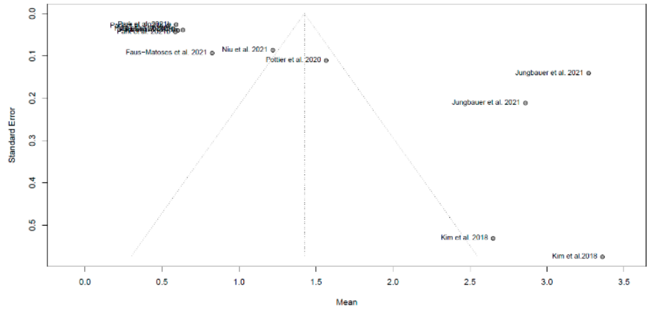  |
| Silicone | 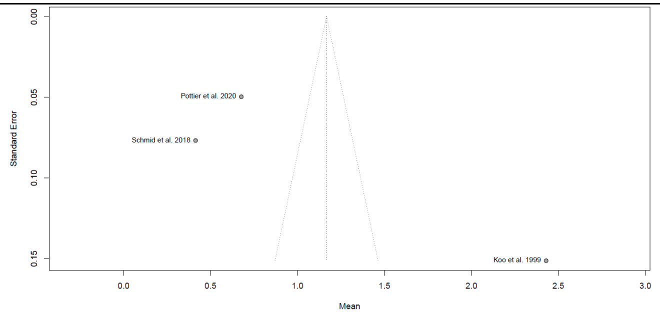  | 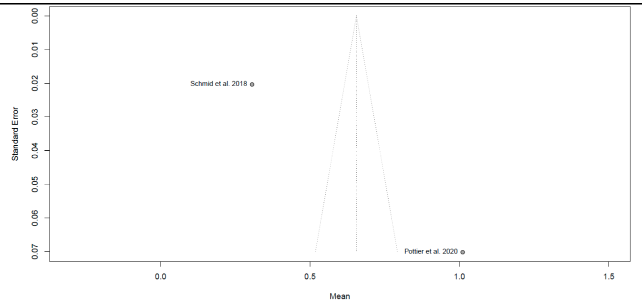  | 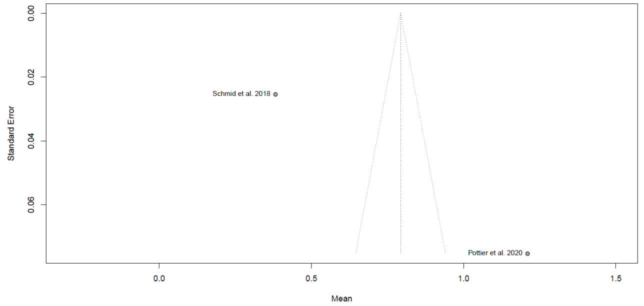  |
| Vacuum form | 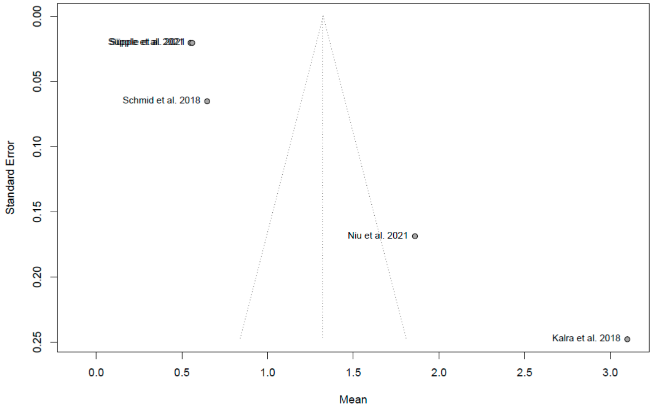  | 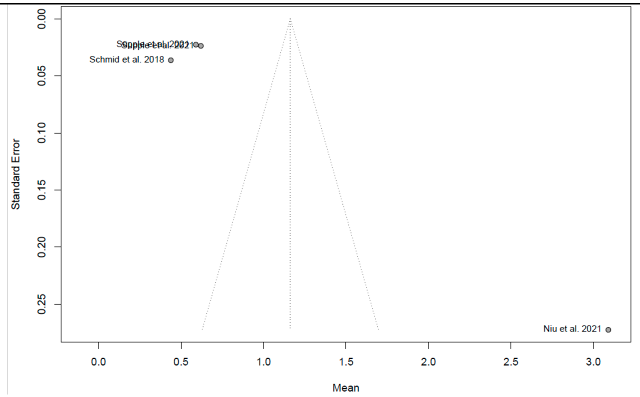  | 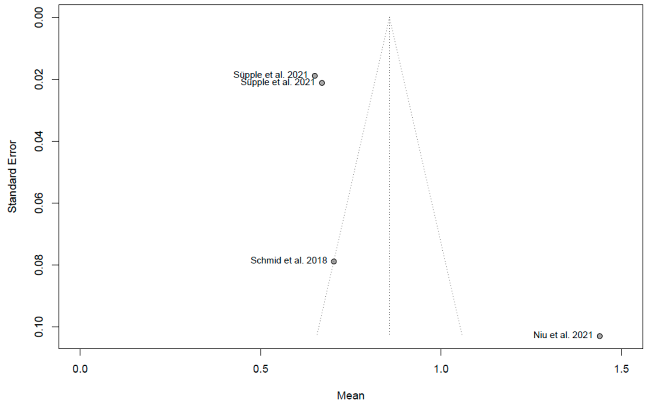  |
| Combined Silicone/ | | | |

| | | | |
|---|---|---|---|
| Vacuum Form | | | |

# Meta analytic statistics – Drapery Plots

## Table S8.1. Linear parameters: Overall accuracy

| Analysed parameters | Mesiodistal | Buccolingual | Vertical |
|---|---|---|---|
| Overall accuracy | 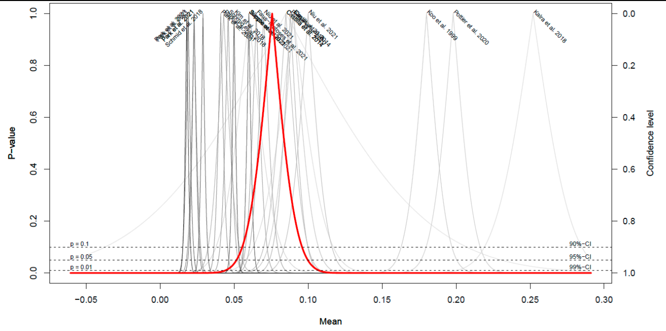 | 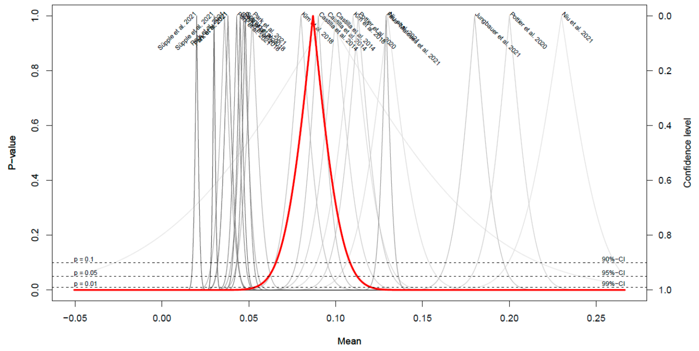 | 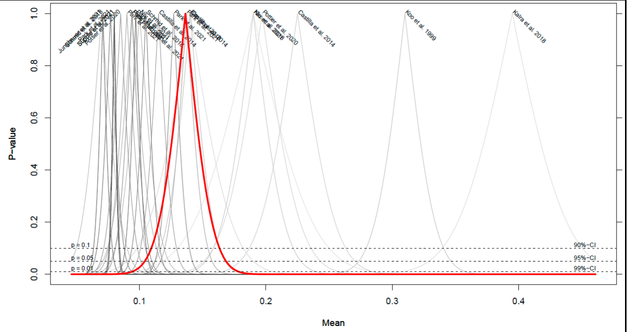 |

## Table S8.2. Linear parameters: Tooth group comparison

| Analysed linear parameters | Mesiodistal | Buccolingual | Vertical |
|---|---|---|---|
| Incisors | 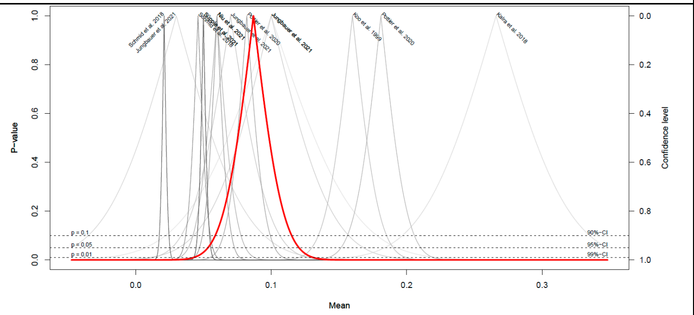 | 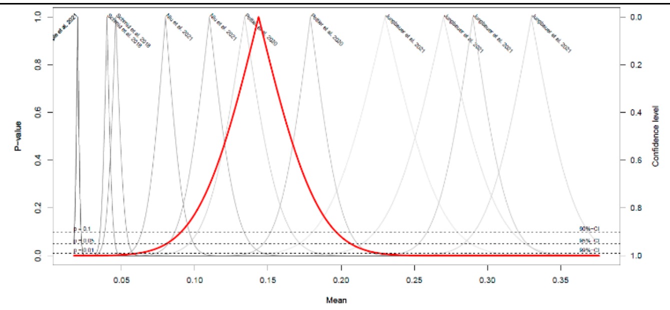 | 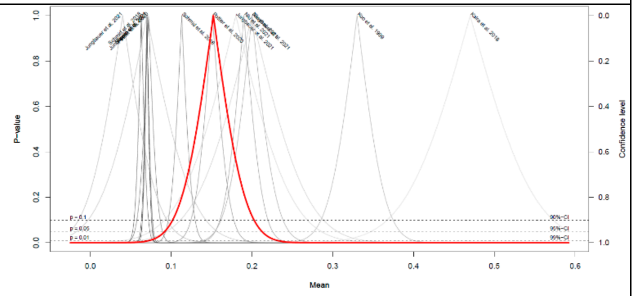 |

| | | | |
|---|---|---|---|
| Canines | 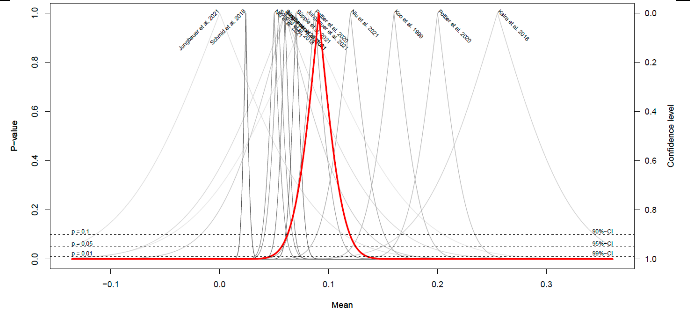  | 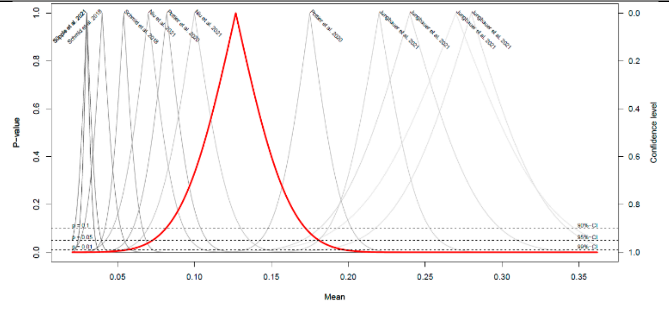  | 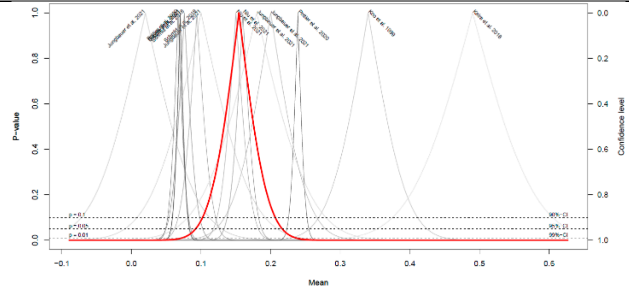  |
| Premolars | 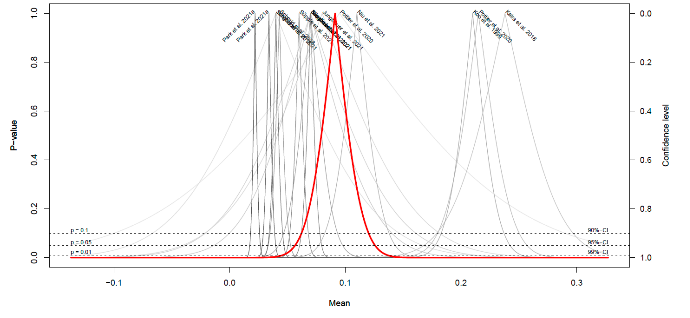  | 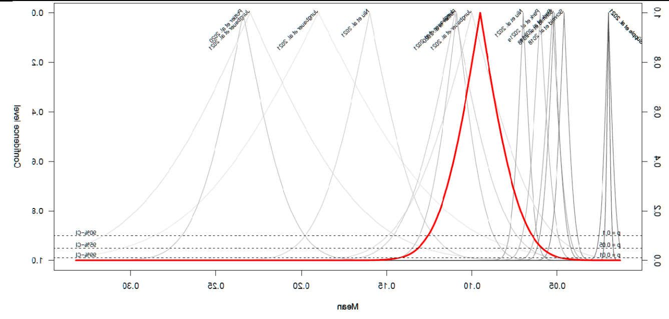 | 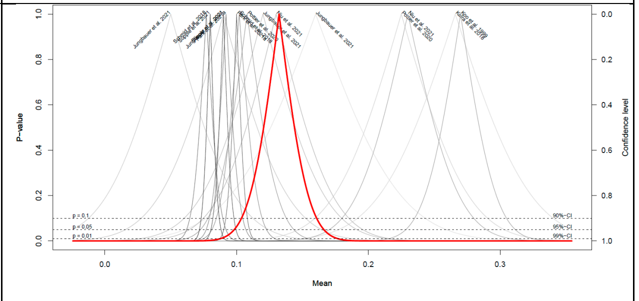  |
| Molars | 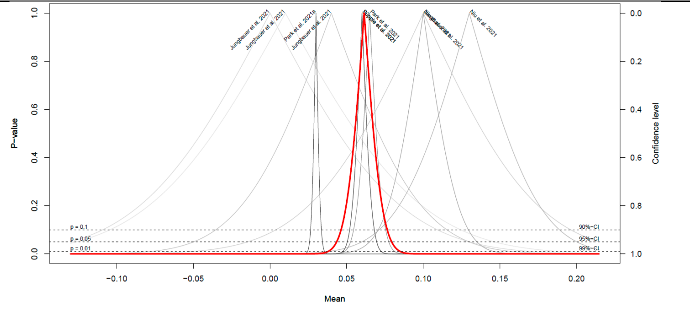  | 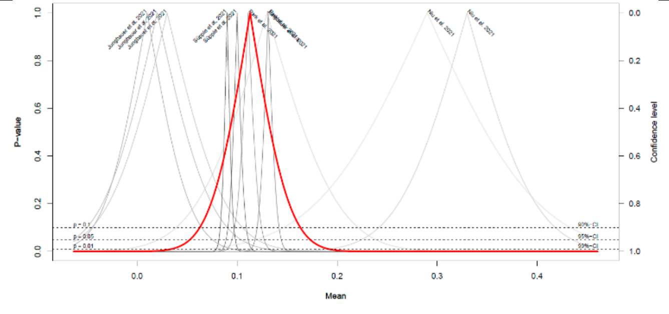  | |
